# Assessment of the relationships between agroecosystem condition and the ecosystem service soil erosion regulation in Northern Germany

**DOI:** 10.1371/journal.pone.0234288

**Published:** 2020-12-07

**Authors:** Paula Rendon, Bastian Steinhoff-Knopp, Philipp Saggau, Benjamin Burkhard

**Affiliations:** 1 Institute of Physical Geography & Landscape Ecology, Leibniz University of Hannover, Hannover, Germany; 2 Institute of Geography, Christian Albrechts University, Kiel, Germany; 3 Leibniz Centre for Agricultural Landscape Research (ZALF), Müncheberg, Germany; Chinese Academy of Forestry, CHINA

## Abstract

Ecosystems provide multiple services that are necessary to maintain human life. Agroecosystems are very productive suppliers of biomass-related provisioning ecosystem services, e.g. food, fibre, and energy. At the same time, they are highly dependent on good ecosystem condition and regulating ecosystem services such as soil fertility, water supply or soil erosion regulation. Assessments of this interplay of ecosystem condition and services are needed to understand the relationships in highly managed systems. Therefore, the aim of this study is twofold: First, to test the concept and indicators proposed by the European Union Working Group on Mapping and Assessment of Ecosystems and their Services (MAES) for assessing agroecosystem condition at a regional level. Second, to identify the relationships between ecosystem condition and the delivery of ecosystem services. For this purpose, we applied an operational framework for integrated mapping and assessment of ecosystems and their services. We used the proposed indicators to assess the condition of agroecosystems in Northern Germany and regulating ecosystem service *control of erosion rates*. We used existing data from official databases to calculate the different indicators and created maps of environmental pressures, ecosystem condition and ecosystem service indicators for the Federal State of Lower Saxony. Furthermore, we identified areas within the state where pressures are high, conditions are unfavourable, and more sustainable management practices are needed. Despite the limitations of the indicators and data availability, our results show positive, negative, and no significant correlations between the different pressures and condition indicators, and the control of erosion rates. The idea behind the MAES framework is to indicate the general condition of an ecosystem. However, we observed that not all proposed indicators can explain to what extent ecosystems can provide specific ecosystem services. Further research on other ecosystem services provided by agroecosystems would help to identify synergies and trade-offs. Moreover, the definition of a reference condition, although complicated for anthropogenically highly modified agroecosystems, would provide a benchmark to compare information on the condition of the ecosystems, leading to better land use policy and management decisions.

## 1. Introduction

Human well-being is strongly dependent on ecosystems, their biodiversity, condition, functionality and capacity to deliver multiple services. Ecosystem condition is clearly linked to ecosystem services and indicates the overall quality of an ecosystem unit in terms of its capacity to generate ecosystem services [1]. Assessing ecosystem condition can help to understand to what extent ecosystems can provide services in a sufficient quantity and quality. Some studies have focused on the links between natural capital and ecosystem services [2] and the relationships between biodiversity and ecosystem services [3]. However, understanding and quantifying the relationships between ecosystem condition and the provision of ecosystem services remains a research gap [4, 5].

International and European Union (EU) policies have integrated ecosystem condition into sustainability and conservation targets, comprising concepts such as ecosystem state, quality, status, health, integrity and functioning [6]. The Sustainable Development Goals (SDGs), for instance, were adopted by the United Nations in 2015, as “a call for action to end poverty, protect the planet and improve the lives and prospects of everyone everywhere” [7]. In particular, for biodiversity, goal 15 aims to protect, restore and promote sustainable use of terrestrial ecosystems, sustainably manage forests, combat desertification, and halt and reverse land degradation and stop biodiversity loss.

In 2011, the EU adopted the Biodiversity Strategy to 2020 [8] and established six targets to halt the loss of biodiversity and ecosystem services in the EU by 2020. These targets aim to protect species and habitats, to maintain and restore ecosystems, sustainable agriculture and use of forests, sustainable fishing and healthy seas, to fight invasive alien species and stop the loss of biodiversity. The 7^th^ Environmental Action Programme (EAP) was adopted by the EU in 2013 [9] and reinforces the targets and actions of the Biodiversity Strategy. The EAP aims to protect natural capital, stimulate resource-efficient, low-carbon growth and innovation, and safeguard human health and well-being while respecting Earth’s limits [9]. Some topics that need further action at EU and national level are the protection of soils and the sustainable use of land and forest resources.

The EU has a dedicated working group, Mapping and Assessment of Ecosystems and their Services (MAES) [10], to support the implementation of Action 5 of Target 2 of the EU Biodiversity Strategy to 2020. Action 5 requires that all Member States map and assess the state of ecosystems and their services in their territory, assess the economic value of such services and integrate these values into accounting and reporting systems at the national and EU level [8]. MAES has developed a conceptual framework [11] and suggested a series of indicators to assess the condition of different ecosystem types including agroecosystems [12]. However, these indicators still need to be tested at EU, national/sub-national and regional level; and the links with ecosystem services need further investigation. Our study tests the indicators suggested by MAES for environmental pressures, ecosystem condition and the relationships with ecosystem services on a regional level, specifically in agroecosystems in Northern Germany with a focus on the soil erosion narrative.

Agroecosystems account for almost half of the land use area in the EU [13]. In Germany, more than half of the surface area is used for agriculture [14]. Agricultural land provides, on the one hand, multiple ecosystem services, especially biomass-related services such as food, fibre, fodder or energy, which are essential for human well-being [15, 16]. On the other hand, agriculture itself is strongly dependent on ecosystem services such as nutrient regulation, water supply, pollination (for selected crop species) or soil erosion regulation [17, 18]. Changes in the condition of agroecosystems may impair the availability of these services. Environmental pressures such as soil erosion, soil biodiversity decline, soil compaction, organic matter decline, soil sealing, and contamination, together with changing climate and water regimes, degrade these ecosystems [19]. Maintaining the good condition of agroecosystems is essential to guarantee resilience, halt biodiversity loss and preserve the sustainable provision of multiple ecosystem services.

Agroecosystems are strongly modified semi-natural systems and are managed with a strong focus on provisioning services [20]. These ecosystem service outputs are, at least in conventional farming, based on substantial anthropogenic human system inputs including fertilizer, insecticides, herbicides, energy, labour and machinery use and in some cases also irrigation water [21]. Besides ecosystem service outputs, agroecosystem service delivery has significant environmental effects such as greenhouse gas emissions, biodiversity loss or water eutrophication [18].

Due to the often long-term human interference in these systems, there are difficulties in defining a (natural) reference condition of agroecosystems. Agroecosystem condition cannot only be based on the physical and ecological properties of plants and soils but must take human interventions of agroecosystems into account [16]. An agroecosystems is in good condition when it supports biodiversity and supplies multiple provisioning, regulating and cultural ecosystem services, and there is no depletion of abiotic resources such as water, soil and air [12]. Nevertheless, the establishment of threshold values to determine whether an agroecosystem is in good or bad condition is still under debate [22]. Time reference condition as, for example, before the industrial revolution and/or different reference times as in other ecosystem types are not available. Besides these temporal issues, the involvement of multiple stakeholders (farmers, policy makers, planners, consumers, environmental groups), who may have different interests and perceptions about the condition of agroecosystems [23], hampers the reference state definition.

This study focuses on the ecosystem service *control of erosion rates* and takes into account that soil erosion by water is a major problem in soil conservation in the EU [24, 25]. Soil erosion by water accounts for the largest share of soil loss in Central European agricultural ecosystems, especially in areas with steep slopes [26]. Unsuitable management activities threaten croplands by increasing the vulnerability of soils to erode [24]. The objective of this study is to conduct an integrated assessment of ecosystems for one exemplary ecosystem service in a specific ecosystem type and by using the indicators proposed by MAES. We apply an operational framework for integrated mapping and assessment of ecosystems and their services suggested by Burkhard et al. [27] in agroecosystems in the Northern German Federal State of Lower Saxony. For this purpose, we follow a stepwise approach: i) the identification of the policy objective “healthy soils”(in our study exemplified by soil erosion regulation); ii) the identification and mapping of agroecosystems; and iii) the selection, quantification and mapping of indicators of agroecosystem condition.

The main goal of this study is to test the feasibility of the indicators proposed by MAES for the assessment of agroecosystem condition at a regional level. Thereby, we hope to improve the methodology and to increase the applicability of the MAES framework and indicators. The results will be relevant for other (also non-EU/MAES-related) comparable indicator-based studies in agroecosystems, their condition and ecosystem services.

The article is organized as follows: First, we describe the methodological approach used for the integrated assessment. Then we show maps of the different indicators to evaluate the environmental pressures, ecosystem condition and the ecosystem service *control of erosion rates*. We also statistically analyse the relationships between ecosystem condition and the *control of erosion rates*. Then we discuss the main limitations of the indicators and the relationship between environmental pressures, ecosystem condition and ecosystem services and conclude with recommendations for further improvement of the MAES framework and their indicators.

## 2. Methods

### 2.1 Study area

**Fig 1:**
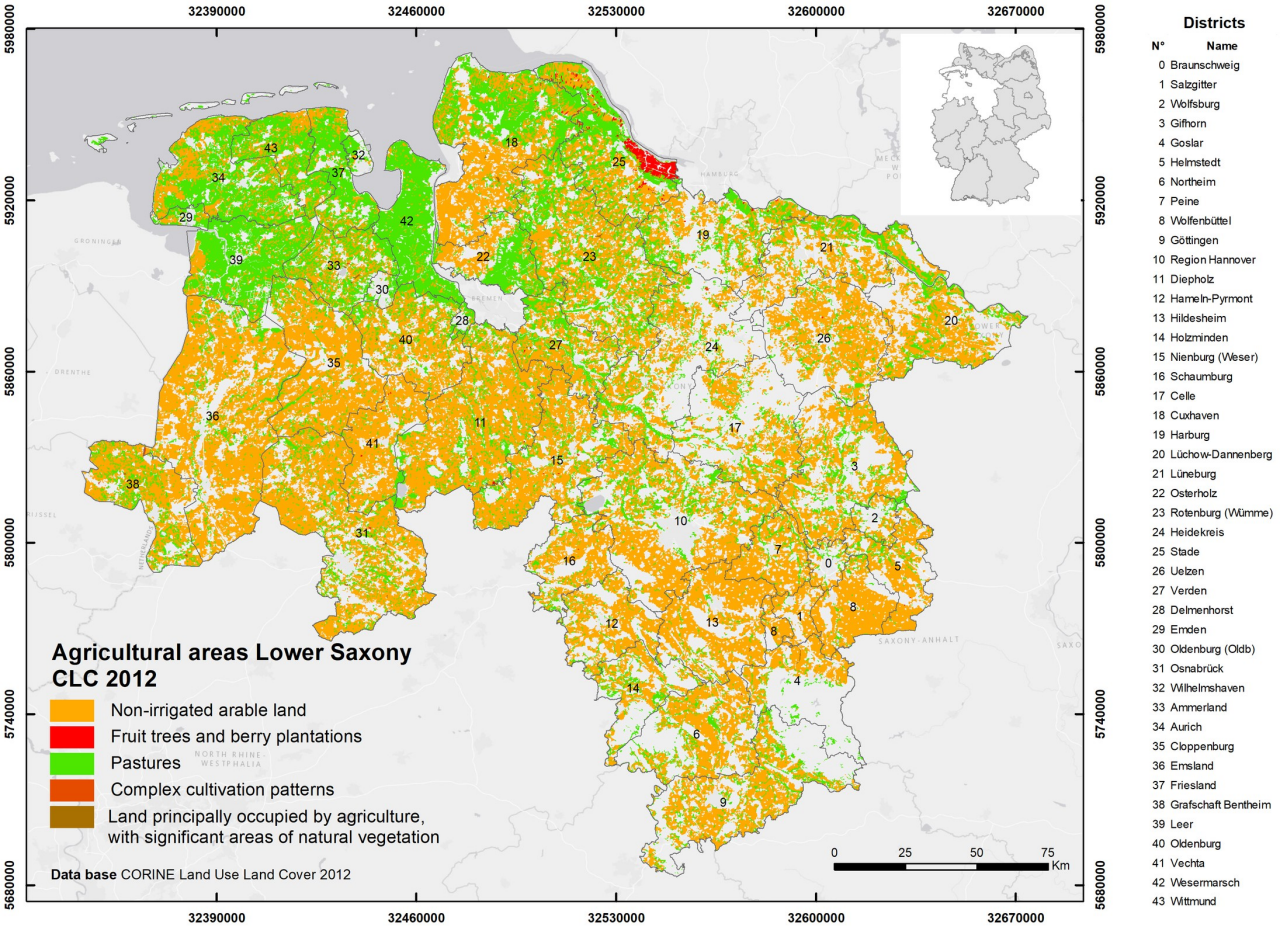
Agricultural areas in Lower Saxony. (Based on CORINE Land Use Land Cover data, of 2012 obtained from the European Environmental Agency [30] and administrative units from the German Federal Agency for Cartography and Geodesy © GeoBasis-DE / BKG (2017) [31]).

Lower Saxony is a federal state located in the north-west of the Federal Republic of Germany, adjoins the North Sea and has an area of 47,620 km^2^ (Fig 1). Its climate is characterized as sub-oceanic with average temperatures ranging from 8.3 to 9.5°C and mean precipitation values ranging from 654 mm in the south-west to 840 mm in the central and northern areas [28]. Agriculture is the main land use with 2.6 million hectares (approximately 53.7% of the total territory), of which 1.9 million hectares are arable land, 0.7 million hectares are permanent grassland and around 20,000 hectares are permanent crops [29].

More than half of the arable land in Lower Saxony is used to grow cereals (mainly winter wheat and winter barley); the remaining area is used for fodder crops, oilseeds, or potatoes. The farm sizes are very diverse and range from a few hectares of specialized horticultural businesses to large arable farms with several hundred hectares. On average, the farms have a size of 83 ha and about 75% of all farms keep animals, especially dairy cattle and pigs. Farm type, specialization and size can be used as a function of soil fertility, climate conditions and historical land use strategies [29].

### 2.2 Conceptual framework

**Fig 2:**
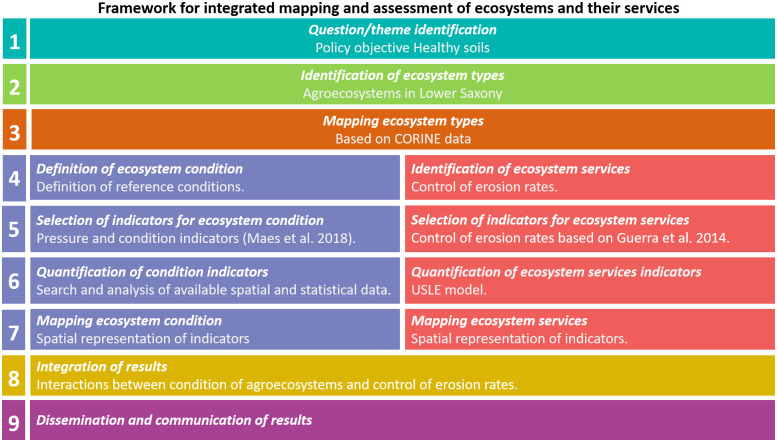
Conceptual framework applied for integrated mapping and assessment of agroecosystems and the ecosystem service *control of erosion rates*. (based on Burkhard et al. [27]).

In this study, we used the operational framework proposed by Burkhard et al. [27] that guides integrated mapping and assessment of ecosystems and their services. Fig 2 provides a summary of the framework that entails nine steps: (1) theme identification; (2) identification of ecosystem type; (3) mapping of ecosystem type; (4) definition of ecosystem condition and identification of ecosystem services to be delivered by agroecosystems; (5) selection of indicators for ecosystem condition and ecosystem services; (6) quantification of ecosystem condition and ecosystem services indicators; (7) mapping ecosystem condition and ecosystem services; (8) integration of results; and (9) dissemination and communication of results. These steps are described in detail in the next paragraphs.

### 2.3 Theme identification: Policy objective healthy soils (Step 1)

The first step of the operational framework refers to the *question and theme identification*, which must be addressed in the ecosystem assessment in order to be relevant for policy, society, business or science. In this case study, we identified the policy objective *maintaining healthy soils*. Healthy soils, especially for agriculture, have high functionality, including biodiversity, fertility and the capacity to sustainably deliver multiple ecosystem services. These services include food and fibre, climate and water regulation, water purification, carbon sequestration, nutrient cycling and provision of habitat for biodiversity [32]. At the same time, the delivery of other ecosystem services such as water supply and regulation, pollination and soil erosion regulation should not be impaired [33].

**Fig 3:**
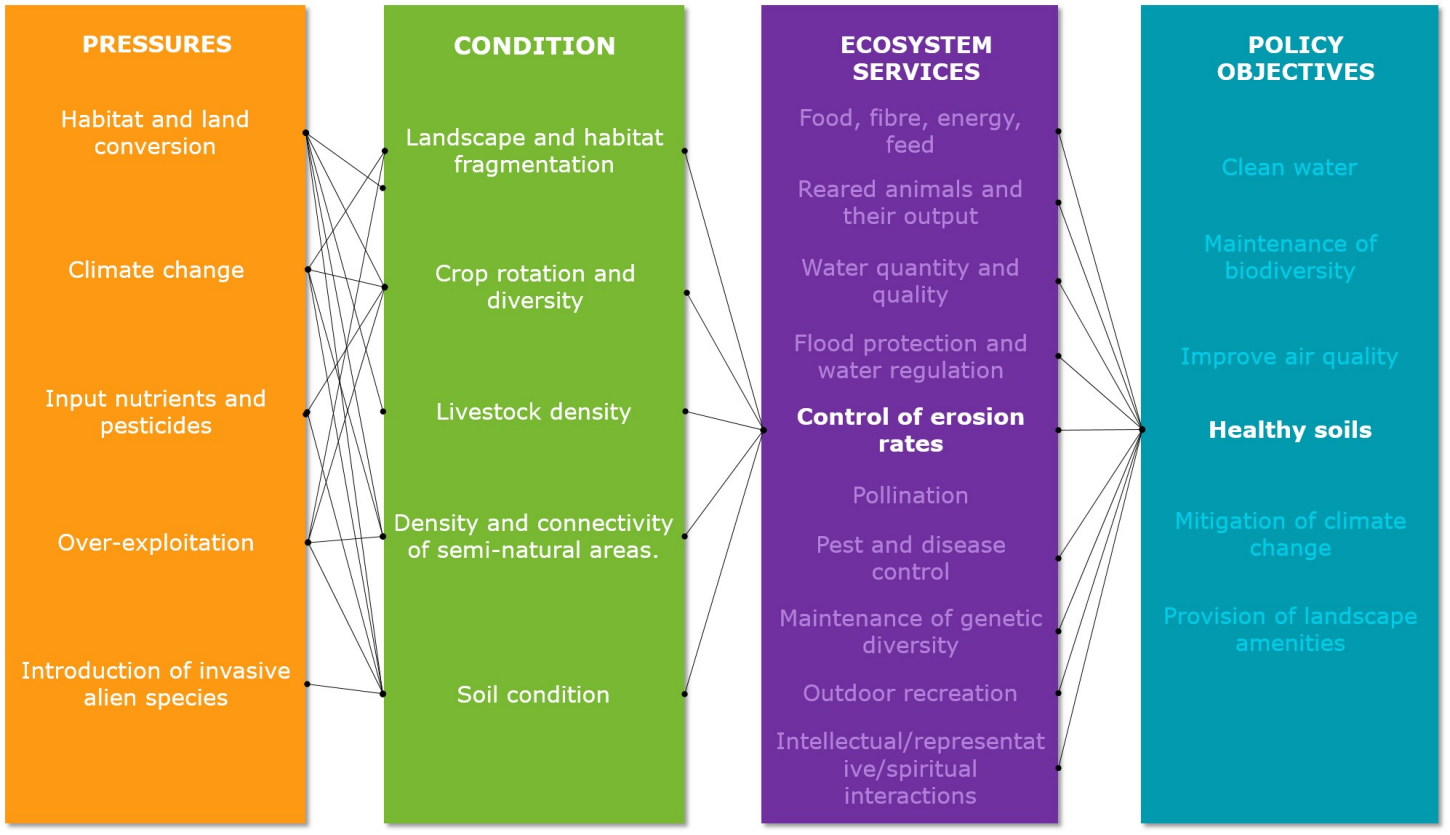
Synthesis of links between environmental pressures, condition, ecosystem service *control of erosion rates* and policy objectives in agroecosystems. (based on Maes et al. [12]).

For this study, we focus on the ecosystem service *control of erosion rates*. Fig 3 shows the condition attributes that determine the delivery of this ecosystem service. Soil condition and the presence of semi-natural areas within or in the vicinity of the agricultural fields are key for the delivery of this service [34, 35]. Additionally, livestock can affect this service by altering the structural condition of soils [36]. Crop rotations and crop types as well as the state of the landscape in which the agroecosystem is embedded are also important to control erosion rates [37]. In this case, the main typologies of environmental pressures, habitat and land conversion, climate change, input nutrients and pesticides, overexploitation, and introduction of invasive species, as proposed by Maes et al. [12], affect the condition attributes.

Healthy soils have been included as a relevant issue in national and European policies. In Germany, for instance, the objective of maintaining and preserving healthy soils was established in the Federal Soil Protection Act of March 17^th^ 1998 [38] and in the Federal Soil Protection and Contaminated Sites Ordinance of July 12^th^ 1999 [39]. Both regulations aim to sustainably secure and restore the soil functions by protecting soils against harmful changes and remediating contaminated sites. At EU level, the European Commission adopted the Thematic Strategy for Soil Protection [40] to protect soils across the EU. Although the proposal for a Soil Framework Directive was withdrawn by the Commission in 2014, the 7^th^ Environmental Action Programme (EAP) came into force in 2014 [9]. The objective of the EAP is the protection and sustainable use of soils. Under priority objective 1, the EAP states that by 2020 “land is managed sustainably in the Union, soil is adequately protected and remediation of contaminated sites is well underway”. More efforts are required to reduce soil erosion and increase soil organic matter, as well as to remediate contaminated sites.

### 2.4. Identification and mapping of the ecosystem type agroecosystems (Steps 2 and 3)

The second step of the operational framework refers to the identification of the ecosystem type(s). In this study the ecosystem type is agroecosystems which are “communities of plants and animals interacting with their physical and chemical environments that have been modified by people to produce food, fibre, fuel, and other products for human consumption and processing” [41]. Maes et al. [11] proposed a classification of ecosystem types for MAES, in which cropland and grassland belong to the ecosystem type agroecosystems. We selected cropland ecosystems in Lower Saxony because croplands are the main provider of ecosystem services such as biomass used as food, fodder or as an energy source. Croplands are threatened by mismanagement and external pressures such as droughts or floods caused by climate change. Due to management-induced bare soils during the year, cropland soils are especially affected by soil erosion.

The third step entails the mapping of the previously identified agroecosystems. For this purpose, we used the CORINE Land Cover Data for the year 2012 [42], particularly the CORINE Land Cover type 2. Agricultural areas, which includes: 211. Non-irrigated arable land, 221. Vineyards, 231. Pastures, 242. Complex cultivation patterns, and 243. Land principally occupied by agriculture, with significant areas of natural vegetation (see Fig 1).

### 2.5. Definition of ecosystem condition and identification of ecosystem services delivered by agroecosystems (Step 4)

The fourth step define the ecosystem condition and the delivered ecosystem services. Agroecosystems are usually purposely heavily modified ecosystems [18], and it is not feasible to compare their condition with undisturbed natural ecosystems. Table 1 shows the median values and the available reference values used in this study to determine the condition of the agroecosystems in Lower Saxony, based on the selected indicators.

**Table 1:** Indicators used for the assessment of environmental pressures and condition of agroecosystems and the ecosystem service *control of erosion rates* in Lower Saxony.

Indicator class	Indicator	Description	Units	Spatial resolution	Year	Median Lower Saxony	Reference value	Source
Pressure indicators								
Habitat conversion and degradation	Change in ecosystem extent	Change in the area (size) of the ecosystem within the years 2006 and 2012.	% per year	100 m	2006–2012	0	N.A.	[30]
Climate	Mean annual temperature	Annual mean of the monthly averaged mean daily air temperature in 2 m height above ground	°C	1000 m	1988–2018	9.74	N.A.	[46]
	Mean annual precipitation	Annual sum of monthly precipitation.	mm		1988–2018	765.7	N.A.	
	Drought index	Annual mean of drought index after de Martonne.	mm °C^-1^		1995–2018	37.78	Very humid (35–55) [47]	
	Precipitation 10 mm	Number of days with precipitation ≥ 10 mm per year.	Number of days		1988–2018	19.45	N.A.	
	Precipitation 20 mm	Number of days with precipitation ≥ 20 mm per year.				3.77	N.A.	
	Precipitation 30 mm	Number of days with precipitation ≥ 30 mm per year.				0.91	N.A.	
	Beginning of vegetation period	Number of consecutive days of the year. It indicates the beginning of the first spring	Consecutive days of the year		1992–2018	82.68	N.A.	
	Summer soil moisture	Modelled trend in soil moisture over a depth of 60 cm.	% NFK (usable field capacity)		1991–2010	66.82	N.A.	
Others	Soil erosion	Amount of soil loss per hectare in a year (Actual soil loss)	t ha^-1^ per year	50 m	2010	0.13	0–3 [48]	[49]
	Loss of organic matter	Percentage of soil organic carbon loss per year.	Mg C ha^-1^ per year	1000 m	2013	0.015	N.A.	[50–52]
Ecosystem condition indicators								
Structural ecosystem attributes (general)	Crop diversity	Average number of crops in a 10 km diameter per municipality	Number of crops	50 m	2018	44.41	N.A.	[53]
	Density of semi-natural areas	Percentage of semi-natural areas	%	50 m	2012	13.16	N.A.	[42]
	Share of fallow land in Utilized Agricultural Area (UAA)	Percentage of arable land that is not being used for agricultural purposes within the UAA.	%	Municipality	2010	0.80	N.A.	[54]
	Share of arable land in Utilized Agricultural Area (UAA)	Percentage of land used for the production of crops within the UAA	%	Municipality	2010	81.32	N.A.	[54]
	Share of permanent crops in Utilized Agricultural Area (UAA)	Percentage of land used for permanent crops within the UAA	%	Municipality	2010	0	N.A.	[54]
	Livestock Density	Stock of animals (cattle, sheep, goats, equidae, pigs, poultry and rabbits) converted in livestock units (LU) per hectare of UAA.	LU ha^-1^	Municipality	2010	0.81	N.A.	[54]
Structural soil attributes	Soil Organic Carbon (SOC)	Concentration of topsoil organic carbon.	% or gr kg^-1^	1000 m	2010	2.03	1–2% [55]	[56]
	Soil erodibility	Susceptibility of soil to erosion by runoff and raindrop impact.	K factor [t ha^-1^N^-1^]	50 m	2010	0.21	N.A.	[56, 57]
	Bulk density	Weight of soil per cubic meter	t m^-3^	500 m	2015	1.3	1.6 g cm^-3^ for sandy and sandy loam soils 1.75 g cm^-3^ for coarse textured soils (with clay content < 17.5%) [55]	[58, 59]
Ecosystem services indicators								
Control of erosion rates	Soil erosion risk	Potential soil loss.	t ha^-1^ per year	50 m	2010	0.81	N.A.	[49]
	Prevented soil erosion	Difference of potential and actual soil loss	t ha^-1^ per year	50 m	2010	0.67	N.A.	[56]
	Provision capacity	Share of mitigation of soil erosion (0 to 1)	Dimensionless	50 m	2010	0.85	From 0 (no mitigation) to 1 (complete mitigation)	[56]

We selected the ecosystem service *control of erosion rates*, because soil erosion is one of the main threats to soils [40] with negative impacts on crop production, water quality, mudslides, eutrophication, biodiversity and carbon stock loss [26]. Soils are the medium on which crops are grown and their functionality is the base for biomass production, storage, filtration and transformation of nutrients and water. Furthermore, healthy soils are essential for biodiversity conservation and act as carbon storage pools. Soils are the platform for human activities, provide raw materials and store geological and archaeological heritage [43]. Soil degradation leads to the decline of many ecosystem services [44] and soil erosion decreases soil surface and then soil thickness. Especially the loss of the humus-rich, fertile topsoil layers leads to a reduction of soil functionality and the capacity to provide ecosystem services. If these losses are not compensated by soil formation, soil erosion will threaten sustainable crop production as well as water regulation and filtration capacities [45].

### 2.6. Selection of indicators for agroecosystem condition and the ecosystem service *control of erosion rates* (Step 5)

The fifth step refers to the selection of the indicators for the assessment of ecosystem condition and ecosystem services. As this study aims to test the framework and indicators proposed by MAES, we chose the indicators for pressures and condition of agroecosystems presented in the 5th MAES report [12]. We selected the indicators for the ecosystem service *control of erosion rates* based on existing literature and the frameworks used by Guerra et al. [60] and Steinhoff-Knopp and Burkhard [25].

#### 2.6.1. Criteria for selecting indicators

Ecosystem condition indicators allow us to assess the overall quality of an ecosystem and its main characteristics that underpin its capacity to deliver ecosystem services [1]. These indicators, together with information on ecosystem extent and services, constitute the main inputs for integrated ecosystem assessments that analyse the links between ecosystem condition, habitat quality and biodiversity, ecosystem services, and the consequences for human wellbeing [27].

Maes et al. [12] highlighted the main characteristics that ecosystem condition indicators must have to inform policies related to the use and protection of natural resources. First, ecosystem condition indicators need to be aligned with the MAES framework in which socioeconomic systems are linked with ecosystems through the flow of ecosystem services and the drivers that affect ecosystems. Second, they need to support the objectives of the EU environmental legislation and the objectives of the natural capital accounts. Third, they need to be policy-relevant, which means that they support EU environmental policies, related national policies and any other policies. Fourth, they need to be spatially explicit by considering the distribution of ecosystems and their use, and they need to be specific for each ecosystem type. Fifth, they need to contribute to measuring progress/trends against a policy baseline towards different policy targets.

We therefore, adopted the following criteria for selecting the indicators.

- Relevancy: Indicators are relevant to the ecosystem service *control of erosion rates*. There is a clear connection between the condition parameter and the provision of the ecosystem service. This connection was determined based on the authors’ expertise and scientific literature.
- Availability of data: The data have an appropriate resolution for the study area and are detailed enough to recognize regional features (i.e. spatially explicit or data at the level of municipalities).
- Quantifiable: Indicators are quantifiable and data can be compared among municipalities.
- Reliability: Both the quantification and monitoring of the indicators are reliable (i.e. data obtained from officially reported data sets).

A total of 23 indicators was selected for the assessment, whereof 11 correspond to pressure (including seven climate indicators), nine correspond to ecosystem condition and 3 to the ecosystem service *control of erosion rates*.

### 2.7. Quantification of indicators (Step 6)

The sixth step refers to the quantification of indicators for condition and the ecosystem service *control of erosion rates*. We conducted a data search in several public databases of the EU, Germany and Lower Saxony. Table 1 provides detailed descriptions of the indicators, including the spatial resolution, year of data collection and data sources. We used the best data available concerning temporal and spatial resolution, which may not be optimal for this kind of study as discussed in section 4.1. We describe the calculation of each indicator in detail in S1 File.

### 2.8. Mapping of indicators (Step 7)

This step refers to the spatial visualisation of the ecosystem condition and the ecosystem service *control of erosion rates* indicators in maps. All the indicators were edited in ArcGIS 10.7 for representation and analysis. They were aggregated to the level of the municipalities to facilitate their comparison.

### 2.9. Integration of results (Step 8)

The integration of results refers to the analysis of relationships and interactions between the condition of agroecosystems and the supply of the ecosystem service *control of erosion rates*. This analysis was conducted from two different angles: the assessment of statistical correlations and the analysis of the spatial distributions and relationships from the compiled maps.

For the statistical correlations, seven classes of the ecosystem service *control of erosion rates* were selected, based on the classification proposed by Steinhoff-Knopp and Burkhard [25] for the potential soil erosion by water on croplands in Lower Saxony. The classes are *No to very low* (less than 1 t ha^-1^ per year eroded soil), *very low* (1 to 5 t ha^-1^ per year), *low* (>5 to 10 t ha^-1^ per year), *medium* (>10 to 15 t ha^-1^ per year), *high* (>15 to 30 t ha^-1^ per year), *very high* (>30 to 55 t ha^-1^ per year) and *extremely high* (≥ 55 t ha^-1^ per year). We considered the environmental pressures and ecosystem condition indicators per class, which are also used to classify the actual *control of erosion rates*. We applied the Akaike Information Criterion (AIC) [61] technique to estimate the likelihood of the different pressure and condition indicators to predict the values of the seven *control of erosion rates* classes mentioned above. Additionally, we applied the Kruskal-Wallis rank test [62] on the class medians to detect significant differences between the seven classes. We carried out the Jonckheere-Terpstra test [63, 64] to identify positive or negative relationships between the delivery of the ecosystem service *control of erosion rates* and the environmental pressures and ecosystem condition. The statistical work was conducted in RStudio (version 1.2.1335) [65].

To show an integrated overview of the distribution of the ecosystem service *control of erosion rates*, the potential soil loss, and the pressures and condition in Lower Saxony, we normalized all the variables and standardized them to a 0 to 100 scale to make them comparable. When looking at pressures and condition variables, we considered the presumable effect of each of them based on the supply of the ecosystem service *control of erosion rates* to do the normalization. The pressure indicators *drought index* and *summer soil moisture*, for instance, are thought to have a positive effect on the supply of the ecosystem service. Regarding the drought index (Ia DM) in regions classified as arid (Ia DM<10) and semi-arid (10≤ Ia DM<20) in the *index of the Martonne*, the protective cover provided by plants against rain splash decreases with increased aridity [66]. This means that the higher the value of the drought index, the lower the erosion risk. Similarly, higher soil moisture maximizes vegetation cover, resulting in the minimization of sediment transport capacity, with important differences between clay and sandy soil textures [67]. On the other hand, the condition indicators *fallow land*, *livestock density* and *soil erodibility (K factor)* have a presumably negative effect on the *control of erosion rates*. Poor structural stability, as well as less plant cover in fallow systems result in an increased erosion risk [68], and a higher percentage of fallow land, indicating areas with bare soil, increases the soil erosion in a specific area. Likewise, the trampling of livestock disturbs and loosens soil, which makes it easier for soil to be removed by agents of transport and therefore increases erodibility [36]. These five indicators were multiplied by -1 in order to take into account the positive or negative effects when normalizing the original data. We then calculated the average of the normalized values for the pressure and condition indicators.

To spatially visualize the relationships between the indicators, we created maps showing the overlaps of pressures, condition, soil erosion risk, and provision capacity. This analysis allowed us to identify how pressures and condition related to soil erosion risk and the provision capacity of the agroecosystems are able to control soil erosion.

### 2.10. Dissemination and communication of results (Step 9)

This step refers to the preparation of maps and other accompanying material for effective dissemination and communication of the results. According to Burkhard et al. [27], the results must be communicated to potentially interested decision makers and other stakeholders in order to answer the initial question(s) posed in Step 1. However, for this assessment, we did not involve stakeholders and these results have not been communicated.

## 3. Results

The results of the assessment are presented in maps showing the distribution of the indicators of environmental pressures, ecosystem condition and the ecosystem service *control of erosion rates* within Lower Saxony. Other graphs and maps show the integration of results and the relationships between pressures, condition and *control of erosion rates* (see data per municipality in S2 File).

### 3.1. Mapping and assessment of agroecosystem condition in Lower Saxony

#### 3.1.1 Pressure indicators

**Fig 4:**
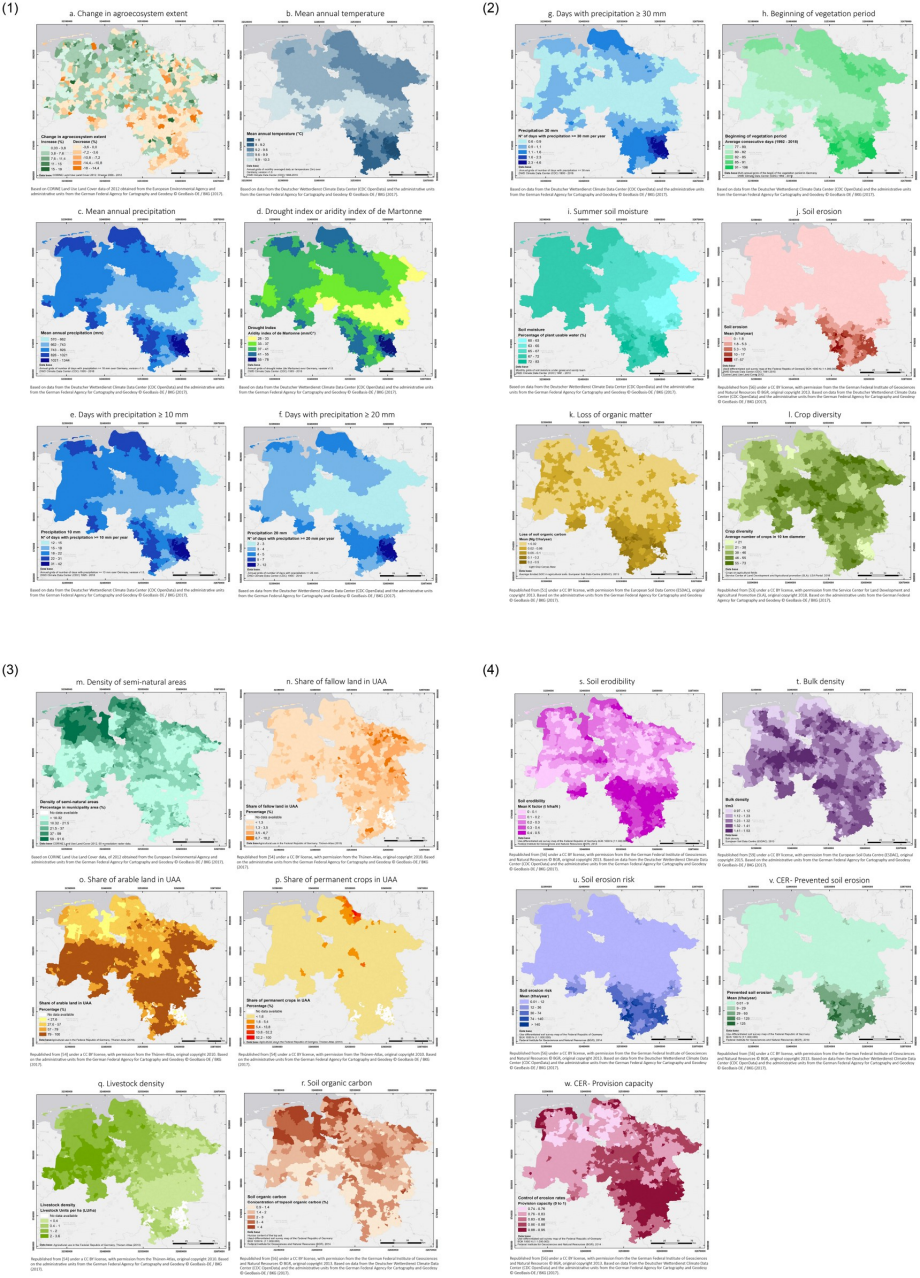
Maps of indicators of environmental pressure, ecosystem condition and control of erosion rates in Lower Saxony. (Larger maps are provided in the S3 File).

*Change in ecosystem extent*. Agroecosystems in Lower Saxony did not experience significant changes in extent from the year 2006 to 2012. Most of the municipalities showed changes from 0% to +/- 0.7%. The changes were more significant in Amelinghausen (district of Lüneburg), in the north-east, with an increase of 19%; Himmelpforten (Stade), in the north, (17,4%); and Seedorf (Rotenburg -Wümme), also in the north with an increase of 16,5%. On the other hand, there were significant reductions in the size of agroecosystems in Spelle (Emsland) in the west and Wallmoden (Goslar) in the south-east, both with a decrease of 18% (Fig 4a).

*Climate*. *Mean annual temperatures* in Lower Saxony ranged from 6.7 °C to 10.3 °C, with the lowest temperatures recorded in mountainous areas such as the Harz (district of Goslar) in the south-east (Fig 4b).

*Mean annual precipitation* in Lower Saxony ranged from 570 mm in the east to 1344 mm in the mountainous region located in the south-east. Precipitation in the coastal areas and mountainous regions in the south ranged from 826 mm to 1021 mm (Fig 4c).

*Drought index or aridity index of the Martonne* ranged from humid (28) to extremely humid (78) across the study area. Municipalities located in mountainous regions (Braunlage and Clausthal-Zellerfeld in the district of Goslar) had the lowest aridity together with areas with continental weather (near the state of Saxony Anhalt and some areas close to North-Rhine Westphalia) (Fig 4d).

The number of days with precipitation higher than 10, 20 and 30 mm was negatively correlated with the amount of rainfall that was recorded. For instance, the number of days with precipitation between 10 and 20 mm ranged from 2 to 42 days, while the number of days with precipitation between 20 mm and 30 mm ranged from 0.6 to 4.6 days (Fig 4e–4g). Similarly to other climatic parameters, higher precipitations occurred in mountainous and coastal regions.

The *beginning of the vegetation period* in Lower Saxony ranged from 77 to 106 consecutive days of the year during the period between 1992 and 2018 across the different municipalities. The areas with the latest beginning of spring are located in the mountainous regions (Braunlage and Clausthal-Zellerfeld in the district of Goslar), which is related to the prevalence of lower temperatures throughout the year (Fig 4h).

*Summer soil moisture* in Lower Saxony was in line with the precipitation levels showing percentages of plant-available water ranging from 60% in the eastern area to 83% in the south-east (mainly mountainous regions) (Fig 4i).

*Soil erosion* had on average the highest values in the southern mountainous region (56.9 t ha^-1^ per year in the municipality of Wenzen, district of Holzminden), whereas municipalities located in the Lower Saxonian German Plain in the northern half of the state, showed median values below 1.8 t ha^-1^ per year of eroded soil (Fig 4j).

*Loss of organic matter* had the highest values in the southern region (0.48 Mg C ha^-1^ per year, in Braunlage, district of Goslar). Other municipalities in the east and north showed values ranging from 0.06 to 0.1 Mg C ha^-1^ per year. The median for the whole federal state was 0.02 Mg C ha^-1^ per year (Fig 4k).

#### 3.1.2 Ecosystem condition indicators

*Crop diversity* varied greatly across Lower Saxony. North-eastern and central areas had the highest values, ranging from 55 to 73 crop species. On the other hand, the North Sea islands in the north-west and the mountainous area in the district of Goslar in the south-east had values below 21 crop species. (Fig 4l).

The *density of semi-natural areas* was higher in the north-west of Lower Saxony, with percentages as high as 91% in the municipality of Ovelgönne (district of Wesermarsch) and more than 87% in Engelschoff (district of Stade), mostly represented in the form of pastures. Almost 40% of the municipalities distributed in the southern and north-eastern regions had less than 10% of semi-natural areas and feature intensive agriculture, mainly with cereal crops (Fig 4m).

The *share of fallow land in UAA* was relatively low in Lower Saxony, with a median of 0.86% for the whole federal state. Almost 60% of the municipalities had a percentage of fallow land below 1.3%, whereas only 41 municipalities had values higher than 6.7%, mainly located in the eastern region (Fig 4n).

The *share of arable land in UAA* was higher in areas with a lower density of semi-natural elements, which is in line with theoretical expectations, where most regions with intensive agriculture tend to have low values of semi-natural vegetation [69]. Municipalities with a share of arable land of around 80% were mainly distributed in the east and south-west of the federal state, where the production of crops such as grains and wheat was high (Fig 4o).

*Share of permanent crops in UAA* was low in Lower Saxony, with only five municipalities located in the district Stade in the north, with a share higher than 80%. This region, “Altes Land”, has mostly tree and berry fruits and fruit tree plantations. The median share of permanent crops for the federal state was 0%, since only 175 municipalities out of the 959 in the study have values higher than 0% (Fig 4p).

*Livestock density* was higher in the western part of Lower Saxony with the highest number of livestock units per hectare (LU ha^-1^) in the districts of Vechta and Cloppenburg with values between 2 and 3.6 LU ha^-1^. Values smaller than 0.4 LU ha^-1^ were recorded in the eastern part of the state and on some islands in the north-west. The median livestock density of the federal state was 0.8 LU ha^-1^ (Fig 4q).

*Soil Organic Carbon* concentrations were high in the north-west of the state with levels of topsoil organic carbon higher than 4% (Fig 4r). This concurs with large peatland areas in north-western Germany. The levels were higher than the threshold values between 1 and 2% as estimated by Kibblewhite et al. [55]. On the other hand, levels of soil organic carbon in the eastern region of the study area, especially in the district of Wolfenbüttel, were lower than 0.9%, which could be an indication of potential degradation.

*Soil erodibility* (K factor) values ranged from 0 to 0.5 t h ha^-1^ N^-1^. Some municipalities in the south-east and the north-west had higher mean values (Fig 4s). Additionally, the topsoils in these areas had high contents of silt, which makes them highly erodible [24].

*Bulk density* in Lower Saxony ranged from 0.97 t m^-3^ to 1.53 t m^-3^, distributed throughout all the municipalities (Fig 4t). These values were lower than the threshold levels for sandy and sandy loam soils of 1.6 g cm^-3^ estimated by Huber et al. [48].

#### 3.1.3 Ecosystem service indicators

*Soil erosion risk* showed high values in the south-east part of the study area with values higher than 140 t ha^-1^ per year, which are considered extremely high according to Steinhoff-Knopp & Burkhard [25]. However, the median value of the entire state was calculated to be 0.8 t ha^-1^per year, and some municipalities even showed mean values as low as 0.01 t ha^-1^ per year (Fig 4u).

*Prevented soil erosion* showed a concentration of high values mainly in the south-east (Fig 4v). As previously mentioned, this area also showed high values of potential and actual soil losses. However, the actual soil loss was considerably lower than the calculated soil loss potential, resulting in a high ecosystem service provision in this area.

*Provision capacity* was relatively high in Lower Saxony with values ranging from 0.74 to 0.95 across all municipalities (Fig 4w). The mean value of 0.85 indicates that most parts of the study area are protected against soil erosion.

### 3.2. Relationships between agroecosystem condition and control of erosion rates

#### 3.2.1 Analysis of the relationships between indicators

**Fig 5:**
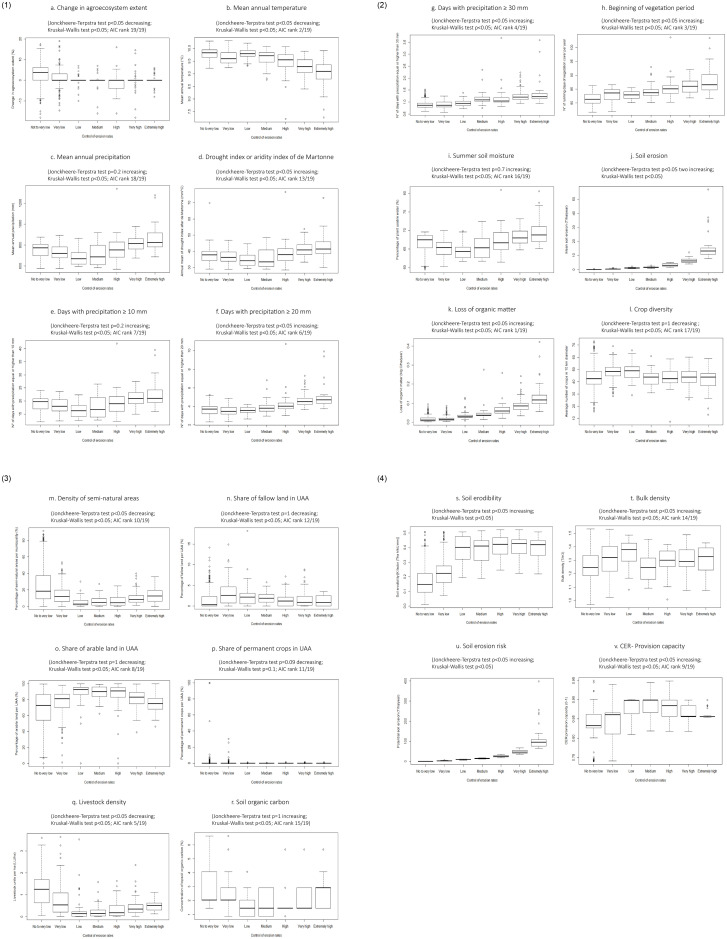
Relationships between the indicators of environmental pressures and condition, and the ecosystem service *control of erosion rates*.

In order to understand the relationships between agroecosystem condition and supply of the ecosystem service *control of erosion rates*, we analysed the likelihood of the different indicators (excluding soil erosion, soil erosion risk, and soil erodibility which were partly included in the calculation of the ecosystem service) to predict the classes from *no to very low* to *extremely high* mentioned in section 2.9. Our results show that the indicators that best predicted these classes are: *loss of organic carbon*, followed by *mean annual temperature* and *beginning of vegetation period*. In contrast, the indicators that had the lowest likelihood to predict the classes were *mean annual precipitation* and *crop diversity* (see AIC rank on Fig 5 and S1 Table).

Additionally, we analysed the relationships between pressures and ecosystem condition and the ecosystem service *control of erosion rates* (see S1 Table). Our results show that the rates of control of erosion were slightly higher in areas with increased *ecosystem extent* (p < 0.05) (Fig 5a). For the climatic variables, we found negative, positive as well as not significant correlations. For instance, the *control of erosion rates* was extremely high in areas with lower temperatures (p < 0.05) (Fig 5b). On the other hand, high and extremely high *control of erosion rates* occurred in areas where variables such as *drought index*, *days with precipitations higher than 20 and 30 mm* and *beginning of vegetation period* were high (p < 0.05). However, the relationships between *mean annual precipitation*, *days with precipitation higher than 10 mm*, *summer soil moisture* and the *control of erosion rates* were not significant (p = 0.2), (p = 0.2), and (p = 0.7), respectively (Fig 5c–5i). Higher values of *soil erosion* and *loss of soil organic matter* occurred in ecosystems providing higher *control of erosion rates* (p < 0.05) (Fig 5j and 5k). In contrast, the *density of semi-natural areas* and *livestock density* were low where the *control of erosion rates* was high or very high (p < 0.05) (Fig 5m and 5q). Moreover, there was no significant relationship between *crop diversity*, *fallow land*, *arable land*, and *soil organic carbon* with the *control of erosion rates* (p = 1) (Fig 5l and 5n–5r). The condition indicators *soil erodibility*, *bulk density*, *soil organic carbon* and the ecosystem service indicators *soil erosion risk* and *provision capacity* showed a positive relationship with the *control of erosion rates* (p < 0.05) (Fig 5s–5v).

#### 3.2.2 Overlaps between environmental pressures, ecosystem condition, soil erosion risk and ecosystem service provision capacity

**Fig 6:**
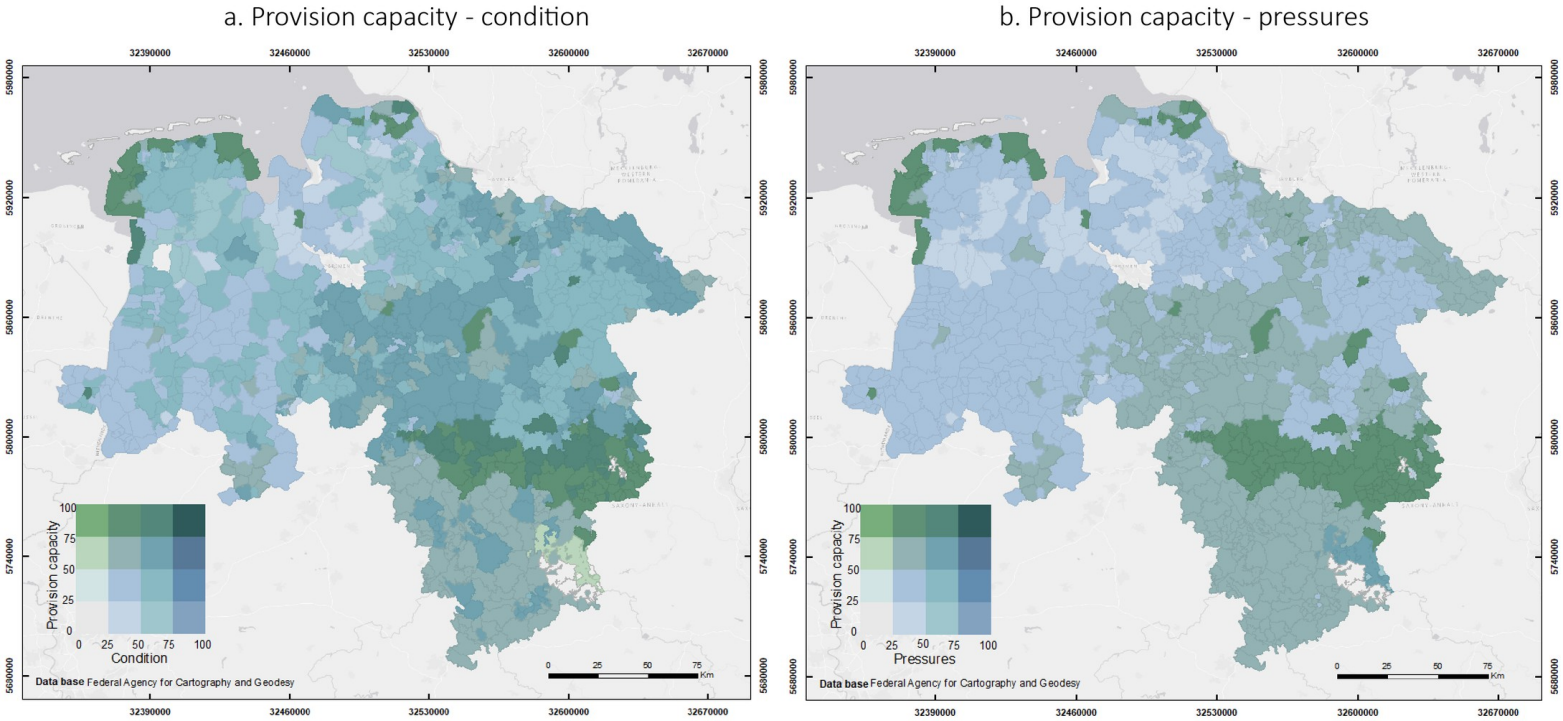
Spatial representation of the overlap between environmental pressures and condition, and ecosystem service provision capacity. (a) Overlap between provision capacity and condition. (b) Overlap between provision capacity and pressures. Darker areas represent high provision capacity and high level of condition in (a), or high provision capacity but high pressures in (b). Lighter areas represent lower provision capacity and low level of condition in (a) or lower provision capacity and low pressures in (b). Based on data from the administrative units from the German Federal Agency for Cartography and Geodesy © GeoBasis-DE / BKG (2017) [31]. (Larger maps in S3 File).

Fig 6 shows the spatial distribution of environmental pressures and condition in relation to the ecosystem service *provision capacity* in Lower Saxony. Fig 6a shows the superimposition of the normalized values of condition and provision capacity. Areas with high provision capacity and high condition levels (darker colours on the right top corner) were mainly located in the north-western and central regions. High provision capacity was also found in municipalities with medium condition levels located mainly in the southern and eastern regions (dark blue). High provision capacity was not necessarily associated with a high level of ecosystem condition in municipalities such as the Harz in the south-east (light green). However, this area is mostly covered by mountainous forest and is part of a national park. On the contrary, some municipalities in the north-west showed low provision capacity, but medium condition levels (light blue). No municipalities in Lower Saxony had low provision capacity and low condition levels.

The spatial distribution of provision capacity and pressures shows that most of the study area had medium levels of pressures and medium or high provision capacity (darker blue colours) (Fig 6b). The highest provision capacity occurred in the central and north-western regions where the pressures had a medium level (dark green). Low provision capacities and medium pressure levels occurred in the north-west, especially in some municipalities of the districts of Cuxhaven, Rotemburg (Wümme) and Wesermarsch.

**Fig 7:**
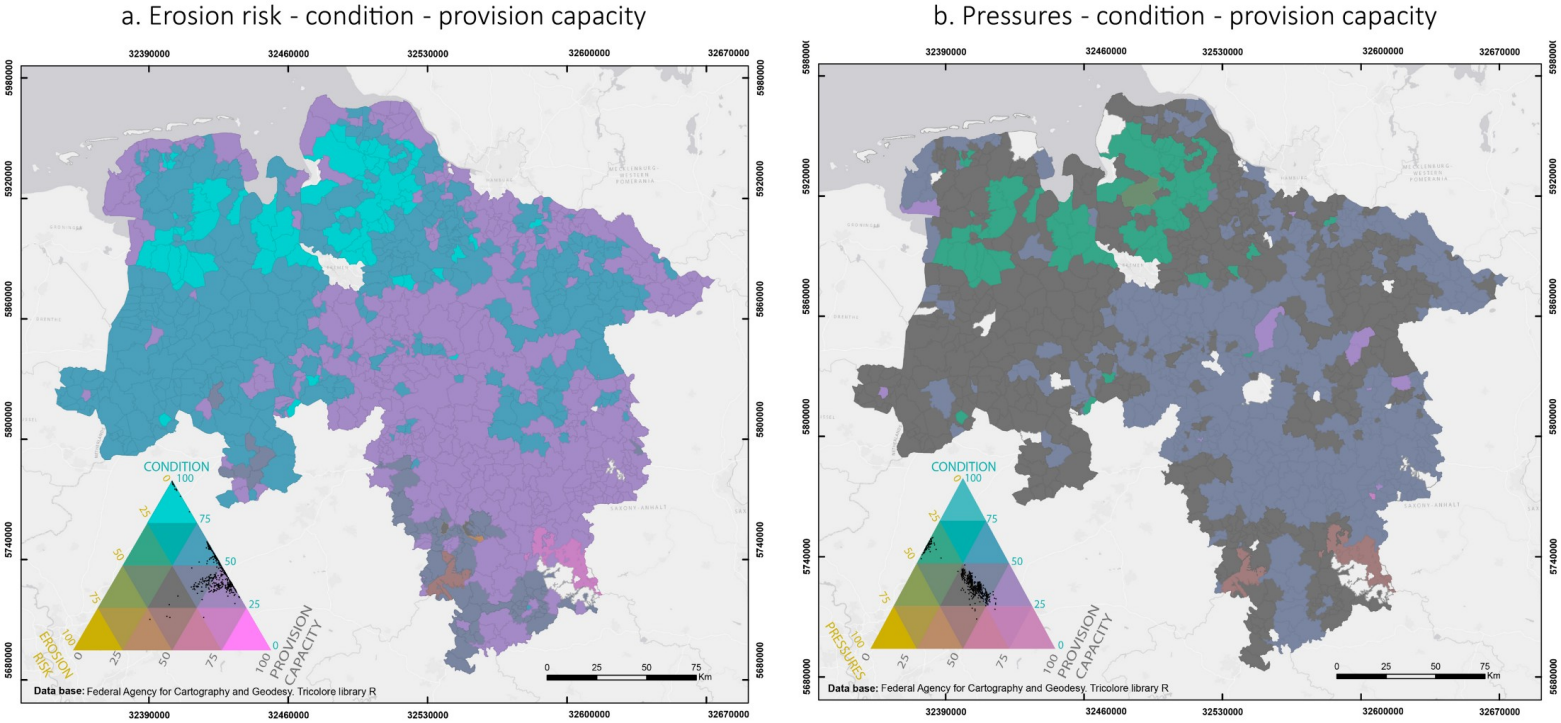
Spatial representation of the overlap between environmental pressures, condition, soil erosion risk and ecosystem service provision capacity. (a) Overlap between soil erosion risk, condition and provision capacity. (b) Overlap between pressures, condition and provision capacity. The colours and dots in the triangle show the distribution of the municipalities across the different indicators in percentage. Darker areas represent medium values of all the variables. Dots close to the lower right corner indicate high values of provision capacity. Dots close to the lower left corner indicate high erosion risk in (a) and high pressures in (b). Dots close to the upper corner indicate high condition levels. Based on data from the administrative units from the German Federal Agency for Cartography and Geodesy © GeoBasis-DE / BKG (2017) [31]. (Larger maps in S3 File).

Fig 7 shows the spatial distribution of soil erosion risk, environmental pressures and condition in relation to the ecosystem service provision capacity in Lower Saxony. Fig 7a shows the superimposition of the normalized values of soil erosion risk, condition and provision capacity. Areas with high provision capacity, high condition levels, and low erosion risk (top of the triangle) were mainly located in the north-western region. High provision capacity was also found in municipalities with medium condition levels and low erosion risk located mainly in the western region and (to a lesser extent) in the north-east (blue colour on the right side of the triangle) and the south (grey colour on the right side of the triangle). Medium provision capacity, condition and erosion risk were found in the district of Holzminden in the south (grey colour in the middle of the triangle). On the other hand, high provision capacity was evident in areas with medium-low condition levels and medium erosion risk (plum colours on the right side of the triangle). These mismatches were evident in the north-western and western regions. Medium provision capacity, low condition levels and high erosion risk (tan colour at the bottom of the triangle) were evident in the southern region, especially in the municipality of Wenzen, also in the district of Holzmiden.

The spatial distribution of provision capacity, pressures and condition shows that most of the study area had medium levels of pressures, medium condition levels and medium or high provision capacity (grey colours in the middle and on the right side of the triangle) (Fig 7b). The highest provision capacity was evident in the districts of Northeim and Goslar, but the condition levels in these areas were low and the pressures were medium. The lowest provision capacity occurred in the north-western region where the pressures had medium levels and condition had high levels (green colour on the left side of the triangle).

## 4. Discussion

The analysis based on the operational MAES framework proposed by Maes et al. [12] and the complied maps provide good results concerning the relationships between ecosystem condition and soil erosion regulating ecosystem service in Northern Germany. In the following, we will discuss the limitations of the applied indicators and provide recommendations for improved applications.

### 4.1. Limitations of the indicators for ecosystem condition and control of erosion rates

#### 4.1.1 Data availability

One aim of this study was to test the conceptual framework for assessing ecosystem condition as suggested by the MAES working group on a regional level. As shown for the soil erosion narrative, the availability and accessibility of spatial and statistical data is a major obstacle when quantifying specific indicators and thus limits the application and explanatory power of such assessment studies. We identified different reasons for data *unavailability/inaccessibility*:

1. Absolute unavailability: Data do not exist.
2. Relative unavailability: Data are not freely available (inaccessibility).
3. Spatial mismatch: Data are only available or exist for highly aggregated areas.
4. Temporal mismatch: Data are not available for the study timeframe.

For some proposed indicators, data simply do not exist *(absolute unavailability)*. In our case study region, for instance, data on crop rotation and soil biodiversity were never collected. As the MAES working group proposes a high number of indicators of which some are very detailed such as fertilizers and pesticides use, this obstacle will certainly occur in other EU regions and for other indicators. Besides, data for model-testing on a regional case is usually not available due to the high costs and time required for measuring (in contrast to catchment or plot scales) [70].

The *relative unavailability* is due to the circumstance that environmental and spatial data collected by public authorities is not freely accessible. A trend exist to make data freely available [71, 72]. Regulations on the EU level promote free data availability [73], but compete with national (or federal state) data protection laws. For instance, in our case study region, some statistical data on the municipality level for crop rotation were not available due to German data protection regulations.

*Spatial mismatches* are well documented issues in environmental studies [74] and occur mainly from different spatial resolutions of geodata or mismatched administrative units [75]. Rescaling and aggregating data to one spatial resolution is a valid and common scientific practice [76–78] and was also implemented in this study for indicators related to climate, soil characteristics, and soil erosion. This makes data comparable without improving the information density of lower resolution data. However, the actual explanatory power of downscaled data is not as high as the provided spatial resolution would suggest. Spatial mismatches occur especially in studies that apply a high number of indicators. The proposed MAES framework and our case study are excellent examples of this issue.

*Temporal mismatches* are also a well-known problem [79]. They arise mainly from mismatching survey cycles and emerge often in combination with relative unavailability of data. Assessing the temporal variations of data is key to handle temporal mismatches [75]. This way, comparable timeframes can be identified and data can be included in environmental studies.

#### 4.1.2 Environmental pressures indicators

*Change in ecosystem extent* was calculated, between the years 2006 and 2012. This is the timeframe of the CORINE land use change layers that was closest to the reference year we used to calculate the soil erosion. However, this two-year comparison leads to some limitations when assessing trends because this timespan does not provide enough information to draw precise conclusions. Additionally, the indicator, as it is proposed here, only shows the degree of change, but not the drivers of change. So when comparing it with an ecosystem service such as *control of erosion rates*, the simple percentage does not indicate whether the change is positive or negative for the provision of the service. The analysis of the causes of change is important to understand the relationships between ecosystem condition and ecosystem services. Nevertheless, such an analysis was beyond the scope of this study.

*Climate indicators* were calculated based on climate data for Germany for at least 30 years whenever they were available. There are, however, some uncertainties with these data, and also other data used in the study. These uncertainties are due to the different data collection methods and to interpolations and missing or erroneous observations. Also, for the climatic variables, the measurement network has changed over time and this affects the comparison of grid fields for the different years [46]. Another possible limitation of the *climate indicators* is that the MAES framework does not provide guidelines about which specific parameters should be used to assess climate change. We selected the most relevant indicators based on the possible influence on the ecosystem service *control of erosion rates*. This selection may seem arbitrary when looking at the general condition of the ecosystem or when making comparisons with other ecosystem services other than control of soil erosion. However, it is a valid approach to generate data on soil erosion rates on the regional scale of this case study.

*Soil erosion* was calculated for arable land only, without taking grasslands and forests into account. Additionally, this calculation was made only for water erosion, without including wind erosion, which is a major problem in the northern part of Lower Saxony. Therefore, the actual soil erosion might be higher than our results. Moreover, the calculation of soil erosion was made using the USLE equation that has some limitations, including the underestimation of the impact in thalwegs and gully erosion [45]. Furthermore, we used agricultural statistics and common assumptions about the effects of management practices and conservation measures for the estimation of soil erosion [49]. This approach is less precise and less explicit than the use of detailed monitoring data [25].

*Loss of organic matter* values were obtained from the ESDAC, which was considered a useful and sufficient approach for our study area. However, these values were originally calculated for the continental scale of Europe. This means that some uncertainties exist regarding the accuracy of the results for the regional level. Although the application of the CENTURY model at smaller scales is technically feasible, there is still a lack of input data [51]. The collection and processing of these data would help to improve the accuracy of the results. However, this is very time-consuming and also out of the scope of our study.

#### 4.1.3 Ecosystem condition indicators

*Crop diversity* was estimated based on the number of crop species in a diameter of 10 km (see S1 File). Although this calculation differs from the one suggested in the MAES framework (N° of crops/10 km × 10 km), it is also an approximation that reflects crop diversity. However, similar to the indicator *change in ecosystem extent*, this indicator does not provide sufficient information to determine whether this number is favourable or not when comparing it with the ecosystem service *control of erosion rates*. For this, an indicator such as the types of crops in a specific area would be more useful, since some cultures are more prone to soil erosion than others. However, we did not use it in this study, since we aimed to test the feasibility of the proposed indicators.

The *density of semi-natural areas* was calculated in regard to the area of each municipality and not specifically in regard to the area of the agricultural land. This could overshadow the results since we did not determine the exact location of these elements, but instead estimated their proportion within the municipalities. Additionally, the lack of suitable metrics prevented us from calculating the shares of some semi-natural elements such as semi-natural grasslands, hedgerows and buffer strips that could have provided a different picture when it comes to identifying the role of semi-natural vegetation in the provision of ecosystem services such as *control of erosion rates*.

The indicators *share of fallow land*, *share of arable land and share of permanent crops in utilized agricultural areas* as well as *livestock density* were calculated based on official statistical data at the level of the municipalities. However, important factors such as the duration and the management of the fallow land can show different results regarding soil erosion in comparison to cultivated lands [68]. Nonetheless, the results could have been more precise and comparable with other indicators, if spatially explicit data were available for the study area. Furthermore, the bare number of livestock units per hectare does not provide sufficient information about the possible impact of livestock on the provision of ecosystem services, because the different types of livestock as well as their management (e.g. landless production systems vs. grassland-based) are not assessed by this indicator.

*Soil Organic Carbon* was calculated based on spatially explicit data on humus content in the topsoil and then using common conversion factors to obtain the concentration of soil organic carbon. These spatially explicit results were upscaled to the level of the municipalities to allow for the comparison between indicators. However, as with other indicators, this leads to uncertainties in the results, since the soil characteristics, in this case soil organic carbon, are not homogeneously distributed within the area of the municipalities.

*Soil erodibility* (K factor of the USLE) showed the same limitations as the calculated soil erosion described before. Furthermore, upscaling these values to the level of the municipalities can increase uncertainty. Taking into consideration that the contents of silt, sand, clay, and organic matter, as well as other parameters needed to calculate the K factor, usually vary within short distances, these generalizations can lead to less accurate results.

*Bulk density* values were obtained from the ESDAC, which is a useful and sufficient approach for our study, similar to the indicator loss of organic matter mentioned before. However, bulk density shows the same limitations as the indicator loss of organic carbon as these values were also calculated for a continental scale. The processing of available regional data would improve the accuracy of the results, but this would be a very complicated and time-consuming approach. It is also worth noting the high annual variability of this indicator, which can be another source of uncertainty in this type of study.

#### 4.1.4 Ecosystem service indicators

The indicator *potential soil loss* (erosion risk) was calculated by assuming that the whole arable land is bare soil, taking into account only natural soil erosion by water. The results aggregated to the municipalities provide an estimate of erosion risk and could show a general picture of the ecosystem condition, when combined with other indicators.

As mentioned in Section 3.1.3, low values of *prevented soil loss* also occurred in areas with low *soil erosion risk*, but this does not necessarily mean that the service supply is low. This shows that only calculating the *prevented soil loss* is insufficient to determine the actual ecosystem service supply [25].

The *provision capacity* indicator, which reflects the proportion of the potential soil loss that is mitigated by the ecosystem service *control of erosion rates*, allows us to identify the service supply and to possibly assess different management practices. However, as with other indicators, provision capacity was upscaled to the municipality level and some aspects—e.g. the presence and distance to watercourses, relief characteristics like thalwegs, presence of tramlines and wheel tracks, as well as management measures, which affect the provision capacity [24, 80]—could not be identified and hence the results are less accurate than they would be with spatially explicit data.

### 4.2. Relationships between agroecosystem condition and control of erosion rates

Since the adoption of the EU Biodiversity Strategy to 2020, the mapping and assessment of European ecosystems and their services has increased [81–83]. However, understanding the interdependencies between biodiversity, ecosystem functioning, and ecosystem services is still a major challenge [5, 6]. Although several studies provide evidence of the positive relationships between biodiversity, natural capital, and ecosystem services [2, 84], there is no consensus on what these links are and how they concretely operate [85].

Although we were not able to establish the causalities among the indicators with the correlation analysis, we observed some strong relations. Almost all the environmental pressure indicators are strongly related to the ecosystem service *control of erosion rates*, except for the indicators *change in ecosystem extent*, *mean annual precipitation*, *days with precipitation equal or higher than 10 mm*, and *summer soil moisture*. Regarding the ecosystem condition indicators, we identified that five out of nine indicators are strongly related to control of erosion rates. The indicators *crop diversity*, *share of fallow and arable land*, and *soil organic carbon* do not show a strong correlation. As expected, there is a positive correlation between the ecosystem service indicators *potential soil loss* and *provision capacity*, and the *control of erosion rates*. This analysis should be perceived with caution, due to the limitations mentioned above.

### 4.3. Recommended land management measures to reduce soil erosion

Based on the overlaps presented in Section 3.2.2., we identified municipalities as priority areas in which the risk of soil erosion is medium or high, the provision capacity is low, and the condition levels are low. Our data identified these *problematic* municipalities in the district of Northeim in the southern region of Lower Saxony. For these areas, it is necessary to implement measures to reduce the impact of pressures, improve the ecosystem condition, and soil conservation. Also, analyses on local scale must be carried out and measures must take into account site-specific characteristics such as soil, crop varieties, soil degradation, and farming practices [19]. When looking at the types of crops in Northeim, we identified cereals, grasslands, oilseeds, pastures for extensive grazing, and fodder plants (maize) as the main agricultural land uses. These crops may have effects on soil degradation and erosion problems due to excessive tillage and crop residue removal [45]. Therefore, the implementation of conservation farming is essential to solve these problems because it can reduce soil erosion by ensuring the protection of the soil surface with residue retention and increased water infiltration.

The techniques applied in conservation farming include permanent soil cover with crop residues, which protects the ground surface and provides organic material, thereby improving soil quality. Other methods are the growth of diverse crop species in the same field and crop rotation, especially crops such as legumes and grasses [36]. Conservation farming also involves minimum soil disturbance that has positive effects on biotic soil activity and leads to increased stability of soil aggregates [37]. Another practice that could be applied to guarantee soil conservation in vulnerable areas is agroforestry. This practice integrates trees with animals or crops or both, increasing the fixation of nitrogen and the return of organic matter to the soil, preserving the fertility and structure of the soil [36]. All these measures impact soil condition and hence agroecosystems condition. Therefore, indicators that address these measures should be taken into account when assessing agroecosystems. The indicators proposed by the MAES working group, as they are implemented in this study, are not able to fully address agroecosystem condition relevant for soil protection and soil erosion prevention. Indicators that analyse the effect of crop species on soil erosion, the use of cover crops and other soil conservation measures should be included in the list of proposed indicators.

### 4.4. Potential for MAES/policy implementation

The normalization presented in Section 2.9 and Figs 6 and 7 aimed to facilitate the comparison between indicators and to visualize the relations between ecosystem condition, environmental pressures, erosion risk, and provision capacity. It is worth highlighting that these representations are somewhat arbitrary and other combinations and overlaps of indicators could provide different results. Furthermore, the results presented here come from a methodological study, aiming at testing an existing framework and respective indicators. These results do not yet have the potential to be used for policy decisions or implement the measures described above to improve the condition or reduce the pressures on agroecosystems, at least not without more detailed, and if possible, spatially explicit data. Our maps should provide a general idea of the environmental pressures, ecosystem condition, soil erosion risk, and the *control of erosion rates* provision capacity in Lower Saxony. These maps should also raise awareness for areas where special attention should be paid to avoid or mitigate ecosystem degradation.

Composite indicators can be used to provide insights on environmental condition, as well as sustainability, quality of life, and economy [86]. Such indicators have been useful in policy analysis and public communication because they seem to be straightforward comprehensible for the general public [87]. However, we did not develop a composite indicator for three main reasons: First, it could add an extra ambiguity to the results. Second, the suggested indicators would not be sufficient to build a trustworthy index, since threshold levels that would help to determine the overall condition have not been defined for all the indicators. Third, it was not the aim of the study to come up with a composite indicator.

## 5. Conclusions

This is- to our knowledge—the first study that tests the MAES framework and indicators for the assessment of the condition of agroecosystems in a regional scale case study. Our study also analyses the relationships between ecosystem condition and the provision of a selected ecosystem service, in particular, *control of erosion rates*. This assessment can identify the suitability of these indicators, check the data availability for respective indicator quantification and describe ecosystem condition on a regional scale.

Although we were not able to establish clear causalities among the indicators, our results identified positive, negative, and no significant correlations between the different pressures and condition indicators, and *control of erosion rates* despite their limitations and data availability. The idea behind the MAES framework is to show the general condition of an ecosystem in the context of ecosystem services supply. However, when looking at the relationships between ecosystem condition and ecosystem services, we observed that not all proposed indicators are suitable to explain to what extent agroecosystems can provide specific ecosystem services. Condition indicators on crop management and soil conservation measures, which are directly linked to the ecosystem service *control of erosion rates*, are missing in the list of indicators proposed in the MAES framework. Additionally, if indicators are to be applied in national or regional scale studies, it is important to consider that trend and high-resolution data are not always available in sufficient quality and resolution. These limitations may undermine the results and hence their comparability with other regions.

Future research should also assess other ecosystem services/ecosystem services bundles provided by agroecosystems. These assessments would facilitate the identification of synergies and trade-offs, both between ecosystem services and between ecosystem condition parameters that may have a different degree of influence on ecosystem services. Besides, a more precise definition of reference conditions, although complicated for agroecosystems, is essential to provide more accurate information on the condition of the ecosystem, which should lead to better policy and management decisions.

## Supporting information

S1 File Quantification of indicators. (PDF)

S2 File Indicators per municipality. (XLSX)

S3 File Maps. (PDF)

S1 Table Results of tests per indicator. (XLSX)
