# Supplementary material for: Assessment of the relationships between agroecosystem condition and the ecosystem service soil erosion regulation in Northern Germany
Source: PLoS One. 2020 Dec 7;15(12):e0234288. doi: 10.1371/journal.pone.0234288 (PMC7721136; doi:10.1371/journal.pone.0234288)
Supplement: S1 File — (PDF) [file pone.0234288.s001.pdf]

SUPPORTING INFORMATION

1. Quantification of indicators

1.1. Environmental pressure indicators

Change in ecosystem extent was calculated based on the CORINE Land Cover change layer for the years 2006 and 2012 [1]. We obtained some average values of change in agroecosystem extent, based on three conditions: (i) Decrease in extent: changes from agricultural areas (Class 2 in the CORINE classification) to other land uses. (ii) Increase in extent: changes from other land uses to agricultural areas. And (iii) No change: changes within the class 2, for example from class 211 (non-irrigated arable land) to class 242 (complex cultivation patterns). No change also represents those raster cells that are part of the study area but were not included in the CORINE change layers. We created a 100 m raster and assigned the value of 1 to all the cells where the three different conditions were met. We then calculated the sum of the 100 m raster cell in a 1 km raster to determine the percentage of change in 1 km^2. The values that correspond to decrease in extent were multiplied by -1 and the values of no change were converted into 0. The raster data were combined with the shapes of the municipalities to calculate the percentage of change per municipality.

Climate

The data of the climate indicators were obtained from the German Weather Service (Deutscher Wetterdienst DWD) [2] with a spatial resolution of $1km^2$. The detailed description of the indicators is provided below:

Mean annual temperature: Corresponds to the annual mean of the monthly averaged mean daily air temperature in 2 m height above ground given in degree Celsius, for the period between 1988 and 2018.

Mean annual precipitation: Corresponds to the annual sum of monthly total precipitation given in mm for the period between 1988 and 2018.

Drought index: The data of annual drought index (from de Martonne [3]) dMI was calculated with:

$$dMI = {}^{P}\!/_{(T+10)} \qquad (1)$$

Where T [in degree Celsius] was obtained from temperature grids and P [in mm] from precipitation grids for the period between 1995 and 2018.

Precipitation 10 mm, 20 mm and 30 mm: Corresponds to the number of days with precipitations equal or higher than 10, 20 and 30 mm respectively, averaged for the period between 1988 and 2018.

Beginning of vegetation period: Corresponds to the consecutive days of the year in which the first spring begins averaged for the period between 1992 and 2018.

Summer soil moisture was obtained from the DWD who used the AMBAV model that calculates the evapotranspiration and the soil-water balance for different crops [4]. This was done based on the soil moisture in 60 cm depth under grass derived from selected stations for the period between 1991 and 2010, for June, July and August.

Soil erosion corresponds to the mean actual soil loss and was calculated with the Universal Soil Loss Equation (USLE), applying the German standard DIN 19708 [5].
The loss rates were modelled as raster GIS layers for Lower Saxony in a resolution of 50 m based on the methodology applied by [6]. The USLE was calculated as:

$$Actual\ soil\ loss = K\ \times R\ \times LS\ \times C\ \times P \qquad (2)$$

Where
K is the soil erodibility factor [t h ha^{-1} N^{-1}] for Germany based on the soil overview maps (Bodenkundliche Übersichtskarten [7]) and the approach of Auerswald & Elhaus [8]
R is the rain erosivity factor [N ha^{-1} per year] for Germany based on mean annual summer precipitations for 1980-2010 from the DWD and the regression equation from standard DIN 19708.
LS is the topography factor (length and slope) [dimensionless] based on a 50 m Digital Elevation Model (DEM) and the approach from the DIN 19708.
C is the crop management factor [dimensionless] based on crop rotations from culture portions on the level of the municipality for the year 2010.
P is the erosion control practices factor [dimensionless] considered in this study as 1 due to lack of detailed data for the resolution we used.

The results from the calculations were clipped to the arable land according to the land use type *Non-irrigated arable land (2.1.1)* of the CORINE Land Cover 2012 [9].

Loss of organic matter: This indicator was obtained from the average eroded soil organic carbon dataset calculated for the EU [10]. This was done by applying the CENTURY model, which simulates the carbon and nitrogen dynamics in cultivated or natural systems, coupled with soil

erosion [11]. We calculated the average soil organic carbon loss per municipality based on a 1 km raster data set.

1.2. Ecosystem condition indicators

Crop diversity was calculated based on the number of field blocks in the year 2018. These data were obtained from the Land Development and Agricultural Support programme of Lower Saxony (LEA) Portal [12] of the Lower Saxonian Ministry of Food, Agriculture, and Consumer Protection. We calculated this indicator by estimating the number of crops in a regular 1km raster by applying a moving window analysis with a radius of 5 km. By counting the overlaying field, we added them up and obtained the diversity of crops in a diameter of 10 km around the midpoints of each raster cell. The raster data were combined with the shapes of the municipalities to calculate the average number of crops per municipality.

Density of semi-natural areas was calculated based on the CORINE Land Cover data of 2012. The land cover classes were selected according to those proposed by García-Feced et al [13]: 2.3.1. Pastures, 2.4.3. Land principally occupied by agriculture, with significant areas of natural vegetation and natural grasslands.The land cover class 2.4.4. Agro-forestry areas is not present in the dataset in Lower Saxony. The data of these land cover classes were combined with the shapes of the municipalities to calculate the percentage of semi-natural areas in relation to the area of the municipality.

The *share of fallow land in Utilized Agricultural Area (UAA)* was calculated by dividing the number of hectares of fallow land and set aside or decommissioning land over the number of hectares of UAA per municipality. The same approach was used for the calculation of the share of arable land and permanent crops in the UAA. The data for the estimation of these indicators were obtained from the Thünen-Atlas (Collection of agricultural data from Germany) [14,15] for the year 2010.

Livestock density was calculated by dividing the number of livestock units over the number of hectares of UAA per municipality, both were obtained from the Thünen-Atlas [14,15] for the year 2010.

Soil Organic Carbon was calculated based on the datasets on *humus content in the topsoil* and the *usage-differentiated soil survey map* obtained from the Federal Institute for Geosciences and Natural Resources (BGR for its acronym in German) [16]. We used depths from 0 to 10 cm below the soil surface for grassland, pasture and forestry and depths from 0 to 30 cm under the soil surface for crop lands. To convert the humus content data into soil organic carbon data, we used the following equations:

$Humus = C_{org} \times 1.72$ (3) For mineral soil (van Bemmelen factor) [17]

$Humus = C_{org} \times 2$ (4) For peat

Soil erodibility was calculated as the K factor explained before in the USLE equation [6].

Bulk density was obtained from the bulk density calculated for the EU [18]. This physical property was derived from soil texture data sets as described by Ballabio et al [19].

1.3. Ecosystem service indicators

The ecosystem service *control of erosion rates* is a regulating ecosystem service that mitigates the structural impact *potential soil loss*. In this study, we adapted the conceptual framework for assessing the provision of regulating ecosystem services developed by Guerra et al [20]. Fig S1 is a graphical representation of the adapted framework, where A shows the concept for the assessment of the ecosystem service *control of erosion rates* and B shows the implementation in this study.

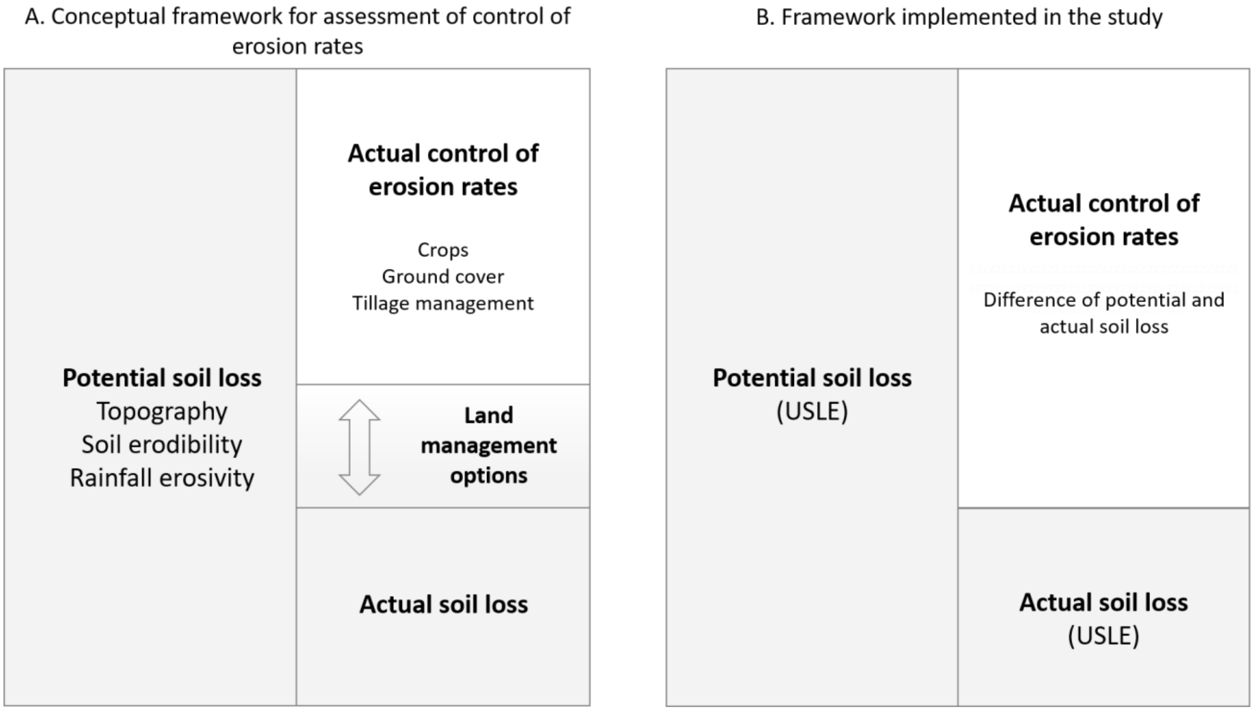

Fig S1. Conceptual framework applied for the assessment of the ecosystem service *control of erosion rates* based on Guerra et al., 2014 [20].

Soil erosion risk is the mean potential soil loss defined as the amount of soil that is lost when there is no vegetation covering the ground [21]. Potential soil loss is determined by the USLE

factors rain erosivity (R), soil erodibility (K) and topography (LS) as shown in the following equation [6] (also described before for the pressure indicator *soil erosion*):

$$Potential\ soil\ loss\ = R\ \times K\ \times LS \qquad (5)$$

The results from the calculations were clipped to the arable land according to the land use type *Non-irrigated arable land (2.1.1)* of the CORINE Land Cover 2012.

The *actual control of erosion rates* denotes the prevented soil erosion calculated as the difference of *potential soil loss* (soil erosion risk) and *actual soil loss* (soil erosion - described before).

Another indicator used to calculate the ecosystem service *control of erosion rates* is the *provision capacity*. This is defined as the fraction of the potential soil loss that was mitigated by the actual service provision. It ranges from 0 to 1, where 0 represents no mitigation and 1 complete mitigation and was calculated based on the USLE model results for Lower Saxony (see [21] for more detailed explanation of the approach).

The data of these indicators were combined with the shapes of the municipalities to calculate the average value per municipality.

References

1. European Environment Agency. Land Cover Change (LCC) 2006-2012 [Internet]. 2019. Available from: https://land.copernicus.eu/pan-european/corine-land-cover/lcc-2006-2012?tab=metadata
2. Deutscher Wetterdienst. CDC- Climate Data Center [Internet]. 2018. Available from: https://cdc.dwd.de/portal/
3. Martonne DE. Traite de Geographie Physique: 3 tomes. Paris; 1925.
4. Löpmeier F. Berechnung der Bodenfeuchte und Verdunstung mittels agrarmeteorologischer Modelle. Zeitschrift für Bewaesserungswirtschaft. 1994;(29):157–67.
5. Deutsche Institut für Normung. DIN 19708 Bodenbeschaffenheit - Ermittlung der Erosionsgefährdung von Böden durch Wasser mit Hilfe der ABAG. 2017.
6. Saggau P, Bug J, Gocht A, Krure K. Aktuelle Bodenerosionsgefährdung durch Wind und Wasser in Deutschland. Bodenschutz [Internet]. 2017;4(17):120–5. Available from: https://doi.org/10.37307/j.1868-7741.2017.04.04
7. LBEG. Maps and data [Internet]. 2019 [cited 2019 Mar 15]. Available from: https://www.lbeg.niedersachsen.de/karten_daten_publikationen/karten_daten/
8. Auerswald K, Elhaus D. Ableitung der Bodenerodierbarkeit K anhand der Bodenart. Bodenschutz [Internet]. 2013;4(13):109–13. Available from: https://www.researchgate.net/profile/Karl_Auerswald/publication/259331462_Ableitung_der_Bodenerodierbarkeit_K_anhand_der_Bodenart/links/565f1f9a08aefe619b284d96.pdf
9. European Environment Agency. CORINE Land Cover (CLC) [Internet]. 2012. Available

from: https://land.copernicus.eu/pan-european/corine-land-cover
10. ESDAC. Pan-European SOC stock of agricultural soils [Internet]. 2014 [cited 2018 Oct 10]. Available from: https://esdac.jrc.ec.europa.eu/content/pan-european-soc-stock-agricultural-soils
11. Lugato E, Paustian K, Panagos P, Jones A, Borrelli P. Quantifying the erosion effect on current carbon budget of European agricultural soils at high spatial resolution. Glob Chang Biol [Internet]. 2016;22(5):1976–84. Available from: https://onlinelibrary.wiley.com/doi/full/10.1111/gcb.13198
12. SLA - LEA. Landentwicklung und Agrarförderung Niedersachsen LEA - Portal [Internet]. 2019 [cited 2019 Apr 3]. Available from: https://sla.niedersachsen.de/landentwicklung/LEA/
13. García-Feced C, Weissteiner CJ, Baraldi A, Paracchini ML, Maes J, Zulian G, et al. Semi-natural vegetation in agricultural land: European map and links to ecosystem service supply. Agron Sustain Dev [Internet]. 2014;35(1):273–83. Available from: https://link.springer.com/content/pdf/10.1007/s13593-014-0238-1.pdf
14. Gocht A, Röder N, Meyer-Borstel H. Thünen-Atlas: Landwirtschaftliche Nutzung (1999-2010) [Internet]. Braunschweig; 2014. Available from: https://gdi.thuenen.de/lr/agraratlas
15. Thünen Institute. Thünen Atlas [Internet]. 2019. Available from: https://www.thuenen.de/de/infrastruktur/thuenen-atlas-und-geoinformation/thuenen-atlas/
16. BGR. Federal Institute for Geosciences and Natural Resources [Internet]. 2019. Available from: https://www.bgr.bund.de/EN/Home/homepage_node_en.html
17. Pribyl DW. A critical review of the conventional SOC to SOM conversion factor. Geoderma [Internet]. 2010;156(3–4):75–83. Available from: http://dx.doi.org/10.1016/j.geoderma.2010.02.003
18. ESDAC. Topsoil physical properties for Europe (based on LUCAS topsoil data) [Internet]. 2016 [cited 2019 Jul 17]. Available from: https://esdac.jrc.ec.europa.eu/content/topsoil-physical-properties-europe-based-lucas-topsoil-data
19. Ballabio C, Panagos P, Monatanarella L. Mapping topsoil physical properties at European scale using the LUCAS database. Geoderma [Internet]. 2016;261:110–23. Available from: http://dx.doi.org/10.1016/j.geoderma.2015.07.006
20. Guerra C, Pinto-Correia T, Metzger MJ. Mapping Soil Erosion Prevention Using an Ecosystem Service Modeling Framework for Integrated Land Management and Policy. Ecosystems [Internet]. 2014;17(5):878–89. Available from: https://link.springer.com/article/10.1007/s10021-014-9766-4
21. Steinhoff-Knopp B, Burkhard B. Mapping Control of Erosion Rates: Comparing Model and Monitoring Data for Croplands in Northern Germany. One Ecosyst [Internet]. 2018;3:e26382. Available from: https://oneecosystem.pensoft.net/articles.php?id=26382
