# Supplementary material for: Assessment of the relationships between agroecosystem condition and the ecosystem service soil erosion regulation in Northern Germany
Source: PLoS One. 2020 Dec 7;15(12):e0234288. doi: 10.1371/journal.pone.0234288 (PMC7721136; doi:10.1371/journal.pone.0234288)
Supplement: S3 File — (PDF) [file pone.0234288.s003.pdf]

Supporting information S3 - Maps.

a. Change in ecosystem extent

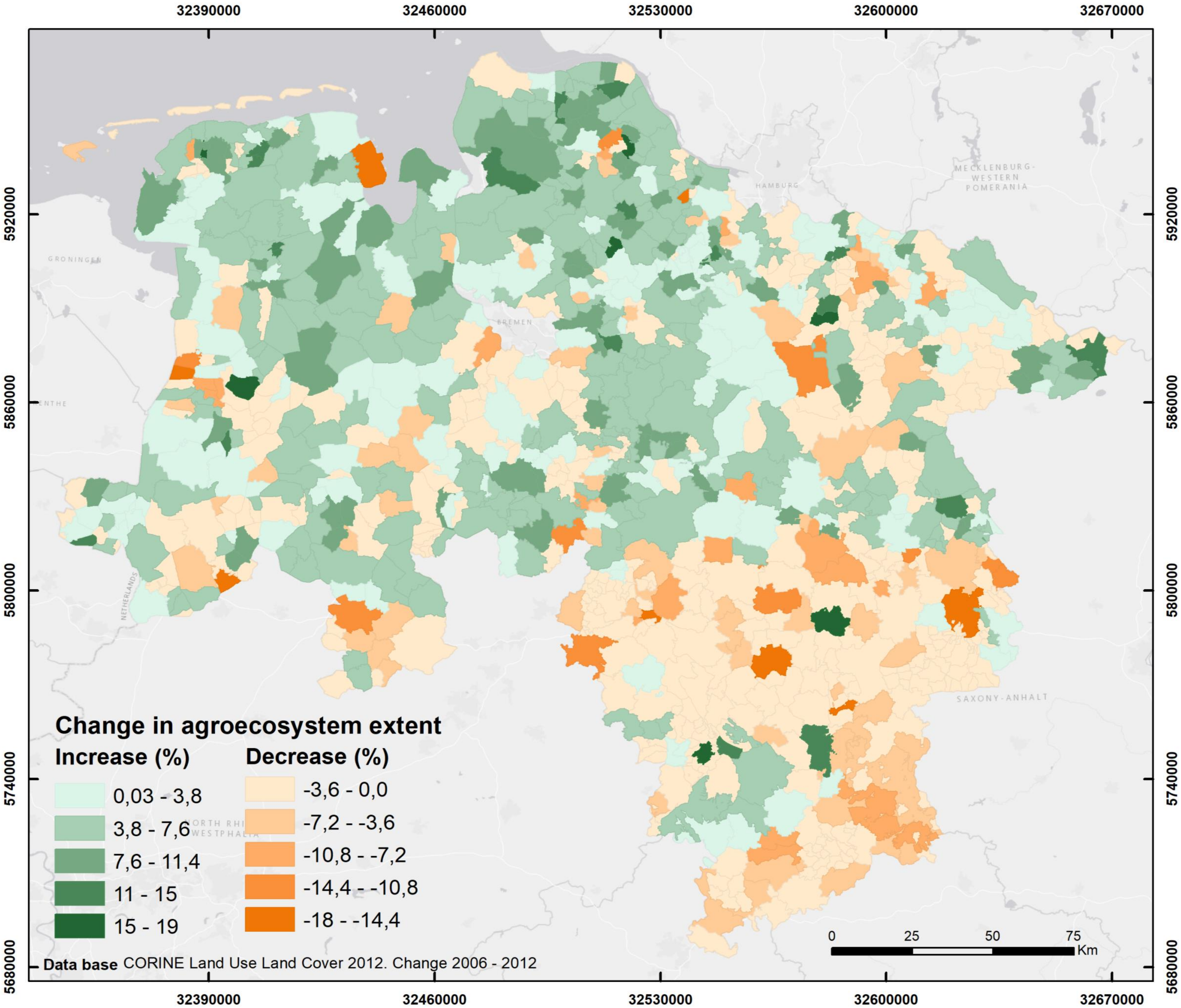

Based on CORINE Land Use Land Cover data of 2012 obtained from the European Environmental Agency [1] and administrative units from the German Federal Agency for Cartography and Geodesy © GeoBasis-DE / BKG (2017) [2].

b. Mean annual temperature

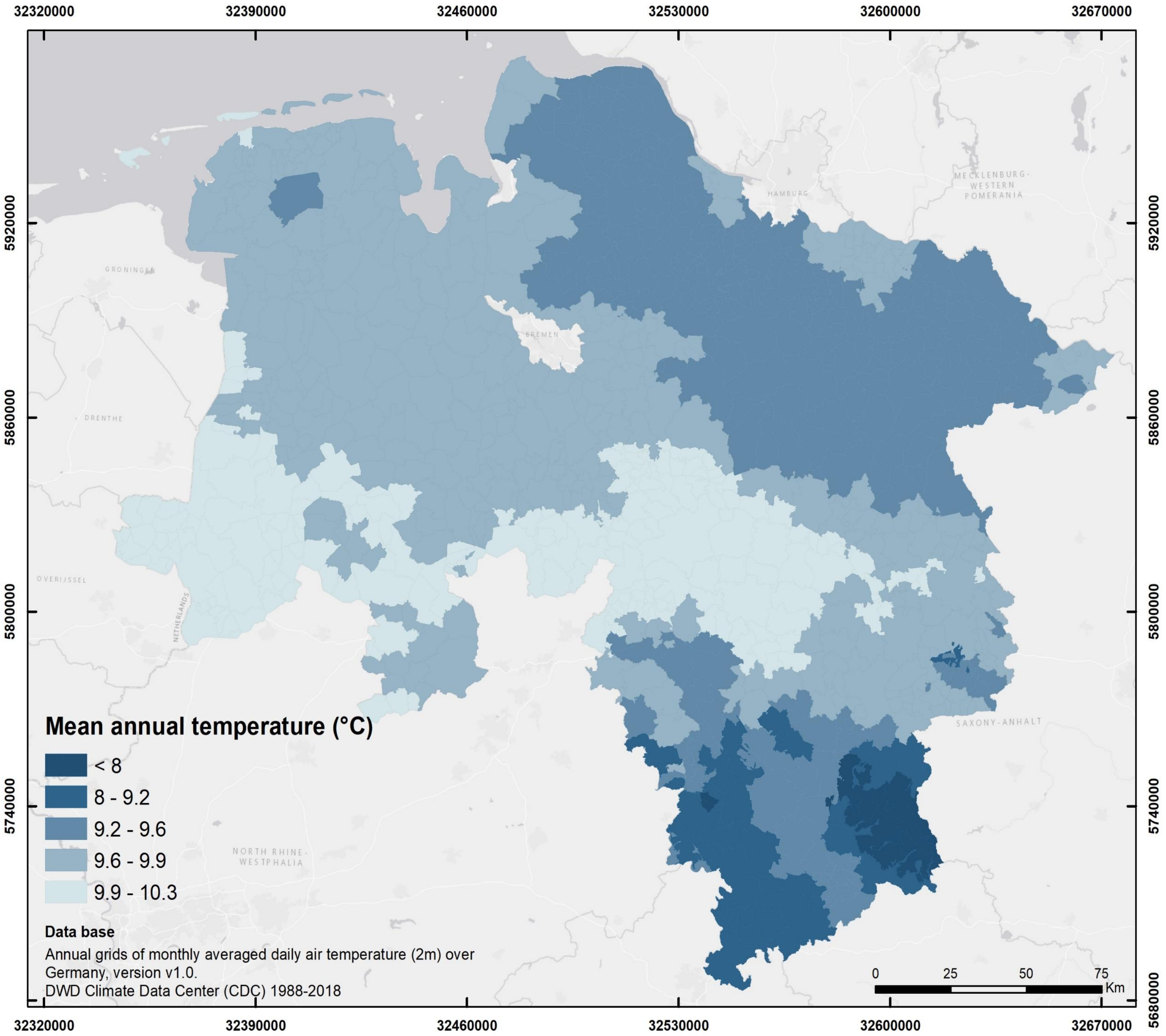

Based on data from the Deutsher Wetterdienst Climate Data Center (CDC OpenData) [3] and the administrative units from the German Federal Agency for Cartography and Geodesy © GeoBasis-DE / BKG (2017) [2].

c. Mean annual precipitation

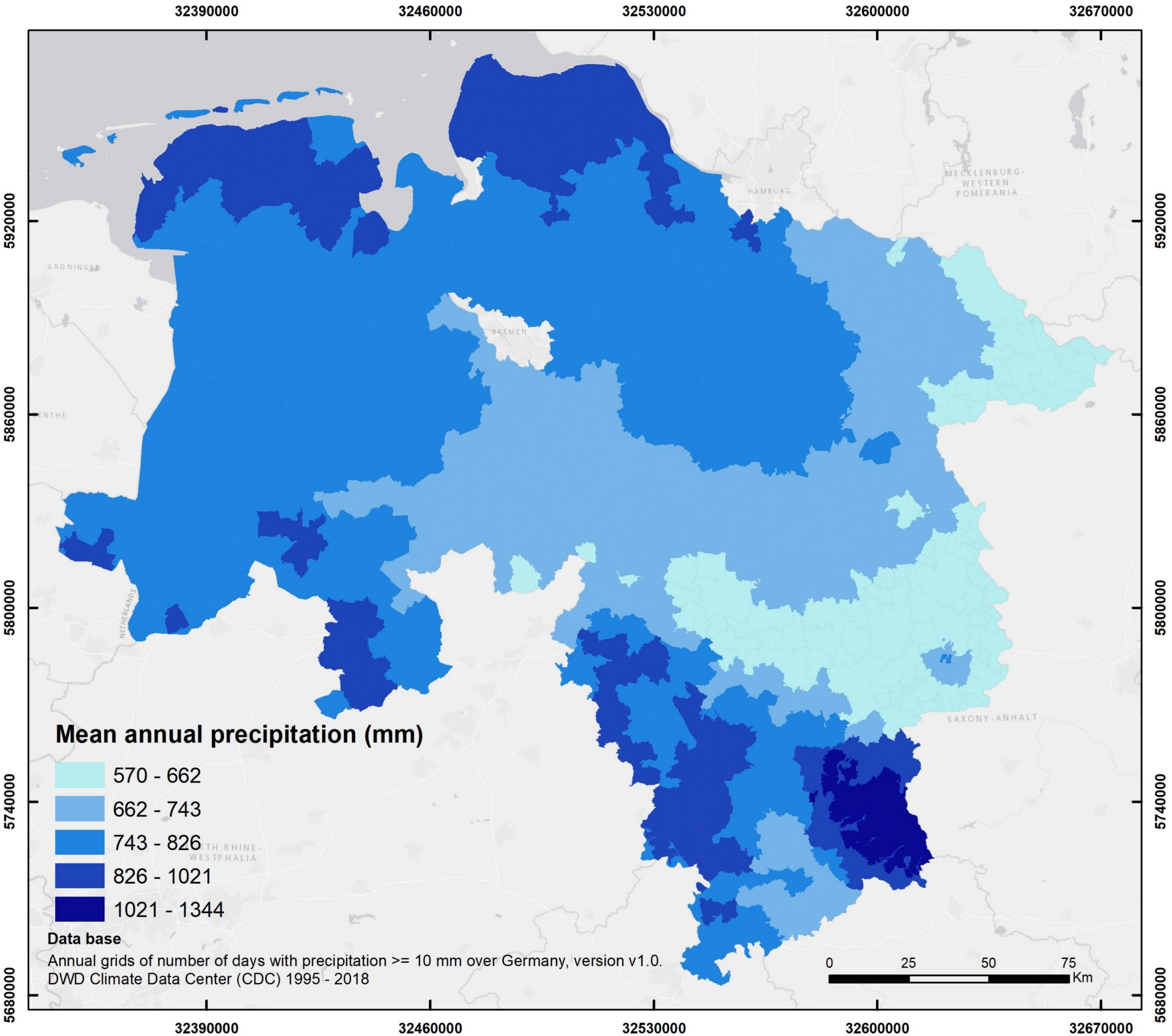

Based on data from the Deutsher Wetterdienst Climate Data Center (CDC OpenData) [3] and the administrative units from the German Federal Agency for Cartography and Geodesy © GeoBasis-DE / BKG (2017) [2].

d. Drought index or aridity index of the Martonne

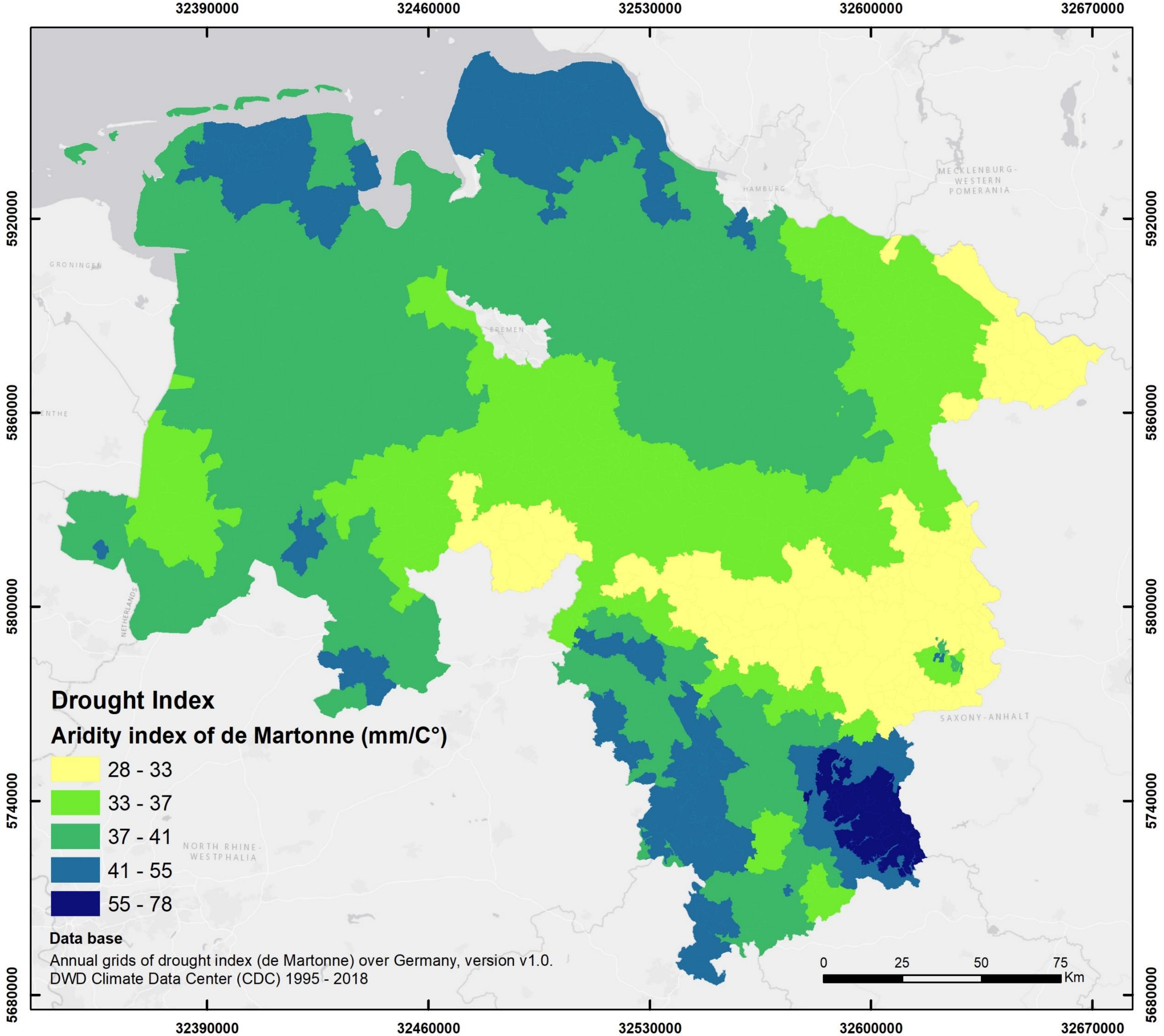

Based on data from the Deutsher Wetterdienst Climate Data Center (CDC OpenData) [3] and the administrative units from the German Federal Agency for Cartography and Geodesy © GeoBasis-DE / BKG (2017) [2].

e. Precipitation 10 mm

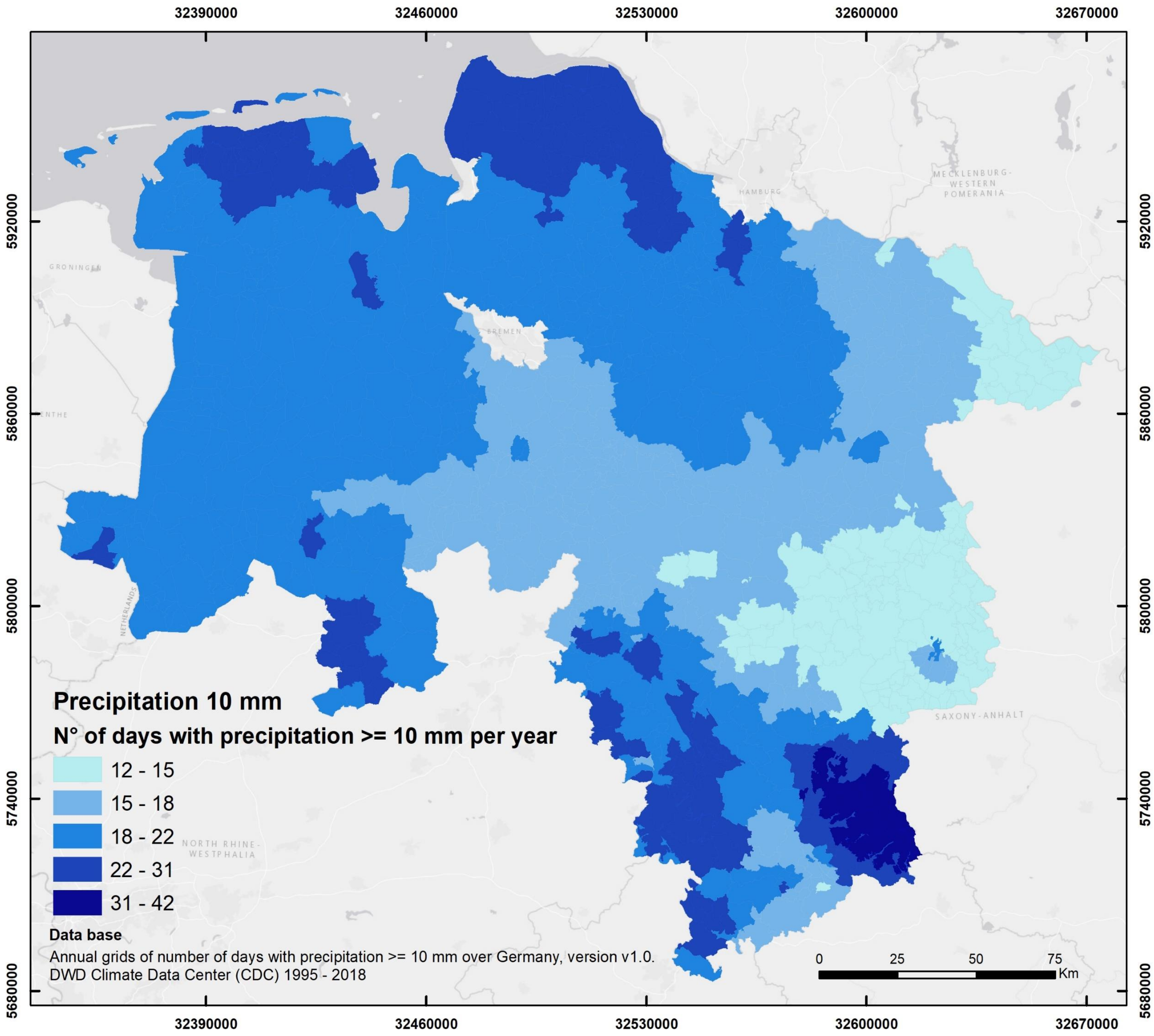

Based on data from the Deutsher Wetterdienst Climate Data Center (CDC OpenData) [3] and the administrative units from the German Federal Agency for Cartography and Geodesy © GeoBasis-DE / BKG (2017) [2].

f. Precipitation 20 mm

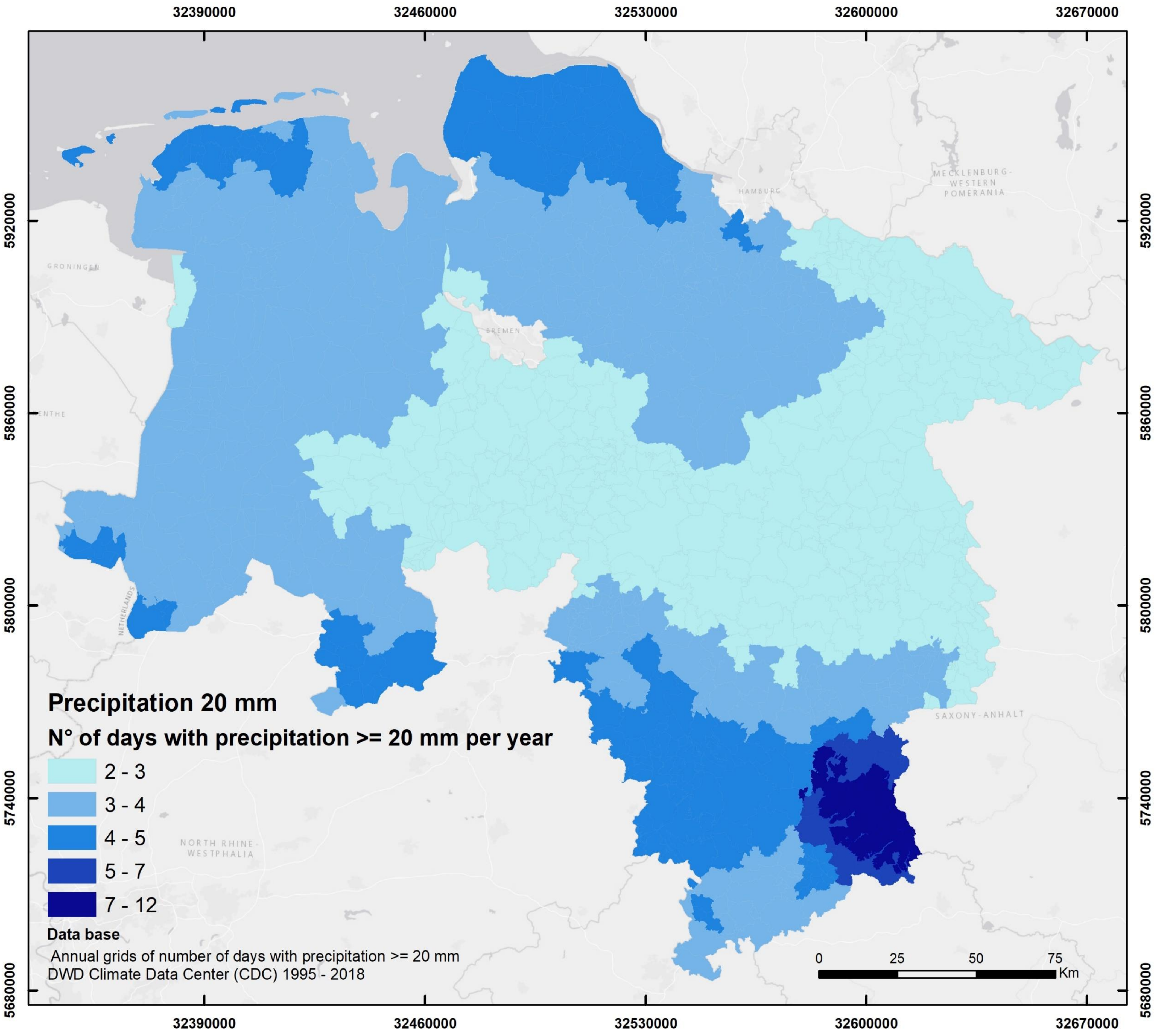

Based on data from the Deutsher Wetterdienst Climate Data Center (CDC OpenData) [3] and the administrative units from the German Federal Agency for Cartography and Geodesy © GeoBasis-DE / BKG (2017) [2].

g. Precipitation 30 mm

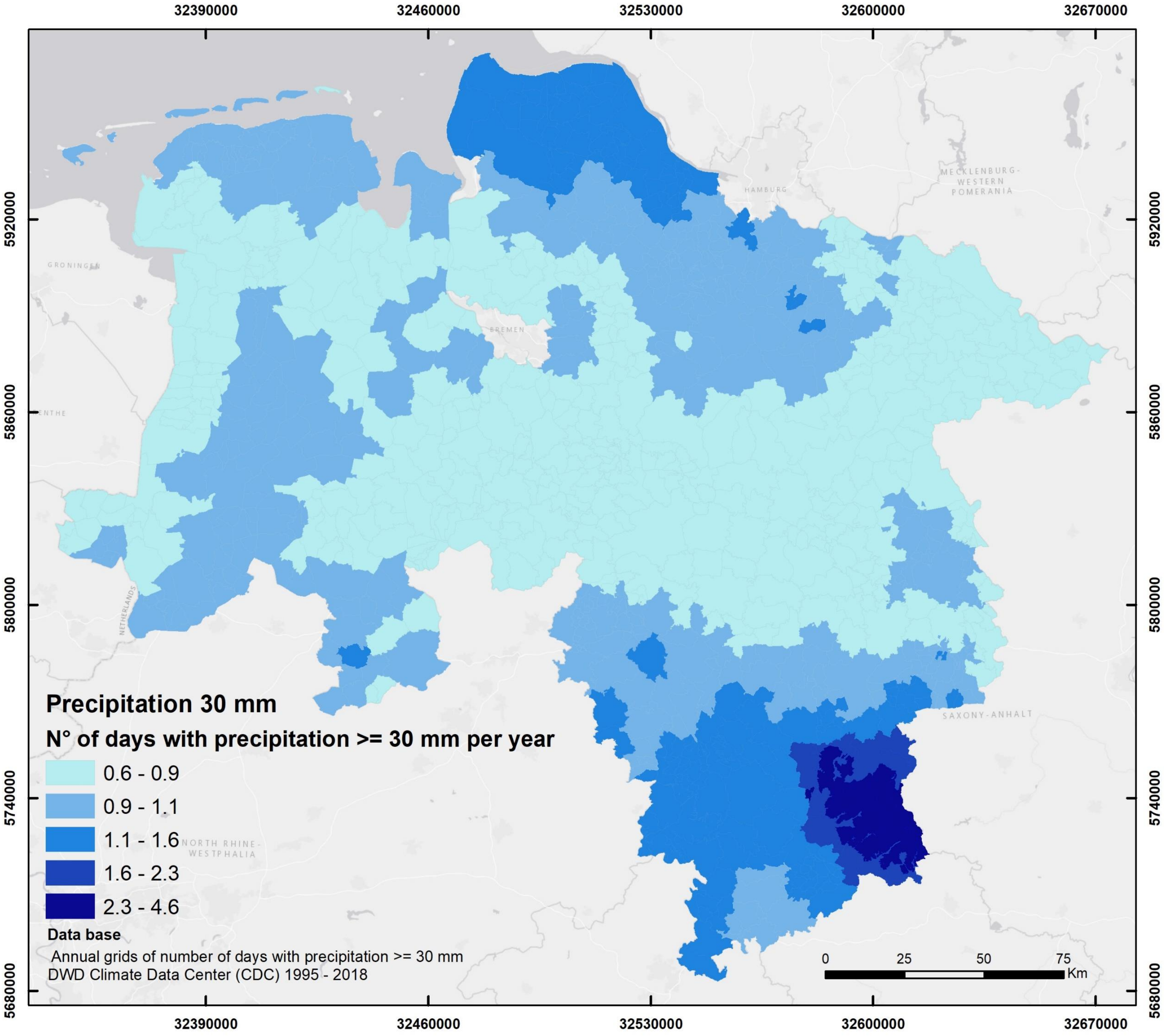

Based on data from the Deutsher Wetterdienst Climate Data Center (CDC OpenData) [3] and the administrative units from the German Federal Agency for Cartography and Geodesy © GeoBasis-DE / BKG (2017) [2].

h. Beginning of vegetation period

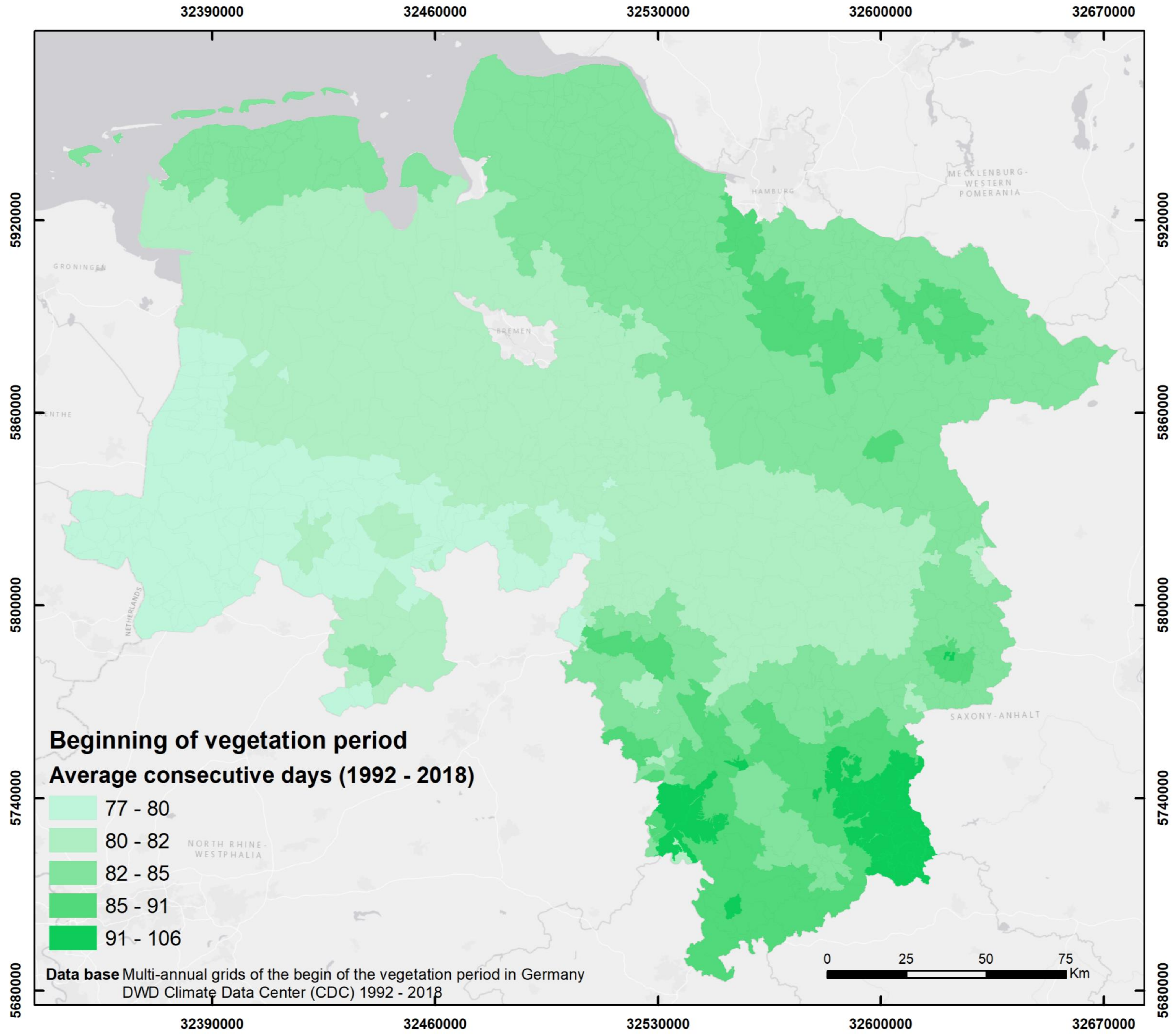

Based on data from the Deutsher Wetterdienst Climate Data Center (CDC OpenData) [3] and the administrative units from the German Federal Agency for Cartography and Geodesy © GeoBasis-DE / BKG (2017) [2].

i. Summer soil moisture

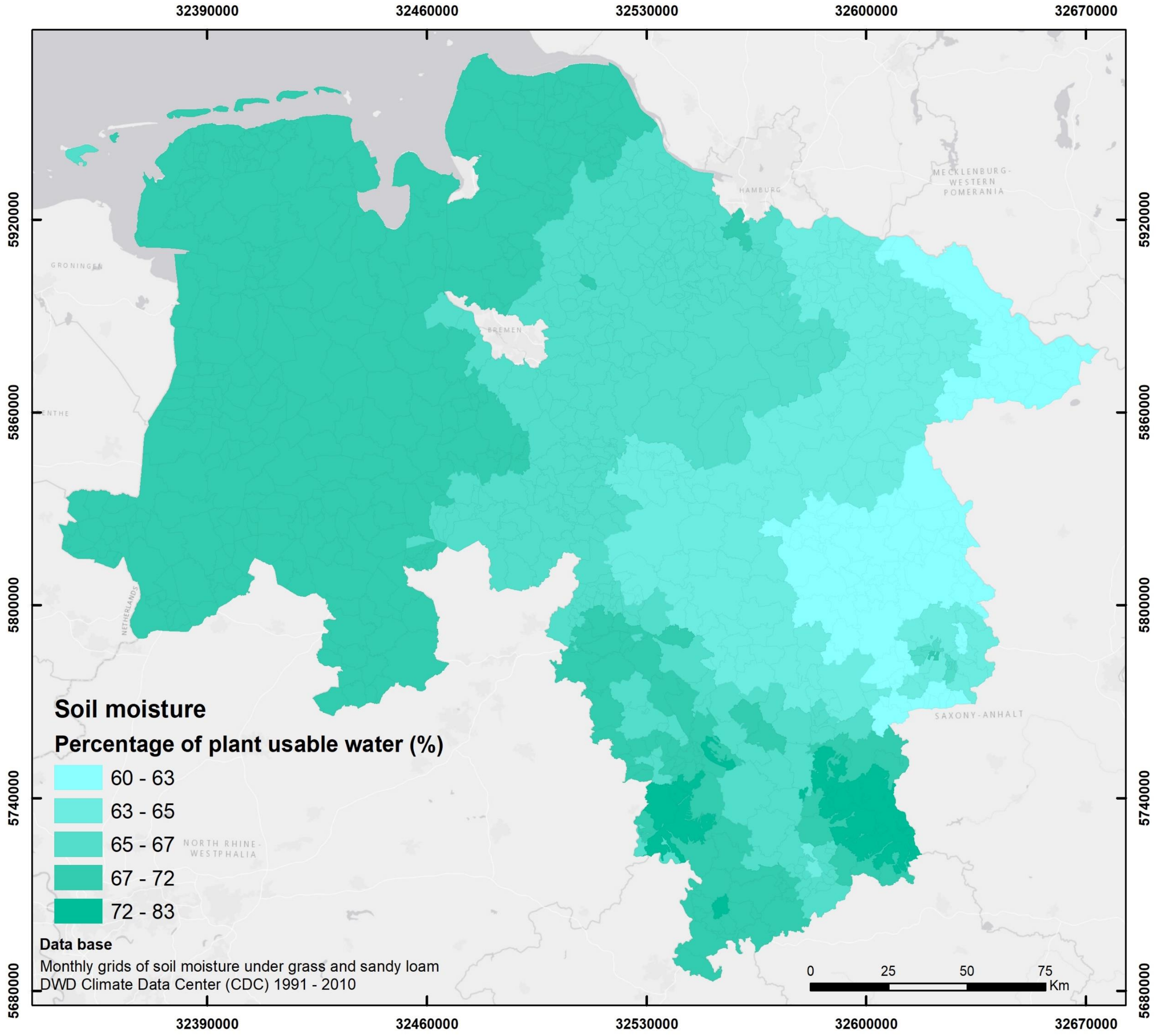

Based on data from the Deutsher Wetterdienst Climate Data Center (CDC OpenData) [3] and the administrative units from the German Federal Agency for Cartography and Geodesy © GeoBasis-DE / BKG (2017) [2].

j. Soil erosion

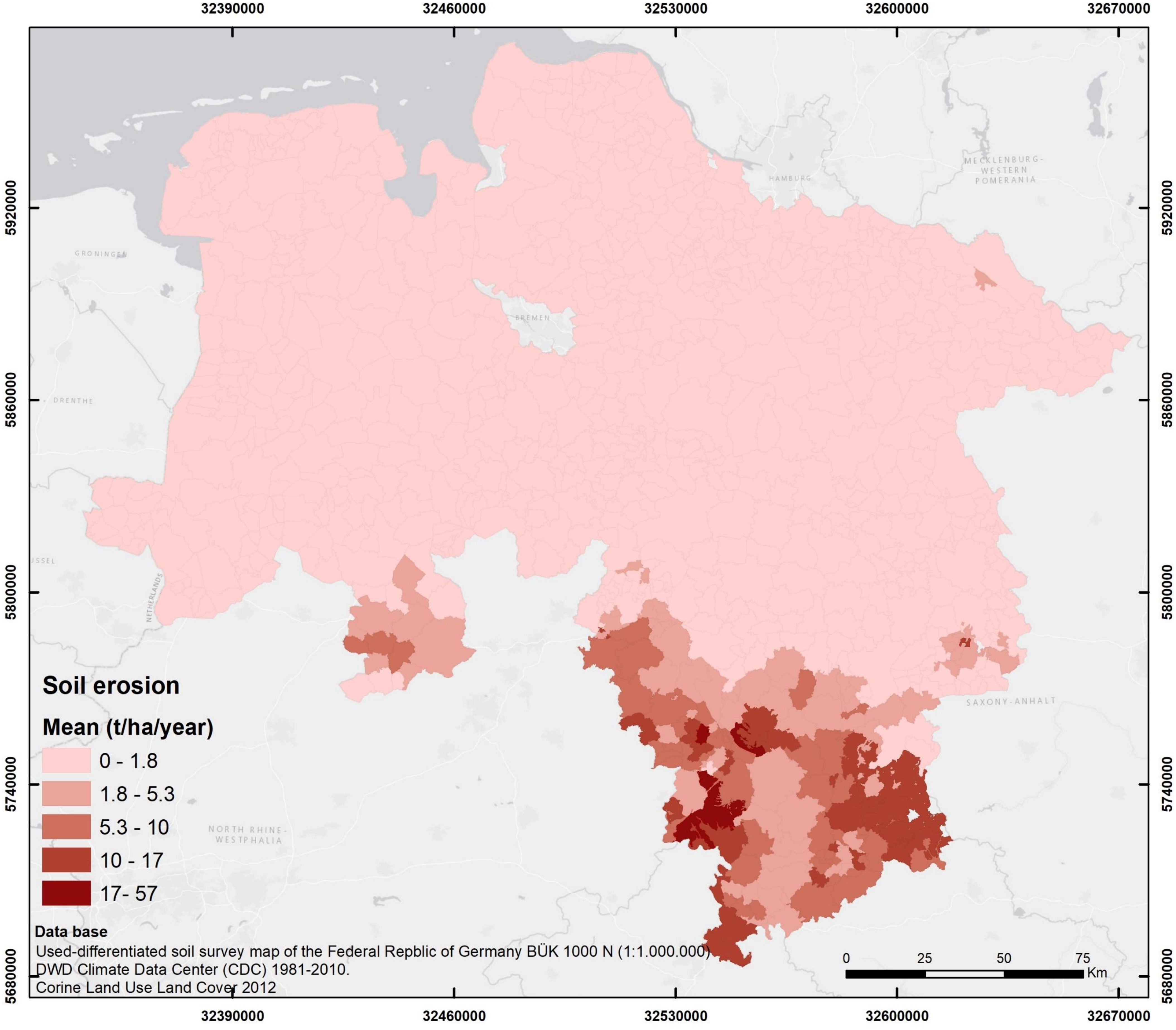

Republished from [4] under a CC BY license, with permission from the German Federal Institute of Geosciences and Natural Resources BÜK1000 V2.1, © BGR, original copyright 2013. Based on the Deutscher Wetterdienst Climate Data Center (CDC OpenData) [3] and the administrative units from the German Federal Agency for Cartography and Geodesy © GeoBasis-DE / BKG (2017) [2].

k. Loss of organic matter

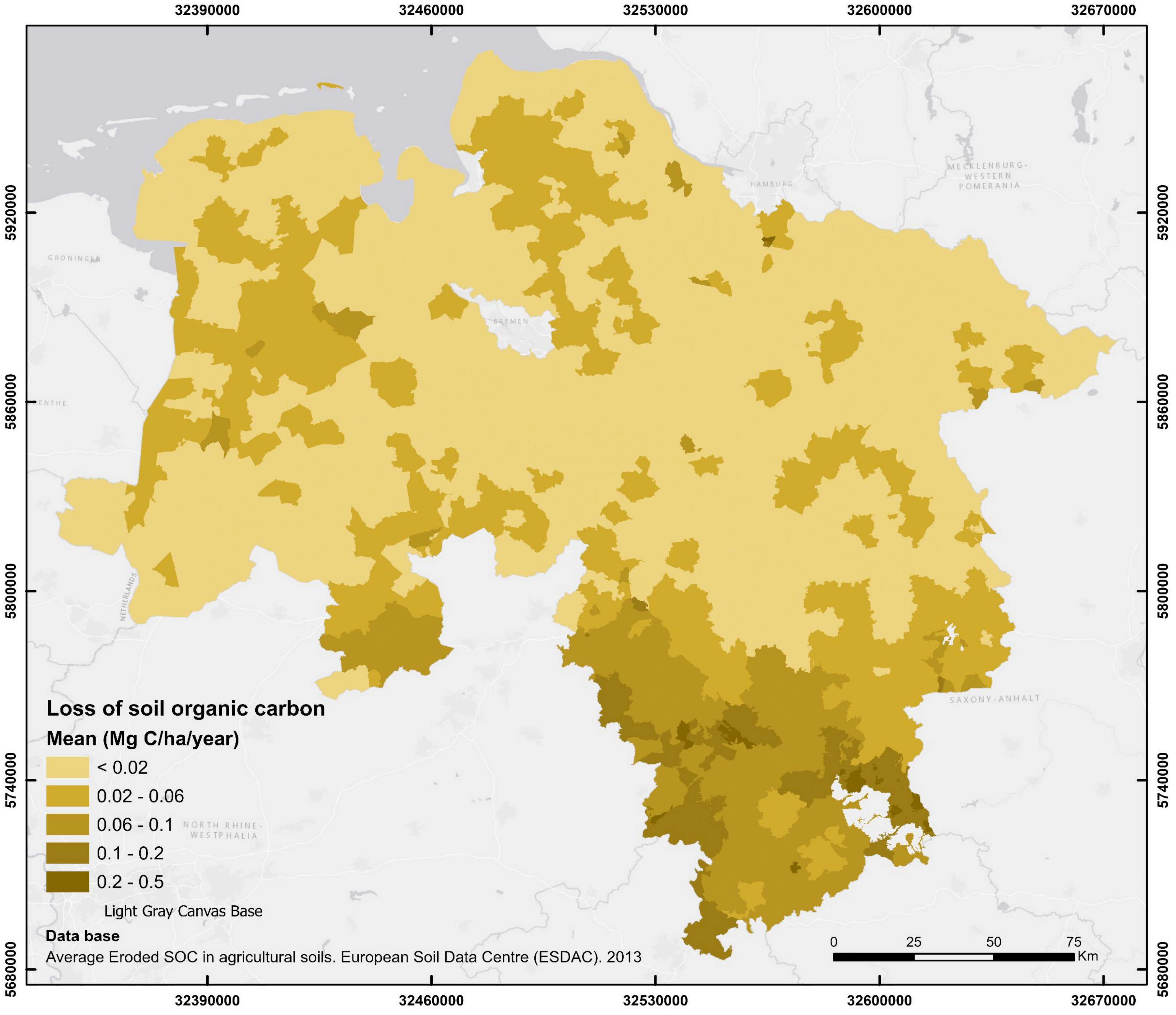

Republished from [5] under a CC BY license, with permission from the European Soil Data Centre (ESDAC), original copyright 2013. Based on the administrative units from the German Federal Agency for Cartography and Geodesy © GeoBasis-DE / BKG (2017) [2].

l. Crop diversity

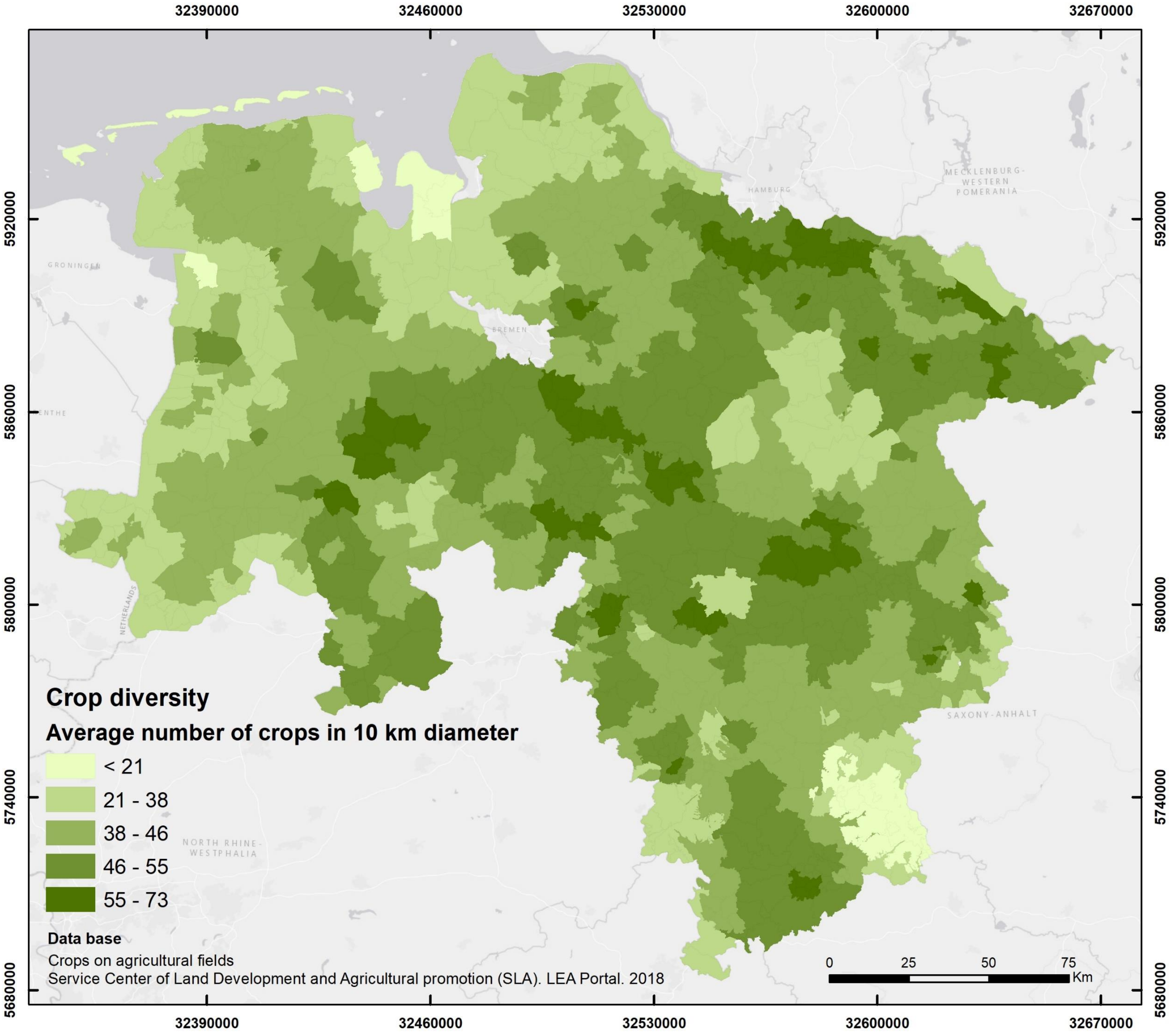

Republished from [6] under a CC BY license, with permission from the Service Center for Land Development and Agricultural Promotion (SLA), original copyright 2018. Based on the administrative units from the German Federal Agency for Cartography and Geodesy. © GeoBasis-DE / BKG (2017) [2].

m. Density of semi-natural elements

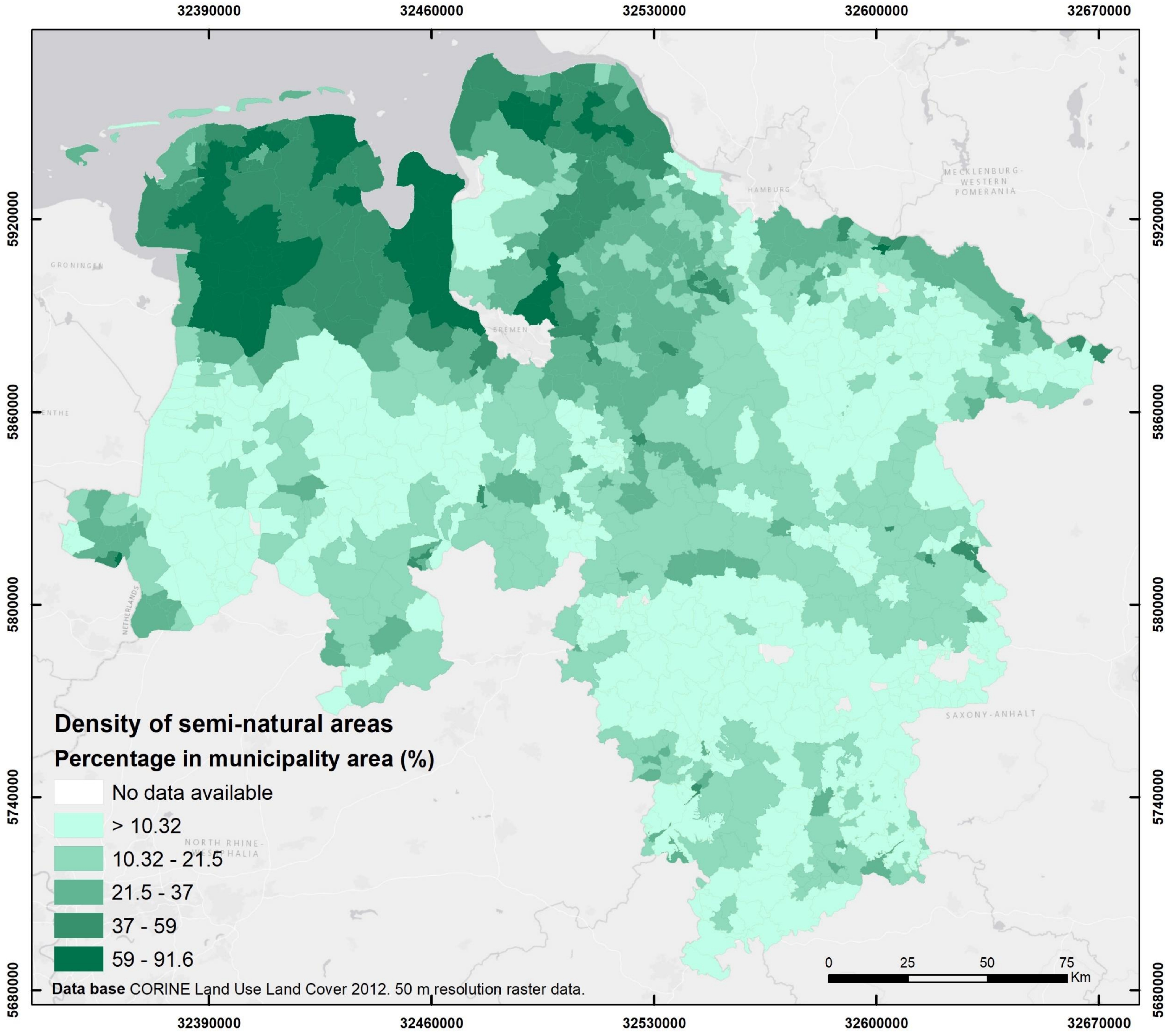

Based on CORINE Land Use Land Cover data, of 2012 obtained from the European Environmental Agency [1] and administrative units from the German Federal Agency for Cartography and Geodesy © GeoBasis-DE / BKG (2017) [2].

n. Share of fallow land in UAA

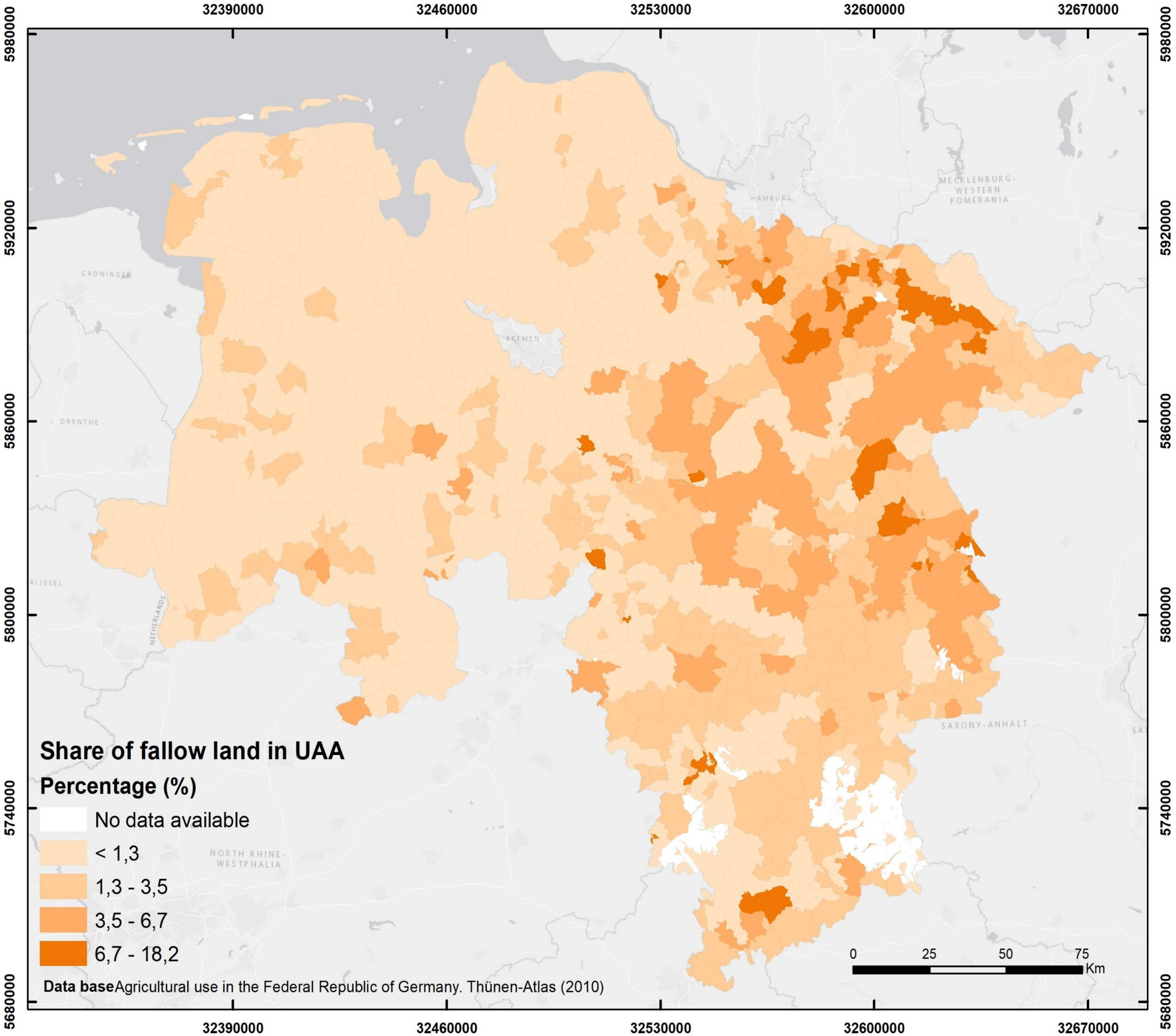

Republished from [7] under a CC BY license, with permission from the Thünen-Atlas (Collection of agricultural data from Germany), original copyright 2010. Based on the administrative units from the German Federal Agency for Cartography and Geodesy © GeoBasis-DE / BKG (2017) [2].

o. Share of arable land in UAA

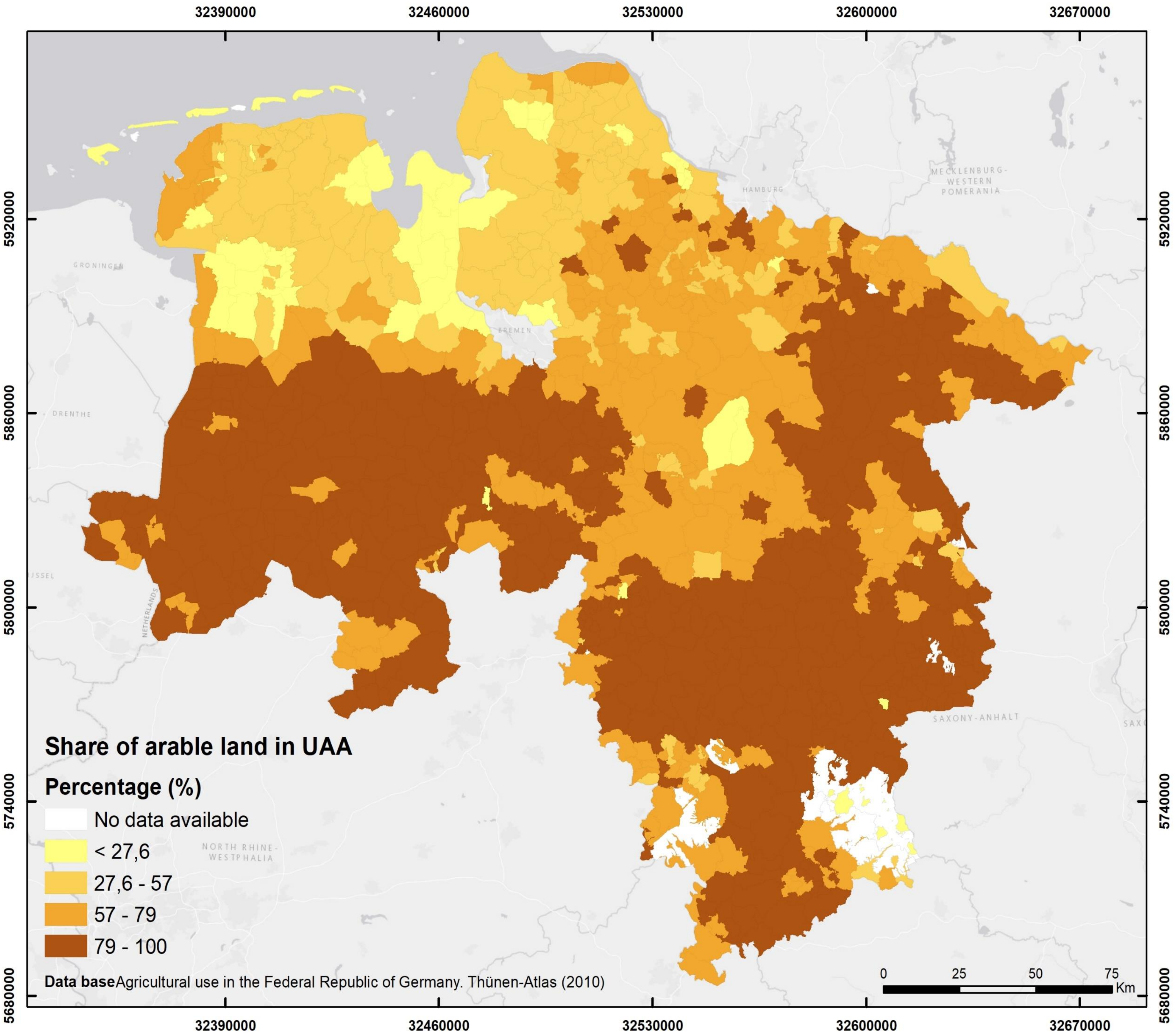

Republished from [7] under a CC BY license, with permission from the Thünen-Atlas (Collection of agricultural data from Germany), original copyright 2010. Based on the administrative units from the German Federal Agency for Cartography and Geodesy © GeoBasis-DE / BKG (2017) [2].

p. Share of permanent crops in UAA

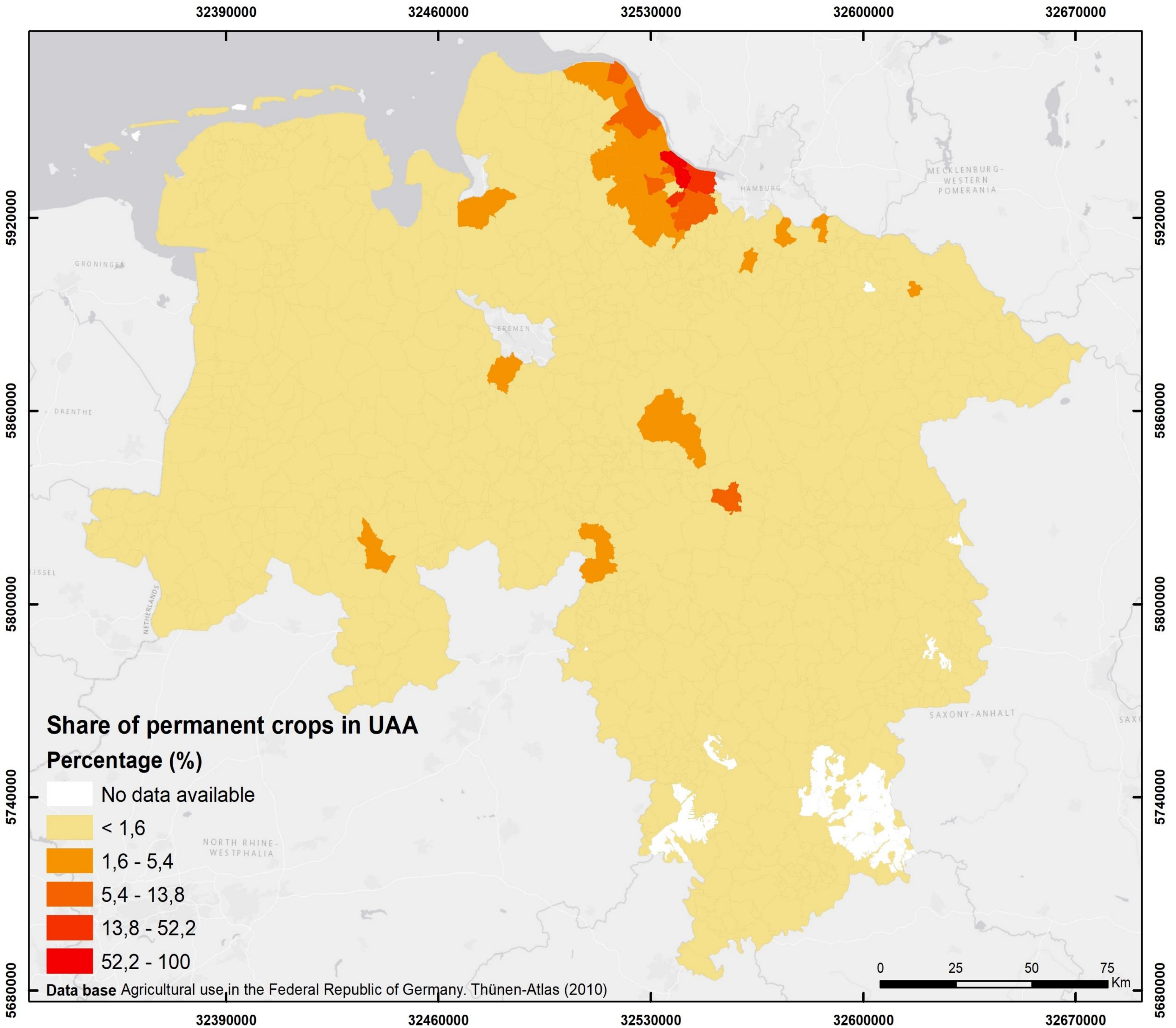

Republished from [7] under a CC BY license, with permission from the Thünen-Atlas (Collection of agricultural data from Germany), original copyright 2010. Based on the administrative units from the German Federal Agency for Cartography and Geodesy © GeoBasis-DE / BKG (2017) [2].

q. Livestock density

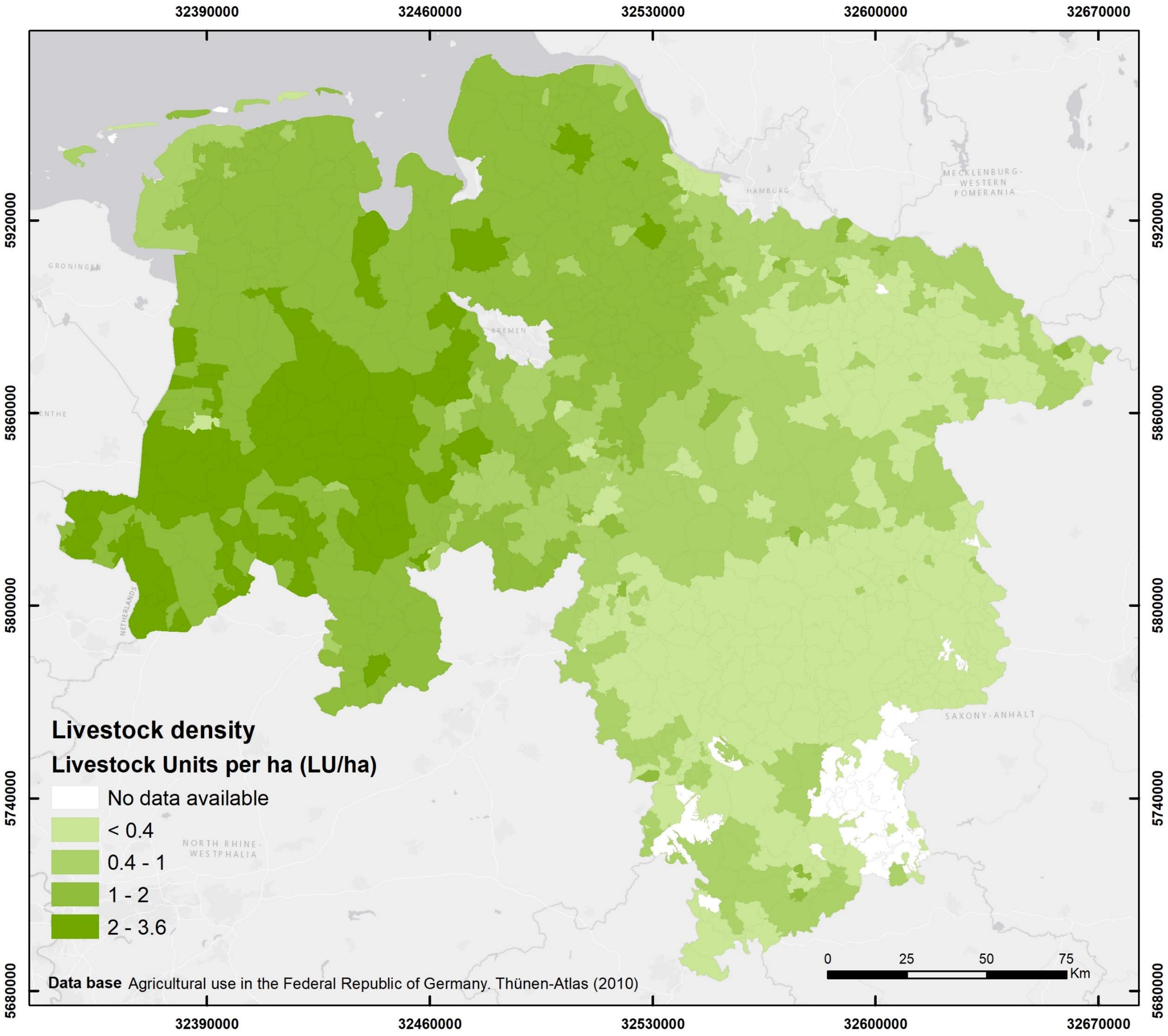

Republished from [7] under a CC BY license, with permission from the Thünen-Atlas (Collection of agricultural data from Germany), original copyright 2010. Based on the administrative units from the German Federal Agency for Cartography and Geodesy © GeoBasis-DE / BKG (2017) [2].

r. Soil Organic Carbon

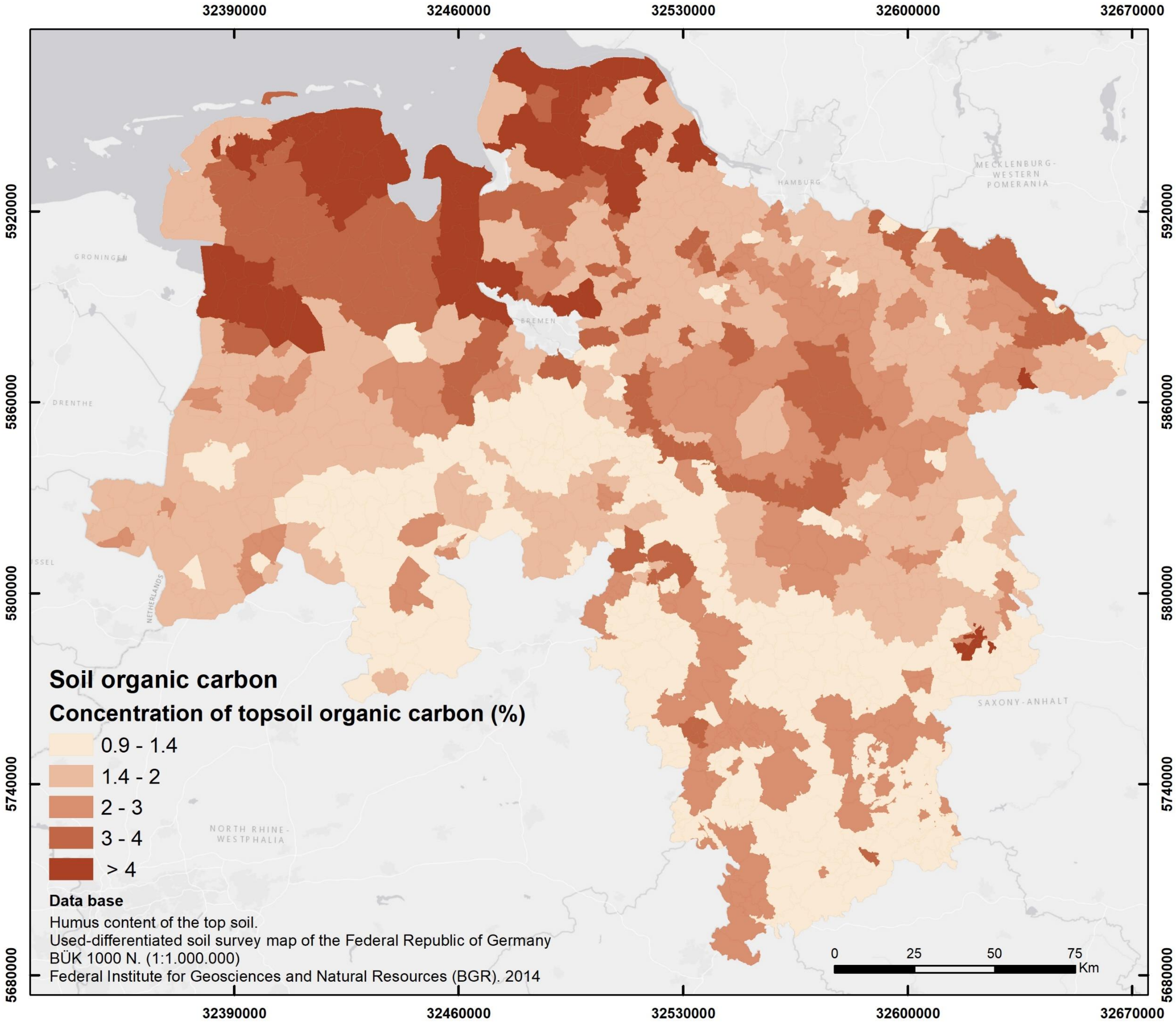

Republished from [4] under a CC BY licence, with permission from the German Federal Institute of Geosciences and Natural Resources BÜK1000 V2.1, © BGR, original copyright 2013. Based on data from the Deutscher Wetterdienst Climate Data Center (CDC OpenData) [3] and the administrative units from the German Federal Agency for Cartography and Geodesy © GeoBasis-DE / BKG (2017) [2].

s. Soil erodibility

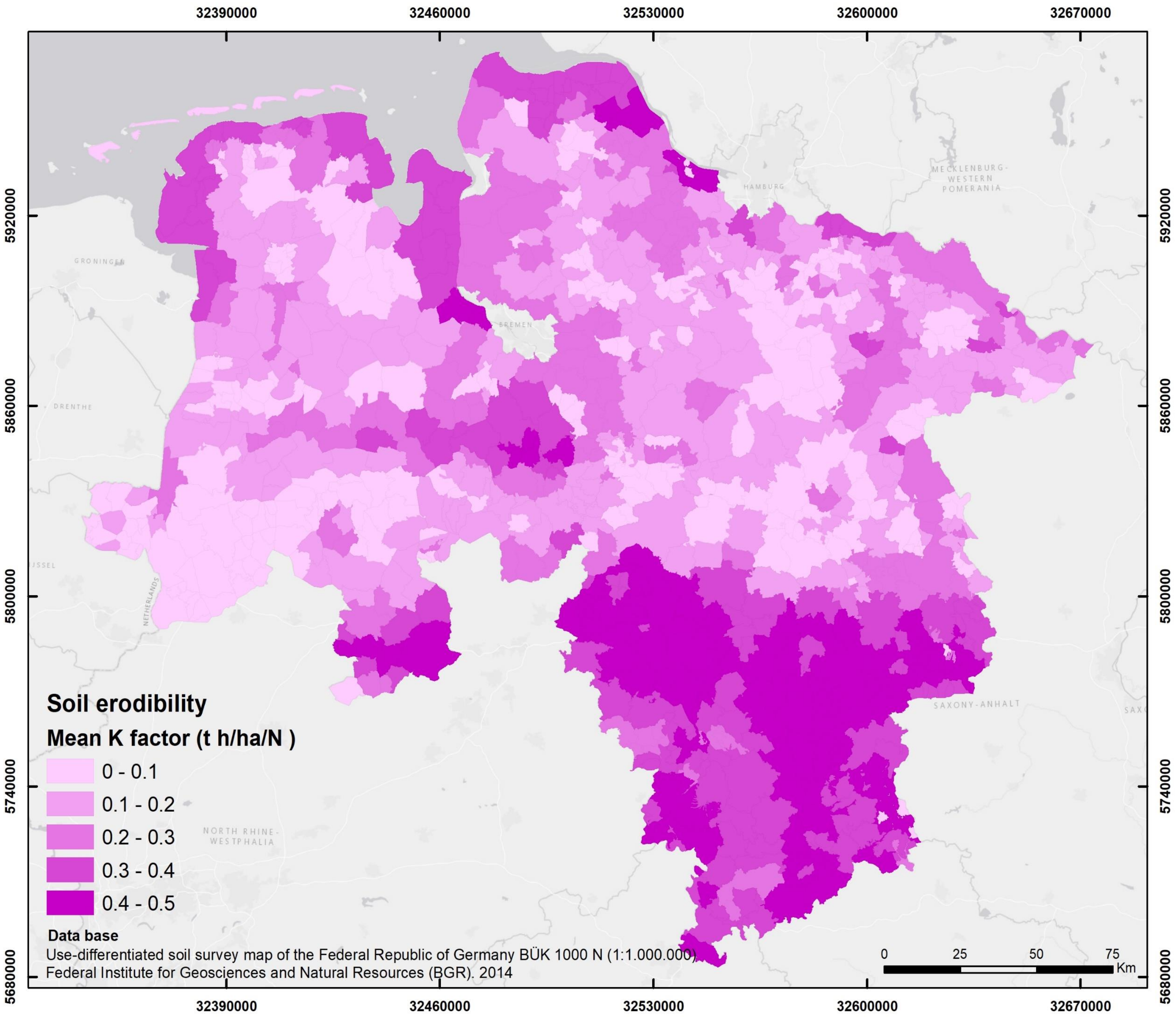

Republished from [4] under a CC BY licence, with permission from the German Federal Institute of Geosciences and Natural Resources BÜK1000 V2.1, © BGR, original copyright 2013. Based on data from the Deutscher Wetterdienst Climate Data Center (CDC OpenData) [3] and the administrative units from the German Federal Agency for Cartography and Geodesy © GeoBasis-DE / BKG (2017) [2].

t. Bulk density

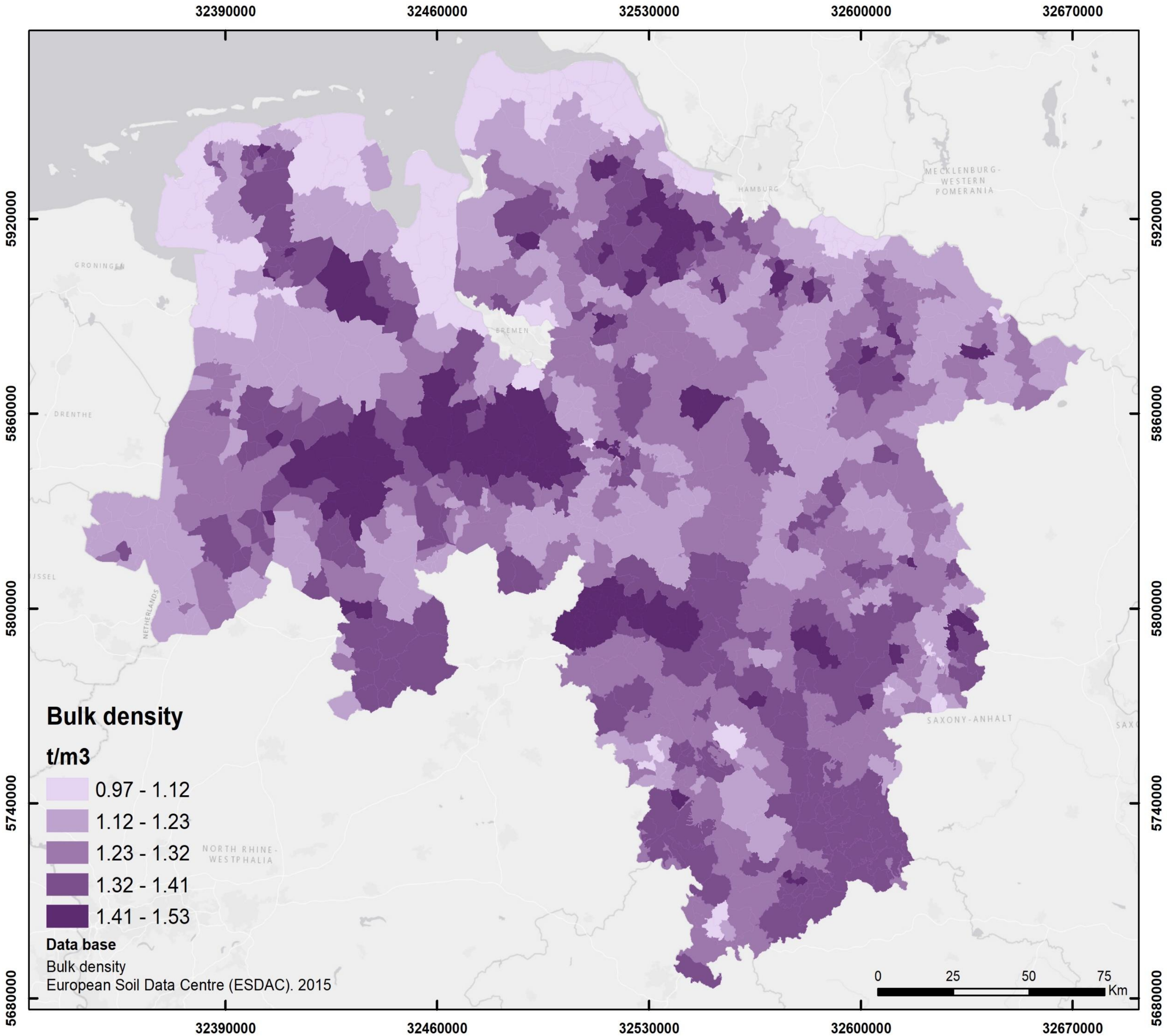

Republished from [8] under a CC BY license, with permission from the European Soil Data Centre (ESDAC). Based on the administrative units from the German Federal Agency for Cartography and Geodesy© GeoBasis-DE / BKG (2017) [2].

u. Soil erosion risk

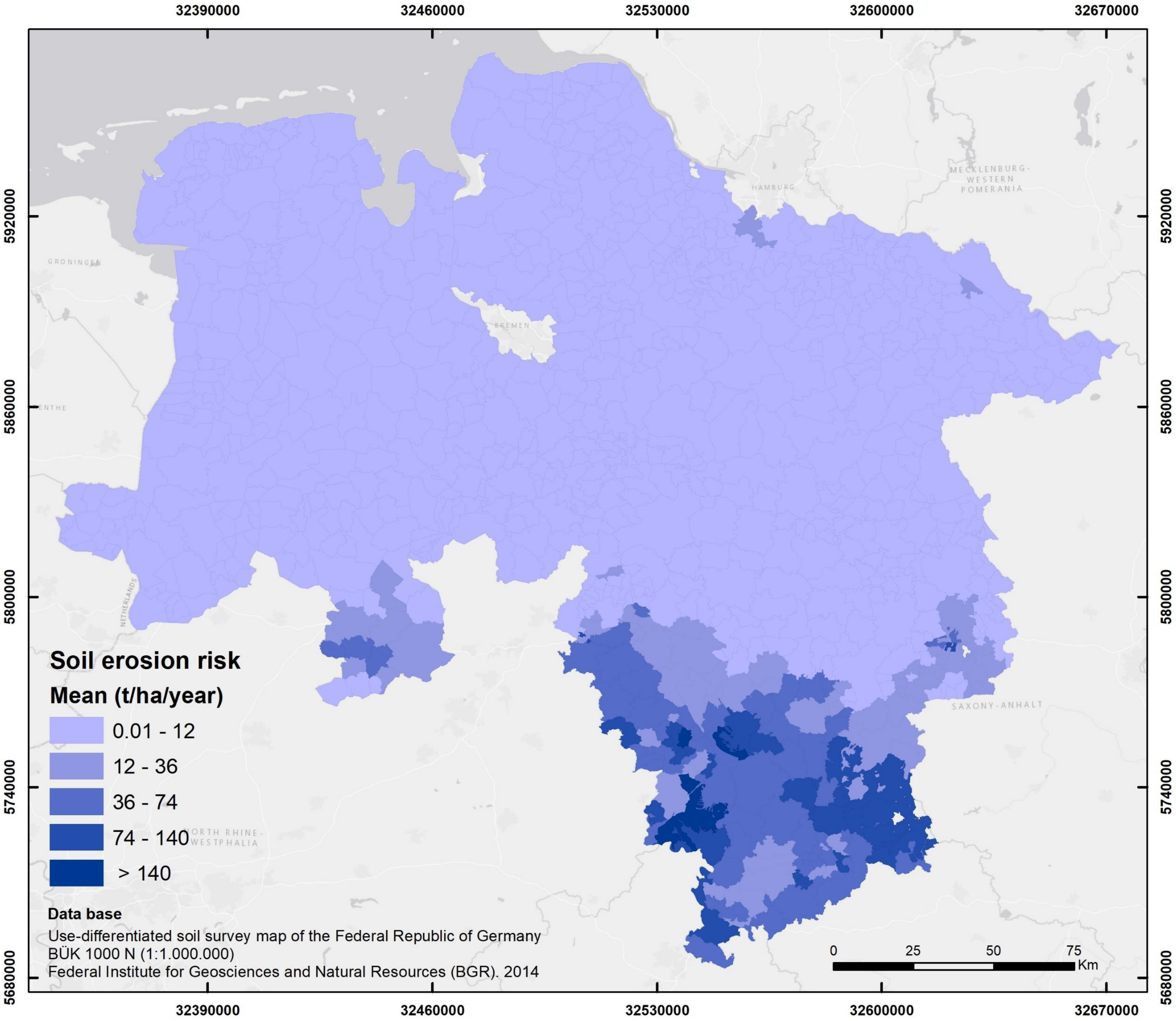

Republished from [4] under a CC BY licence, with permission from the German Federal Institute of Geosciences and Natural Resources BÜK1000 V2.1, © BGR, original copyright 2013. Based on data from the Deutscher Wetterdienst Climate Data Center (CDC OpenData) [3] and the administrative units from the German Federal Agency for Cartography and Geodesy © GeoBasis-DE / BKG (2017) [2].

v. Prevented soil erosion

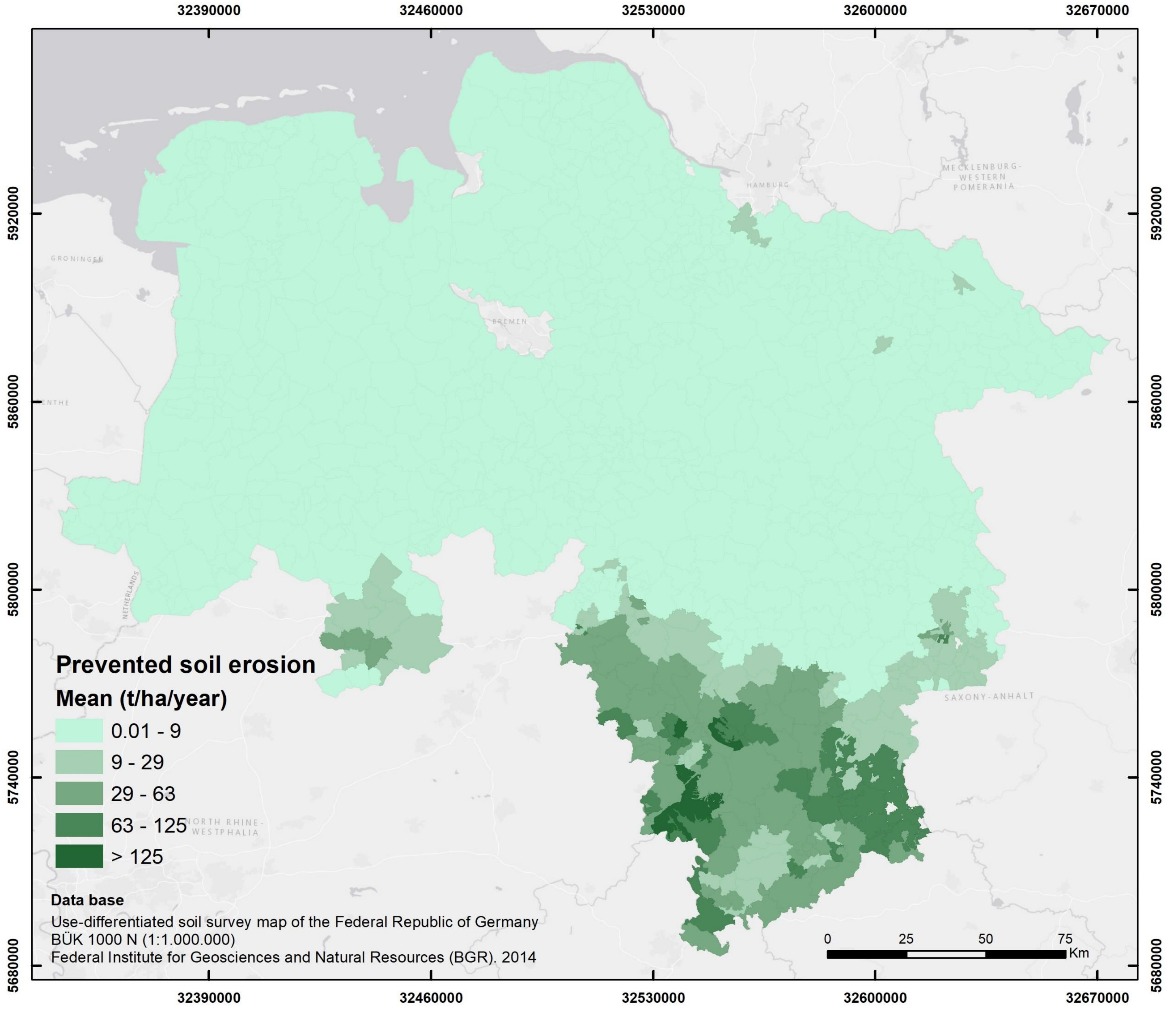

Republished from [4] under a CC BY licence, with permission from the German Federal Institute of Geosciences and Natural Resources BÜK1000 V2.1, © BGR, original copyright 2013. Based on data from the Deutscher Wetterdienst Climate Data Center (CDC OpenData) [3] and the administrative units from the German Federal Agency for Cartography and Geodesy © GeoBasis-DE / BKG (2017) [2].

w. Provision capacity

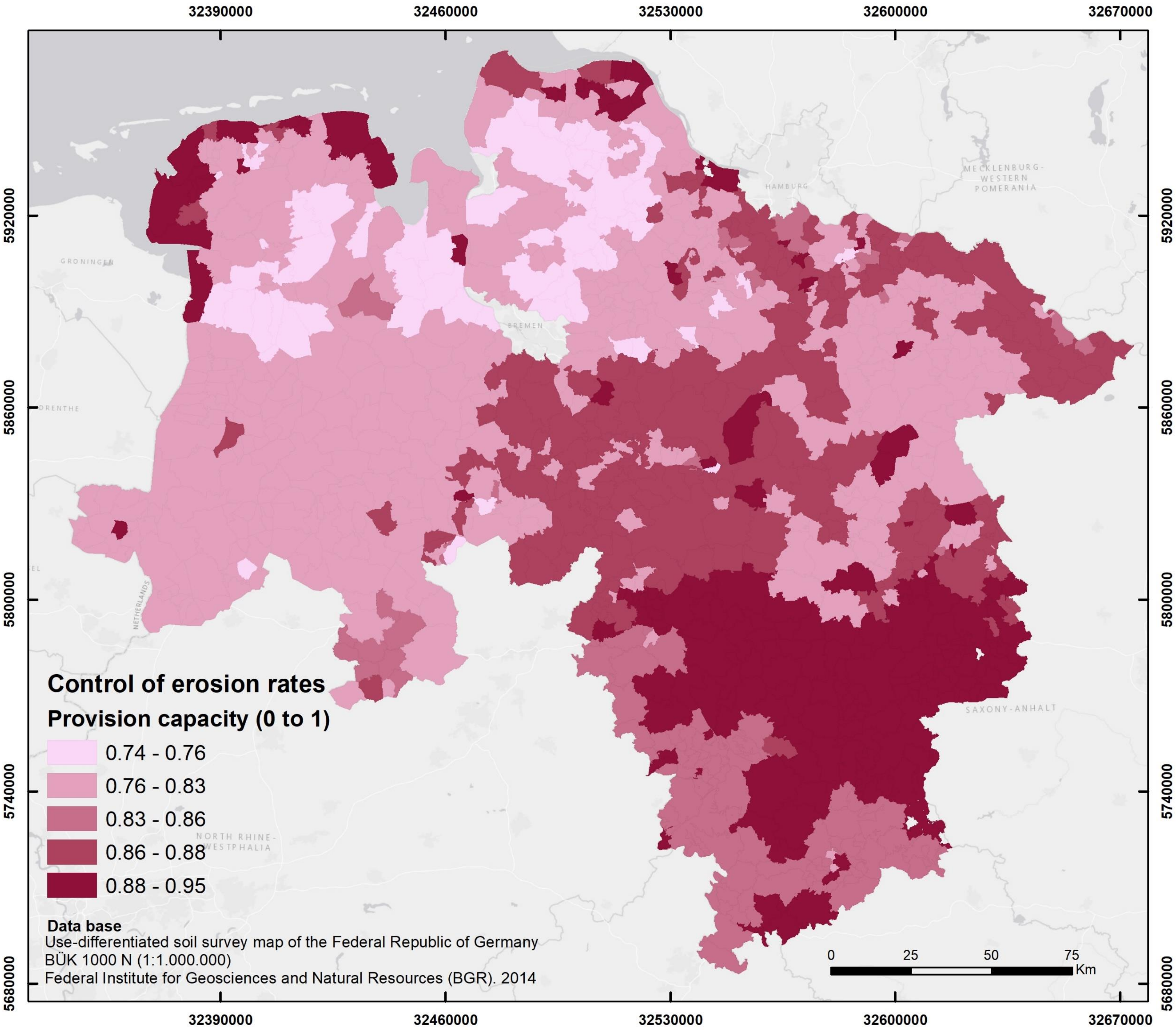

Republished from [4] under a CC BY licence, with permission from the German Federal Institute of Geosciences and Natural Resources BÜK1000 V2.1, © BGR, original copyright 2013. Based on data from the Deutscher Wetterdienst Climate Data Center (CDC OpenData) [3] and the administrative units from the German Federal Agency for Cartography and Geodesy © GeoBasis-DE / BKG (2017) [2].

(a) Overlap between provision capacity and condition

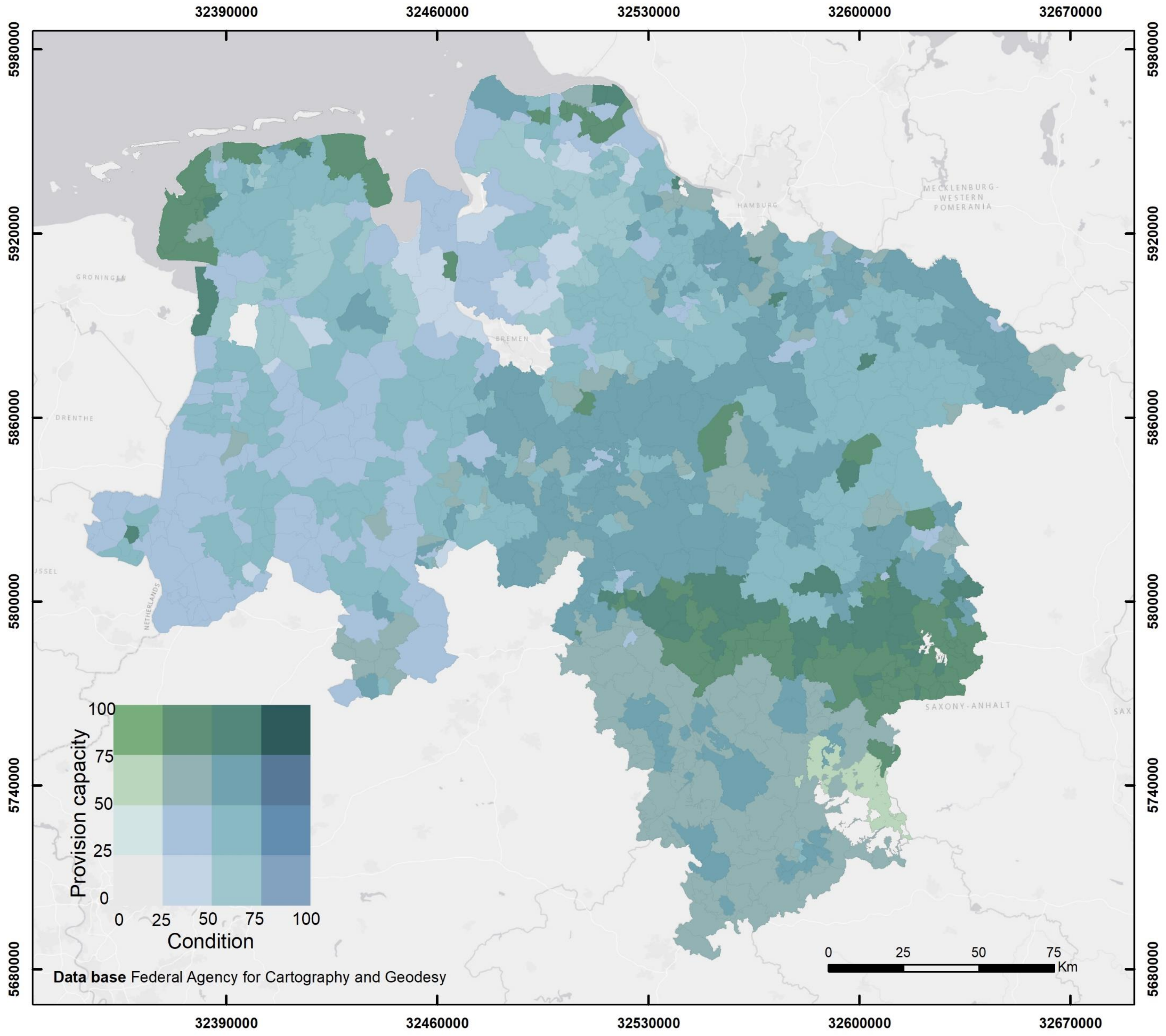

Based on data from the administrative units from the German Federal Agency for Cartography and Geodesy © GeoBasis-DE / BKG (2017) [2].

(b) Overlap between provision capacity and pressures

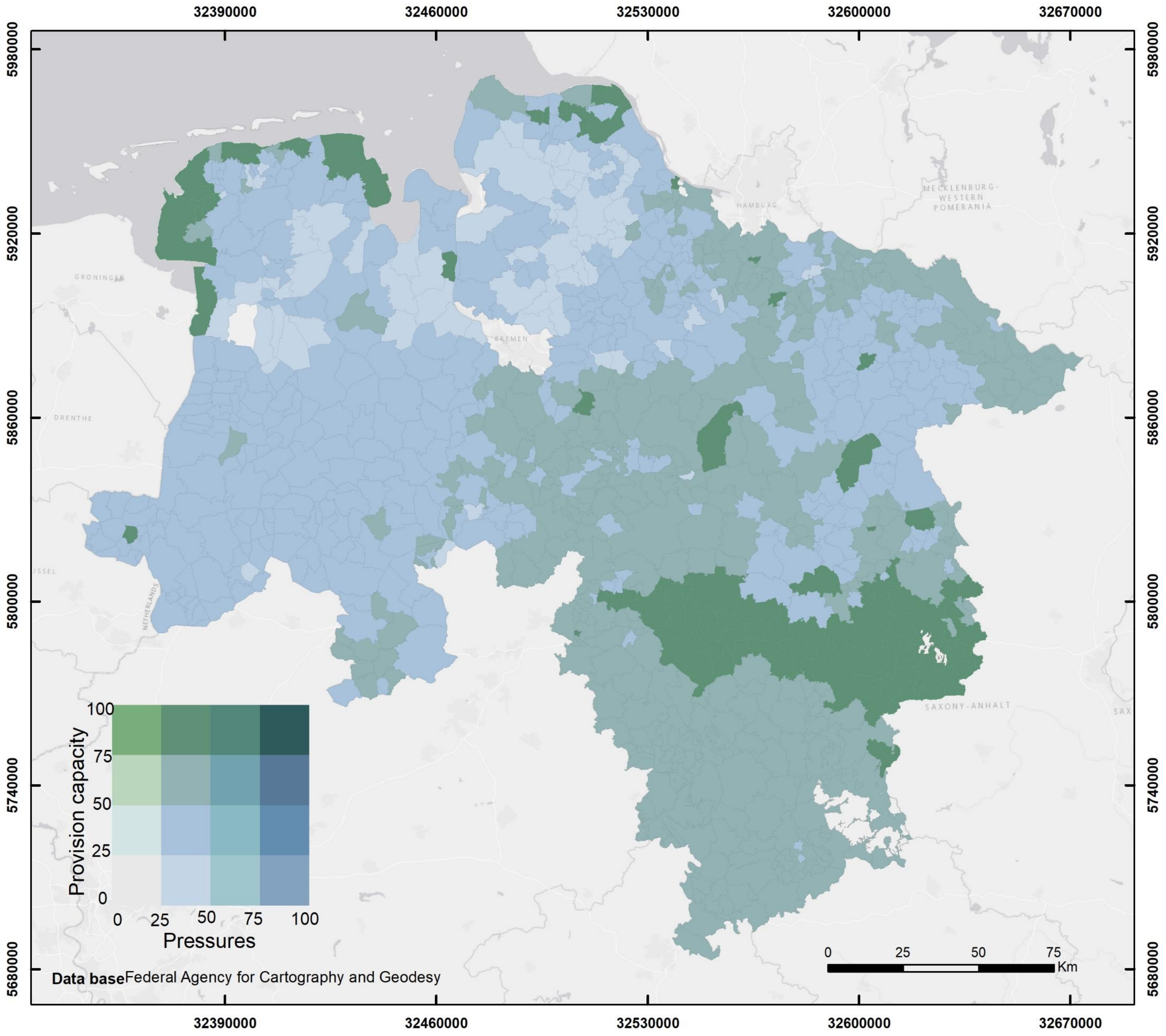

Based on data from the administrative units from the German Federal Agency for Cartography and Geodesy © GeoBasis-DE / BKG (2017) [2].

(a) Overlap between soil erosion risk, condition and provision capacity

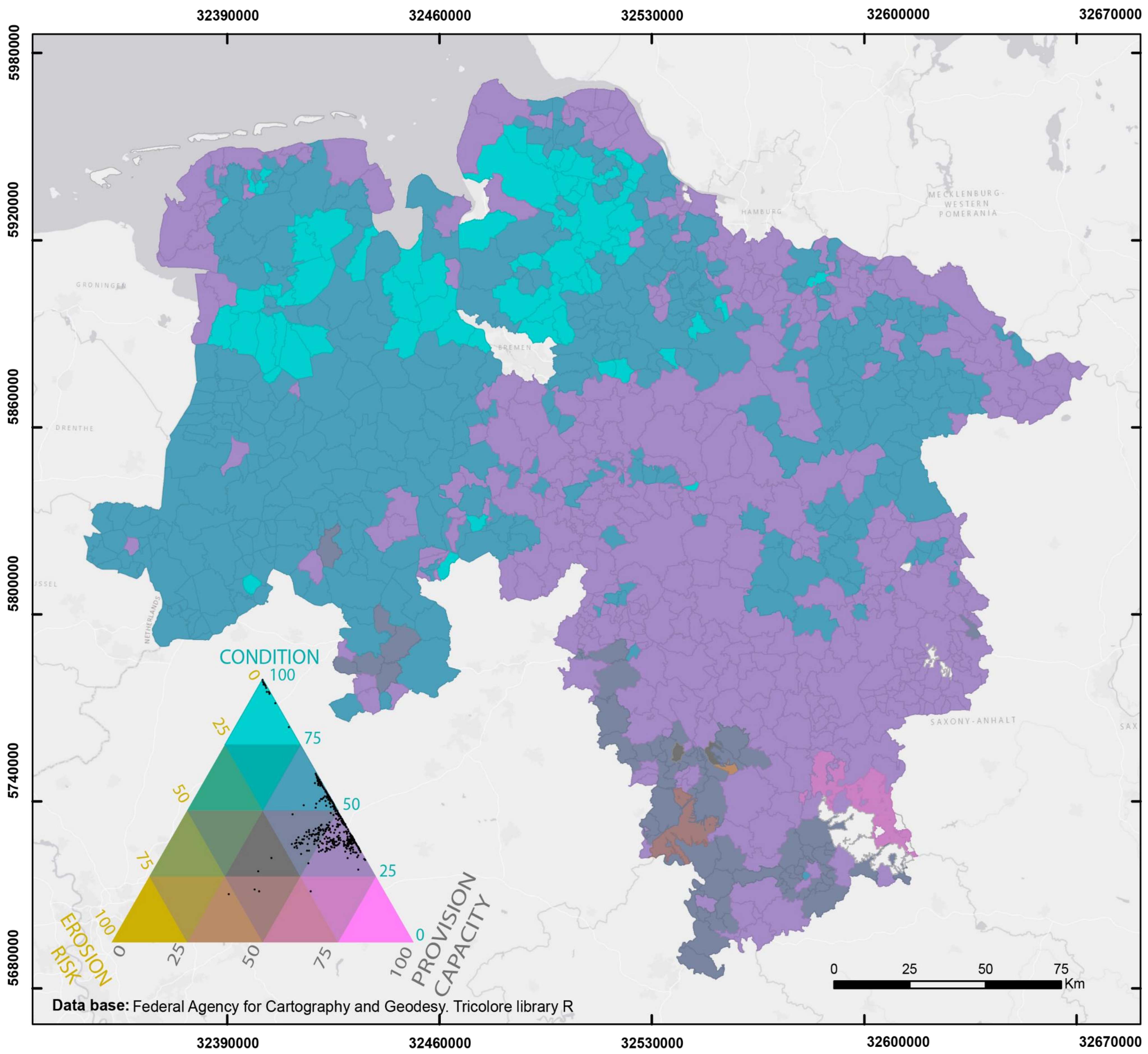

Based on data from the administrative units from the German Federal Agency for Cartography and Geodesy © GeoBasis-DE / BKG (2017) [2].

(b) Overlap between pressures, condition and provision capacity.

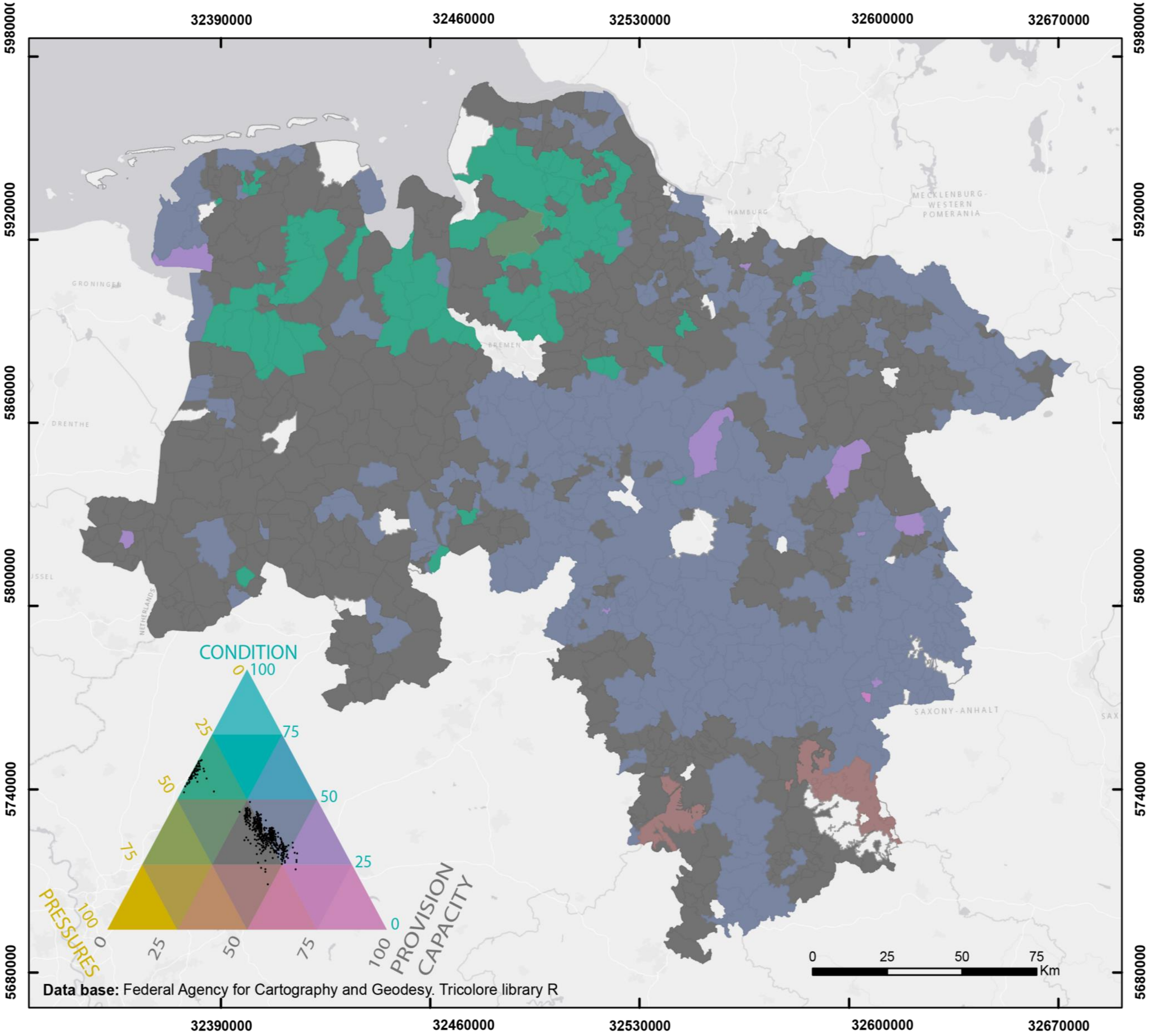

Based on data from the administrative units from the German Federal Agency for Cartography and Geodesy © GeoBasis-DE / BKG (2017) [2].

Supporting information S3 - Maps.

References

1. European Environment Agency. Land Cover Change (LCC) 2006-2012 [Internet]. 2019 [cited 2019 Jul 31]. Available from: https://land.copernicus.eu/pan-european/corine-land-cover/lcc-2006-2012?tab=metadata

2. German Federal Agency for Cartography and Geodesy. Administrative areas 1: 250,000 (levels), as of 01.01. (VG250 01.01.) [Internet]. 2017 [cited 2019 Jun 17]. Available from: https://gdz.bkg.bund.de/index.php/default/digitale-geodaten/verwaltungsgebiete/verwaltungsgebiete-1-250-000-ebenen-stand-01-01-vg250-ebenen-01-01.html

3. Deutscher Wetterdienst. Climate Data Center (CDC OpenData) [Internet]. 2018 [cited 2018 Oct 8]. Available from: https://opendata.dwd.de/climate_environment/CDC/

4. Bundesanstalt für Geowissenschaften und Rohstoffe (BGR). Bodenübersichtskarte der Bundesrepublik Deutschland 1:1.000.000 [Internet]. 2019 [cited 2019 Apr 20]. Available from: https://produktcenter.bgr.de/terraCatalog/DetailResult.do?fileIdentifier=A95A723E-1274-4601-9E60-27079436F1F3

5. ESDAC. Pan-European SOC stock of agricultural soils [Internet]. 2014 [cited 2018 Oct 10]. Available from: https://esdac.jrc.ec.europa.eu/content/pan-european-soc-stock-agricultural-soils

6. SLA - LEA. Landentwicklung und Agrarförderung Niedersachsen LEA - Portal [Internet]. 2019 [cited 2019 Apr 3]. Available from: https://sla.niedersachsen.de/landentwicklung/LEA/

7. Gocht A, Röder N, Meyer-Borstel H. Thünen-Atlas: Landwirtschaftliche Nutzung (1999-2010) [Internet]. Braunschweig; 2014. Available from: https://gdi.thuenen.de/lr/agraratlas

8. ESDAC. Topsoil physical properties for Europe (based on LUCAS topsoil data) [Internet]. 2016 [cited 2019 Jul 17]. Available from: https://esdac.jrc.ec.europa.eu/content/topsoil-physical-properties-europe-based-lucas-topsoil-data
