# Supplementary material for: Assessment of the relationships between agroecosystem condition and the ecosystem service soil erosion regulation in Northern Germany
Source: PLoS One. 2020 Dec 7;15(12):e0234288. doi: 10.1371/journal.pone.0234288 (PMC7721136; doi:10.1371/journal.pone.0234288)
Supplement: Attachment — Submitted filename: Response_to_Reviewers.docx [file pone.0234288.s005.docx]

Ref: Assessment of the relationships between agroecosystem condition and the ecosystem service soil erosion regulation in Northern Germany

Dear Editor-in-chief,

We thank you, the academic editor and the reviewers, for taking the time to review our manuscript and for the constructive comments. We have taken into account your suggestions and are now submitting the revised version. This version includes the changes to the previous version of the manuscript highlighting the new sentences or crossing out the deleted ones. Please find below our responses to each of the comments made by the editor and the reviewers. We look forward to the result of the assessment of this new version.

Kind regards,

The authors

Decision on Manuscript PONE-D-20-15438

Ref: Assessment of the relationships between agroecosystem condition and the ecosystem service soil erosion regulation in Northern Germany

Thank you for submitting your manuscript to PLOS ONE. After careful consideration, we feel that it has merit but does not fully meet PLOS ONE’s publication criteria as it currently stands. Therefore, we invite you to submit a revised version of the manuscript that addresses the points raised during the review process.

Please revise the manuscript according to the suggestions of the referees, pay more attention to clarify the methods used and make the text more concise and easy to understand.

*Comments Editor*

| **Comments** | **Response** |
| --- | --- |
| Please consider the concerns of the referees and make a major revision on the manuscript, especially make clarifications on the methods used. Also, the descriptions of the text should be concise and the English should be polished by a native speaker. | Thank you for the suggestions to improve our manuscript. We hope we addressed them accordingly. A native speaker checked an improved the English language. |

*Comment reviewer 1*

| **Comment** | **Response** |
| --- | --- |
| The text is written in clear and understandable English, but it is a somewhat mechanical narrative, with lengthy paragraphs that could be edited for stile, making it a more pleasant reading without loosing any precision. E-publishing is not limited by the number of pages, but I do believe this paper should be more concise and avoid some obvious or redundant sentences. E.g. (lines 253-254): Manuscript - “The third step consists of the mapping of the ecosystem type agroecosystems that was previously identified.” Rewritten - The third step consisted in mapping previously identified agroecosystems. From 16 to 9 words in a sentence! I also recommend the submission of the whole text to a language reviewer, to avoid some simple mistakes like Line 261 “Begin of vegetation period”, which should be rewritten as “Beginning of vegetation period” (minor errors can be found throughout the manuscript. | Thank you for the recommendations, a native speaker has edited and revised the English. We realised that some sentences were a bit too technical and rather lengthy.  We corrected all the language errors you pointed to us directly as well as other mistakes. |
| Long established methods like the Universal Soil Loss Equation (USLE) may only be cited (with a bibliographic reference), there is no need to describe the formula. | Thank you for the suggestion, we moved the detailed description of the methods to the supporting information to make the paper more concise. We kept the explanation of the USLE equation to help to understand the methodology, particularly when describing the calculation of the control of erosion rates. |
| I can say the authors have done some robust geoprocessing analysis and that the paper has policy implications for soil conservation, identifying problematic municipalities where land is more prone to soil erosion and making recommendations to prevent the loss of ecosystem services. They also put the MAES framework to real world test at a regional scale, and suggest its improvement. | Thank you for noticing the work we have done. We hope we can contribute to improve the measurement of ecosystem condition at different scales and to contribute to better decision making in the context of MAES. |

*Comment reviewer 2*

| **Comment** | **Response** |
| --- | --- |
| The study applies the MAES framework for soiL erosion for the federal state Lower Syaxony in northern Germany. Generally, it is interesting to see a worked exampel oft he MAES framework, wever, the following methodoligcal shortfalls highlighted as following make the results not acceptable for publication at the moment. | Thank you for finding our work already interesting and for your suggestions to improve it. We hope we could address them all accordingly and that the article will be ready for publication soon. |
| Temporal and spatial mismatch of input data: A major issue is the considerable mismatch of temporal and (partially) spatial mismatch of scales. Aligning indicators (e.g., soil erosion parameters and indicators on land management for 2010 and habitat conversion for 2016-2017 combined with longterm climate data). It is likely difficult to obtain meaningful correlations between temporal misaligned datasets. One option could be to derive, e.g., the arithmetic mean for multiple points in time as welll that are currently only considered for a single year. Othwerwise, an assessment is needed how dynamic the selected parameters are over time to judge the effect temporal mismatches. Given the analysis at municipality level, the upscaling process of spatially explicit datasets to municipalities already creates some distortion, which equally affects the strength of relationships between the variables. | Thank you for pointing this out. We had to use different spatial and temporal data to calculate the indicators suggested in the MAES framework. We are aware that this brings some uncertainties to the study, but, to our knowledge, they were the only available data. Therefore we used them, although they are not the optimal desired data set. These points are reflected more in detail in the revised manuscript now.  One of the goals of our case study is to test the suggested MAES framework in a real-world situation and with the abundant data. We included a section in the discussion about the availability of data where we explained the limitations we faced regarding the accessibility of data and temporal and spatial mismatches. |
| In addition, I am not sure how you were able to draw conlusions on temporal changes on soil erosion risks given the use of single yeara for multiple input variables in your overview table. You only vary climate parameters, which leads for me tot he conlusion that you rather assess the hypothetical imapct on changes in climate parameters on soil erosion risk of a hypothetical taget year given the different years of input data. Generally, I would in this case rather aim for representative years for a dry or a wet year, or other different representative compbinations of input parameters. | The main variation in our assessment is that we now included the change in ecosystem types’ extent between the years 2006 and 2012 based on the CORINE change layers. This new calculation replaces the one we made before between the years 2016 and 2017. We hope that with this new calculation we can somehow address the temporal mismatches you raised. |
| Validation and calibration: I can hardly find any calibration or validation effort in the given dataset as done in other studies working on soil erosion rates. It would be necessary to do so to draw solid conclusions (e.g., https://www.pnas.org/content/110/29/12149, https://iopscience.iop.org/article/10.1088/1748-9326/11/3/034005/pdf). | Thank you for pointing this out and for the articles you suggested. Validation and calibration of soil erosion models can be generally done from plot to catchment scale (primarily small catchments) as sediment loads can be easily measured as outlets. However, at a regional scale such as Lower Saxony, the calibration of models like the USLE remains an unachieved goal in research. This is due to the time and workload required. Additionally, in our study area, we calculated the mean soil erosion losses for 30 years based on crop rotation for this period. To adequately calibrate the USLE for Lower Saxony, it would be necessary to measure data for the whole region for several years. Furthermore, we used the model already calibrated for German conditions using the approaches and data from Auerswald and Elhaus, 2013 and DIN – Deutsche Institut für Normung, 2017. |
