# Supplementary material for: Assessment of the relationships between agroecosystem condition and the ecosystem service soil erosion regulation in Northern Germany
Source: PLoS One. 2020 Dec 7;15(12):e0234288. doi: 10.1371/journal.pone.0234288 (PMC7721136; doi:10.1371/journal.pone.0234288)
Supplement: Peer review history

# Peer review: Decision Letter 0

**DOI:** 10.1371/journal.pone.0234288.r001

**Authors:** RunGuo Zang

9 Jul 2020

PONE-D-20-15438

Assessment of the relationships between agroecosystem condition and soil erosion regulating ecosystem service in Northern Germany

PLOS ONE

Dear Dr. Rendon,

Thank you for submitting your manuscript to PLOS ONE. After careful consideration, we feel that it has merit but does not fully meet PLOS ONE’s publication criteria as it currently stands. Therefore, we invite you to submit a revised version of the manuscript that addresses the points raised during the review process.

Please revise the manuscript according to the suggestions of the referees,pay more attention to clarify  the methods used and make the text more concise and easy to understand.

Please submit your revised manuscript by Aug 23 2020 11:59PM. If you will need more time than this to complete your revisions, please reply to this message or contact the journal office at plosone@plos.org. When you're ready to submit your revision, log on to https://www.editorialmanager.com/pone/ and select the 'Submissions Needing Revision' folder to locate your manuscript file.

Please include the following items when submitting your revised manuscript:

- A rebuttal letter that responds to each point raised by the academic editor and reviewer(s). You should upload this letter as a separate file labeled 'Response to Reviewers'.
- A marked-up copy of your manuscript that highlights changes made to the original version. You should upload this as a separate file labeled 'Revised Manuscript with Track Changes'.
- An unmarked version of your revised paper without tracked changes. You should upload this as a separate file labeled 'Manuscript'.

If you would like to make changes to your financial disclosure, please include your updated statement in your cover letter. Guidelines for resubmitting your figure files are available below the reviewer comments at the end of this letter.

If applicable, we recommend that you deposit your laboratory protocols in protocols.io to enhance the reproducibility of your results. Protocols.io assigns your protocol its own identifier (DOI) so that it can be cited independently in the future. For instructions see: http://journals.plos.org/plosone/s/submission-guidelines#loc-laboratory-protocols

We look forward to receiving your revised manuscript.

Kind regards,

RunGuo Zang

Academic Editor

PLOS ONE

Additional Editor Comments:

Please consider the concerns of the referees and make a major revision on the manuscript,especially make clarifications on the methods used.Also,the descriptions of the text should be concise and the English should be polished by a native speaker.

Journal Requirements:

When submitting your revision, we need you to address these additional requirements.

1. Please ensure that your manuscript meets PLOS ONE's style requirements, including those for file naming. The PLOS ONE style templates can be found at

https://journals.plos.org/plosone/s/file?id=wjVg/PLOSOne_formatting_sample_main_body.pdf and

https://journals.plos.org/plosone/s/file?id=ba62/PLOSOne_formatting_sample_title_authors_affiliations.pdf

2. We note that Figures 1, 5.1, 5.2, 5.3, 5.4, 5.7, 5.8 and S2 in your submission contain map images which may be copyrighted. All PLOS content is published under the Creative Commons Attribution License (CC BY 4.0), which means that the manuscript, images, and Supporting Information files will be freely available online, and any third party is permitted to access, download, copy, distribute, and use these materials in any way, even commercially, with proper attribution. For these reasons, we cannot publish previously copyrighted maps or satellite images created using proprietary data, such as Google software (Google Maps, Street View, and Earth). For more information, see our copyright guidelines: http://journals.plos.org/plosone/s/licenses-and-copyright.

We require you to either (1) present written permission from the copyright holder to publish these figures specifically under the CC BY 4.0 license, or (2) remove the figures from your submission:

2.1.    You may seek permission from the original copyright holder of Figures 1, 5.1, 5.2, 5.3, 5.4, 5.7, 5.8 and S2 to publish the content specifically under the CC BY 4.0 license.

We recommend that you contact the original copyright holder with the Content Permission Form (http://journals.plos.org/plosone/s/file?id=7c09/content-permission-form.pdf) and the following text:

“I request permission for the open-access journal PLOS ONE to publish XXX under the Creative Commons Attribution License (CCAL) CC BY 4.0 (http://creativecommons.org/licenses/by/4.0/). Please be aware that this license allows unrestricted use and distribution, even commercially, by third parties. Please reply and provide explicit written permission to publish XXX under a CC BY license and complete the attached form.”

Please upload the completed Content Permission Form or other proof of granted permissions as an "Other" file with your submission.

In the figure caption of the copyrighted figure, please include the following text: “Reprinted from [ref] under a CC BY license, with permission from [name of publisher], original copyright [original copyright year].”

2.2.    If you are unable to obtain permission from the original copyright holder to publish these figures under the CC BY 4.0 license or if the copyright holder’s requirements are incompatible with the CC BY 4.0 license, please either i) remove the figure or ii) supply a replacement figure that complies with the CC BY 4.0 license. Please check copyright information on all replacement figures and update the figure caption with source information. If applicable, please specify in the figure caption text when a figure is similar but not identical to the original image and is therefore for illustrative purposes only.

The following resources for replacing copyrighted map figures may be helpful:

USGS National Map Viewer (public domain): http://viewer.nationalmap.gov/viewer/

The Gateway to Astronaut Photography of Earth (public domain): http://eol.jsc.nasa.gov/sseop/clickmap/

Maps at the CIA (public domain): https://www.cia.gov/library/publications/the-world-factbook/index.html and https://www.cia.gov/library/publications/cia-maps-publications/index.html

NASA Earth Observatory (public domain): http://earthobservatory.nasa.gov/

Landsat: http://landsat.visibleearth.nasa.gov/

USGS EROS (Earth Resources Observatory and Science (EROS) Center) (public domain): http://eros.usgs.gov/#

Natural Earth (public domain): http://www.naturalearthdata.com/

[Note: HTML markup is below. Please do not edit.]

Reviewers' comments:

Reviewer's Responses to Questions

**Comments to the Author**

1. Is the manuscript technically sound, and do the data support the conclusions?

The manuscript must describe a technically sound piece of scientific research with data that supports the conclusions. Experiments must have been conducted rigorously, with appropriate controls, replication, and sample sizes. The conclusions must be drawn appropriately based on the data presented. 

Reviewer #1: Yes

Reviewer #2: No

**********

2. Has the statistical analysis been performed appropriately and rigorously? 

Reviewer #1: N/A

Reviewer #2: Yes

**********

3. Have the authors made all data underlying the findings in their manuscript fully available?

The PLOS Data policy requires authors to make all data underlying the findings described in their manuscript fully available without restriction, with rare exception (please refer to the Data Availability Statement in the manuscript PDF file). The data should be provided as part of the manuscript or its supporting information, or deposited to a public repository. For example, in addition to summary statistics, the data points behind means, medians and variance measures should be available. If there are restrictions on publicly sharing data—e.g. participant privacy or use of data from a third party—those must be specified.

Reviewer #1: Yes

Reviewer #2: Yes

**********

4. Is the manuscript presented in an intelligible fashion and written in standard English?

PLOS ONE does not copyedit accepted manuscripts, so the language in submitted articles must be clear, correct, and unambiguous. Any typographical or grammatical errors should be corrected at revision, so please note any specific errors here.

Reviewer #1: Yes

Reviewer #2: Yes

**********

5. Review Comments to the Author

Please use the space provided to explain your answers to the questions above. You may also include additional comments for the author, including concerns about dual publication, research ethics, or publication ethics. (Please upload your review as an attachment if it exceeds 20,000 characters)

Reviewer #1: The text is written in clear and understandable English, but it is a somewhat mechanical narrative, with lengthy paragraphs that could be edited for stile, making it a more pleasant reading without loosing any precision. E-publishing is not limited by the number of pages, but I do believe this paper should be more concise and avoid some obvious or redundant sentences. E.g. (lines 253-254):

Manuscript - “The third step consists of the mapping of the ecosystem type agroecosystems that was previously identified.”

Rewritten - The third step consisted in mapping previously identified agroecosystems.

From 16 to 9 words in a sentence!

I also recommend the submission of the whole text to a language reviewer, to avoid some simple mistakes like Line 261 “Begin of vegetation period”, which should be rewritten as “Beginning of vegetation period” (minor errors can be found throughout the manuscript.

Long established methods like the Universal Soil Loss Equation (USLE) may only be cited (with a bibliographic reference), there is no need to describe the formula.

I can say the authors have done some robust geoprocessing analysis and that the paper has policy implications for soil conservation, identifying problematic municipalities where land is more prone to soil erosion and making recommendations to prevent the loss of ecosystem services. They also put the MAES framework to real world test at a regional scale, and suggest its improvement.

Reviewer #2: The study applies the MAES framework for soiL erosion for the federal state Lower Syaxony in northern Germany. Generally, it is interesting to see a worked exampel oft he MAES framework, wever, the following methodoligcal shortfalls highlighted as following make the results not acceptable for publication at the moment.

Temporal and spatial mismatch of input data:

A major issue is the considerable mismatch of temporal and (partially) spatial mismatch of scales. Aligning indicators (e.g., soil erosion parameters and indicators on land management for 2010 and habitat conversion for 2016-2017 combined with longterm climate data). It is likely difficult to obtain meaningful correlations between temporal misaligned datasets. One option could be to derive, e.g., the arithmetic mean for multiple points in time as welll that are currently only considered for a single year. Othwerwise, an assessment is needed how dynamic the selected parameters are over time to judge the effect temporal mismatches. Given the analysis at municipality level, the upscaling process of spatially explicit datasets to municipalities already creates some distortion, which equally affects the strength of relationships between the variables.

In addition, I am not sure how you were able to draw conlusions on temporal changes on soil erosion risks given the use of single yeara for multiple input variables in your overview table. You only vary climate parameters, which leads for me tot he conlusion that you rather assess the hypothetical imapct on changes in climate parameters on soil erosion risk of a hypothetical taget year given the different years of input data. Generally, I would in this case rather aim for representative years for a dry or a wet year, or other different representative compbinations of input parameters.

Validation and calibration:

I can hardly find any calibration or validation effort in the given dataset as done in other studies working on soil erosion rates. It would be necessary to do so to draw solid conclusions

(e.g., https://www.pnas.org/content/110/29/12149, https://iopscience.iop.org/article/10.1088/1748-9326/11/3/034005/pdf).

**********

6. PLOS authors have the option to publish the peer review history of their article (what does this mean?). If published, this will include your full peer review and any attached files.

If you choose “no”, your identity will remain anonymous but your review may still be made public.

**Do you want your identity to be public for this peer review?** For information about this choice, including consent withdrawal, please see our Privacy Policy.

Reviewer #1: **Yes: **Maurício Schneider

Reviewer #2: No

[NOTE: If reviewer comments were submitted as an attachment file, they will be attached to this email and accessible via the submission site. Please log into your account, locate the manuscript record, and check for the action link "View Attachments". If this link does not appear, there are no attachment files.]

While revising your submission, please upload your figure files to the Preflight Analysis and Conversion Engine (PACE) digital diagnostic tool, https://pacev2.apexcovantage.com/. PACE helps ensure that figures meet PLOS requirements. To use PACE, you must first register as a user. Registration is free. Then, login and navigate to the UPLOAD tab, where you will find detailed instructions on how to use the tool. If you encounter any issues or have any questions when using PACE, please email PLOS at figures@plos.org. Please note that Supporting Information files do not need this step.

# Author comment: Author response to Decision Letter 0

**DOI:** 10.1371/journal.pone.0234288.r002

17 Nov 2020

Ref: Assessment of the relationships between agroecosystem condition and the ecosystem service soil erosion regulation in Northern Germany

Dear Editor-in-chief, 

We thank you, the academic editor and the reviewers, for taking the time to review our manuscript and for the constructive comments. We have taken into account your suggestions and are now submitting the revised version. This version includes the changes to the previous version of the manuscript highlighting the new sentences or crossing out the deleted ones. Please find below our responses to each of the comments made by the editor and the reviewers. We look forward to the result of the assessment of this new version.

Kind regards,

The authors

Decision on Manuscript PONE-D-20-15438

Ref: Assessment of the relationships between agroecosystem condition and the ecosystem service soil erosion regulation in Northern Germany

Thank you for submitting your manuscript to PLOS ONE. After careful consideration, we feel that it has merit but does not fully meet PLOS ONE’s publication criteria as it currently stands. Therefore, we invite you to submit a revised version of the manuscript that addresses the points raised during the review process.

Please revise the manuscript according to the suggestions of the referees, pay more attention to clarify the methods used and make the text more concise and easy to understand.

Comments Editor

Comment

Please consider the concerns of the referees and make a major revision on the manuscript, especially make clarifications on the methods used. Also, the descriptions of the text should be concise and the English should be polished by a native speaker. 

Response

Thank you for the suggestions to improve our manuscript. We hope we addressed them accordingly. A native speaker checked an improved the English language.

Comment reviewer 1

Comment

The text is written in clear and understandable English, but it is a somewhat mechanical narrative, with lengthy paragraphs that could be edited for stile, making it a more pleasant reading without loosing any precision. E-publishing is not limited by the number of pages, but I do believe this paper should be more concise and avoid some obvious or redundant sentences. E.g. (lines 253-254):

Manuscript - “The third step consists of the mapping of the ecosystem type agroecosystems that was previously identified.”

Rewritten - The third step consisted in mapping previously identified agroecosystems.

From 16 to 9 words in a sentence!

I also recommend the submission of the whole text to a language reviewer, to avoid some simple mistakes like Line 261 “Begin of vegetation period”, which should be rewritten as “Beginning of vegetation period” (minor errors can be found throughout the manuscript. Thank you for the recommendations, a native speaker has edited and revised the English. We realised that some sentences were a bit too technical and rather lengthy.

Response

We corrected all the language errors you pointed to us directly as well as other mistakes.

Comment

Long established methods like the Universal Soil Loss Equation (USLE) may only be cited (with a bibliographic reference), there is no need to describe the formula.

Response

Thank you for the suggestion, we moved the detailed description of the methods to the supporting information to make the paper more concise. We kept the explanation of the USLE equation to help to understand the methodology, particularly when describing the calculation of the control of erosion rates.

Comment

I can say the authors have done some robust geoprocessing analysis and that the paper has policy implications for soil conservation, identifying problematic municipalities where land is more prone to soil erosion and making recommendations to prevent the loss of ecosystem services. They also put the MAES framework to real world test at a regional scale, and suggest its improvement.

Response

Thank you for noticing the work we have done. We hope we can contribute to improve the measurement of ecosystem condition at different scales and to contribute to better decision making in the context of MAES.

Comment reviewer 2

Comment

The study applies the MAES framework for soiL erosion for the federal state Lower Syaxony in northern Germany. Generally, it is interesting to see a worked exampel oft he MAES framework, wever, the following methodoligcal shortfalls highlighted as following make the results not acceptable for publication at the moment.

Response

Thank you for finding our work already interesting and for your suggestions to improve it. We hope we could address them all accordingly and that the article will be ready for publication soon.

Comment

Temporal and spatial mismatch of input data:

A major issue is the considerable mismatch of temporal and (partially) spatial mismatch of scales. Aligning indicators (e.g., soil erosion parameters and indicators on land management for 2010 and habitat conversion for 2016-2017 combined with longterm climate data). It is likely difficult to obtain meaningful correlations between temporal misaligned datasets. One option could be to derive, e.g., the arithmetic mean for multiple points in time as welll that are currently only considered for a single year. Othwerwise, an assessment is needed how dynamic the selected parameters are over time to judge the effect temporal mismatches. Given the analysis at municipality level, the upscaling process of spatially explicit datasets to municipalities already creates some distortion, which equally affects the strength of relationships between the variables. 

Response

Thank you for pointing this out. We had to use different spatial and temporal data to calculate the indicators suggested in the MAES framework. We are aware that this brings some uncertainties to the study, but, to our knowledge, they were the only available data. Therefore we used them, although they are not the optimal desired data set. These points are reflected more in detail in the revised manuscript now.

One of the goals of our case study is to test the suggested MAES framework in a real-world situation and with the abundant data. We included a section in the discussion about the availability of data where we explained the limitations we faced regarding the accessibility of data and temporal and spatial mismatches.

Comment

In addition, I am not sure how you were able to draw conlusions on temporal changes on soil erosion risks given the use of single yeara for multiple input variables in your overview table. You only vary climate parameters, which leads for me tot he conlusion that you rather assess the hypothetical imapct on changes in climate parameters on soil erosion risk of a hypothetical taget year given the different years of input data. Generally, I would in this case rather aim for representative years for a dry or a wet year, or other different representative compbinations of input parameters.

Response

The main variation in our assessment is that we now included the change in ecosystem types’ extent between the years 2006 and 2012 based on the CORINE change layers. This new calculation replaces the one we made before between the years 2016 and 2017. We hope that with this new calculation we can somehow address the temporal mismatches you raised.

Comment

Validation and calibration:

I can hardly find any calibration or validation effort in the given dataset as done in other studies working on soil erosion rates. It would be necessary to do so to draw solid conclusions

(e.g., https://www.pnas.org/content/110/29/12149, https://iopscience.iop.org/article/10.1088/1748-9326/11/3/034005/pdf).

Response

Thank you for pointing this out and for the articles you suggested. Validation and calibration of soil erosion models can be generally done from plot to catchment scale (primarily small catchments) as sediment loads can be easily measured as outlets. However, at a regional scale such as Lower Saxony, the calibration of models like the USLE remains an unachieved goal in research. This is due to the time and workload required. Additionally, in our study area, we calculated the mean soil erosion losses for 30 years based on crop rotation for this period. To adequately calibrate the USLE for Lower Saxony, it would be necessary to measure data for the whole region for several years. Furthermore, we used the model already calibrated for German conditions using the approaches and data from Auerswald and Elhaus, 2013 and DIN – Deutsche Institut für Normung, 2017.

Attachment Submitted filename: Response_to_Reviewers.docx Click here for additional data file.

# Editor report: Decision Letter 1

**DOI:** 10.1371/journal.pone.0234288.r003

**Authors:** RunGuo Zang

20 Nov 2020

Assessment of the relationships between agroecosystem condition and the ecosystem service soil erosion regulation in Northern Germany

PONE-D-20-15438R1

Dear Dr. Rendon,

We’re pleased to inform you that your manuscript has been judged scientifically suitable for publication and will be formally accepted for publication once it meets all outstanding technical requirements.

Within one week, you’ll receive an e-mail detailing the required amendments. When these have been addressed, you’ll receive a formal acceptance letter and your manuscript will be scheduled for publication.

An invoice for payment will follow shortly after the formal acceptance. To ensure an efficient process, please log into Editorial Manager at http://www.editorialmanager.com/pone/, click the 'Update My Information' link at the top of the page, and double check that your user information is up-to-date. If you have any billing related questions, please contact our Author Billing department directly at authorbilling@plos.org.

If your institution or institutions have a press office, please notify them about your upcoming paper to help maximize its impact. If they’ll be preparing press materials, please inform our press team as soon as possible -- no later than 48 hours after receiving the formal acceptance. Your manuscript will remain under strict press embargo until 2 pm Eastern Time on the date of publication. For more information, please contact onepress@plos.org.

Kind regards,

RunGuo Zang

Academic Editor

PLOS ONE

Additional Editor Comments (optional):

Reviewers' comments:

# Editor report: Acceptance letter

**DOI:** 10.1371/journal.pone.0234288.r004

**Authors:** RunGuo Zang

25 Nov 2020

PONE-D-20-15438R1 

Assessment of the relationships between agroecosystem condition and the ecosystem service soil erosion regulation in Northern Germany 

Dear Dr. Rendon:

I'm pleased to inform you that your manuscript has been deemed suitable for publication in PLOS ONE. Congratulations! Your manuscript is now with our production department. 

If your institution or institutions have a press office, please let them know about your upcoming paper now to help maximize its impact. If they'll be preparing press materials, please inform our press team within the next 48 hours. Your manuscript will remain under strict press embargo until 2 pm Eastern Time on the date of publication. For more information please contact onepress@plos.org.

If we can help with anything else, please email us at plosone@plos.org. 

Thank you for submitting your work to PLOS ONE and supporting open access. 

Kind regards, 

PLOS ONE Editorial Office Staff

on behalf of

Professor RunGuo Zang 

Academic Editor

PLOS ONE
